# Exercise Training for Cerebrovascular and Cognitive Health in Adults at Risk of Cognitive Decline: A Scoping Review of Healthcare Translation and Evidence Gaps

**DOI:** 10.3390/healthcare14121774

**Published:** 2026-06-19

**Authors:** Kunrong Zhang, Yi-Chen Cheng, Chun-Hsien Su

**Affiliations:** 1 School of Physical Education, Guangdong University of Petrochemical Technology, Maoming 525000, China; zhangkunrong1215@gdupt.edu.cn; 2 Department of Business Administration, College of Management, Shih Chien University, Taipei 104336, Taiwan; yc.elaine@g2.usc.edu.tw; 3 Department of Physical Education, Putian University, Putian 351100, China; 4 Department of Exercise and Health Promotion, Chinese Culture University, Taipei 111369, Taiwan

**Keywords:** cognitive decline, dementia prevention, cerebrovascular function, exercise prescription, healthcare translation

## Abstract

**Highlights:**

**What are the main findings?**

- The evidence base for exercise, cerebrovascular regulation, and cognitive health remains uneven in evidence maturity, with stronger development in aerobic cerebrovascular research than in integrated vascular-cognitive intervention designs.
- Current studies often separate mechanistic cerebrovascular outcomes from clinically relevant cognitive outcomes, limiting the ability to determine whether exercise-induced vascular adaptations translate into cognitive benefit.

**What are the implications of the main findings?**

- The findings indicate that precision exercise prescription for cognitive health is not yet supported by sufficiently integrated evidence across exercise dose, vascular assessment, cognitive testing, adherence, safety, and feasibility.
- Future healthcare-oriented trials should prioritize integrated vascular-cognitive outcome assessment, standardized exercise-dose reporting, and implementation-relevant measures to strengthen translation into rehabilitation, community prevention, and dementia risk management.

**Abstract:**

**Background/Objectives**: Dementia and cognitive decline place increasing demands on healthcare systems, rehabilitation services, long-term care, and community-based prevention. Structured exercise training is a promising strategy for adults at risk of cognitive decline, but it remains unclear how intervention studies integrate cerebrovascular and cognitive outcomes in ways that can inform clinical translation, rehabilitation planning, and exercise prescription. **Methods**: This scoping review followed PRISMA-ScR guidance. PubMed/MEDLINE and Scopus were searched for peer-reviewed English-language studies published from 2010 to 2026, supplemented by reference list checking and citation chasing. Eligible studies were human intervention studies involving structured exercise training and at least one cerebrovascular, vascular, brain-related, or cognitive outcome. Studies were mapped by exercise modality, population risk profile, grouped outcome domain, and outcome-integration category. **Results**: Fifty-four studies were included. A central finding was the vascular cognitive integration gap: only 7 studies assessed both cerebrovascular and cognitive outcomes within the same intervention design, whereas 38 studies reported cognitive outcomes only and 9 reported cerebrovascular or vascular outcomes only. Aerobic training formed the most developed evidence cluster for direct cerebrovascular outcomes, whereas other modalities were more often represented in cognition-focused studies but less frequently included direct cerebrovascular measures. **Conclusions**: Current evidence is limited by a major vascular cognitive integration gap. Because most exercise intervention studies separate cerebrovascular and cognitive outcomes, the field cannot yet determine whether exercise-induced cerebrovascular adaptations correspond to cognitive improvements in the same participants. Future trials should combine cerebrovascular assessment, domain-specific cognitive testing, dose reporting, adherence monitoring, safety reporting, feasibility evaluation, and mechanistic biomarkers to support more precise exercise prescription for dementia risk mitigation and cognitive health promotion.

## 1. Introduction

Cognitive decline and dementia risk represent major healthcare challenges, with increasing consequences for individuals, families, health systems, rehabilitation services, long-term care resources, and community-based prevention [1,2]. Although these challenges are especially visible in aging populations, the scope of this review was not restricted to older adults. Instead, the review focused on adults, older adults, sedentary or physically inactive adults, and populations with cognitive, vascular, cardiometabolic, or functional risk profiles relevant to cerebrovascular and cognitive health [2].

Structured exercise training is clinically attractive because it can be delivered across community, outpatient, rehabilitation, and preventive care settings while targeting multiple physiological and functional risk factors, including vascular function, metabolic regulation, inflammation, neurotrophic signaling, physical capacity, and daily functional independence [3,4,5]. Cerebrovascular dysfunction is one plausible pathway linking vascular risk, brain health, and cognitive decline [6,7]. Outcomes such as cerebral blood flow, cerebral perfusion, cerebral blood velocity, cerebrovascular reactivity, cerebral oxygenation, and neurovascular coupling may therefore provide mechanistic insight into how exercise affects cognitive health [4,8,9]. However, many exercise intervention studies assess cognition without measuring cerebrovascular adaptation, whereas others assess vascular or cerebrovascular outcomes without evaluating cognitive relevance. This separation limits the ability to determine whether exercise-related vascular adaptations correspond with meaningful cognitive outcomes in the same participants [4]. To clarify the scope of outcomes, this review adopted an ICF-informed perspective without using the ICF as a formal coding framework. Cerebrovascular, vascular, and brain-related measures were treated primarily as body-function or body-structure-related outcomes that may help explain biological mechanisms. Cognitive outcomes, cognitive-motor performance, physical capacity, daily function, rehabilitation relevance, and community prevention were treated as functioning-oriented outcomes or healthcare translation contexts. This framing allowed mechanistic outcomes to be considered in relation to individual functioning rather than discussed as isolated biological endpoints. This methodological framing also explains why a scoping review was needed: the field has not only accumulated mechanistic and cognitive outcomes, but has done so using partially disconnected outcome frameworks that require explicit mapping before clinical translation can be judged.

Physical inactivity and vascular risk are modifiable contributors to cerebrovascular and cognitive vulnerability and are directly relevant to healthcare prevention and rehabilitation strategies. Sedentary behavior and insufficient physical activity are associated with reduced cardiorespiratory fitness, hypertension, insulin resistance, obesity, systemic inflammation, oxidative stress, and endothelial dysfunction [2,10]. These processes may impair peripheral and cerebral vascular regulation, thereby increasing vulnerability to cognitive decline [6,11]. Adults with mild cognitive impairment, subjective cognitive decline, cardiometabolic risk factors, or functional frailty may be particularly relevant target populations for exercise-based healthcare intervention because they represent groups in which brain health trajectories may still be modifiable [2,5]. However, these populations should not be treated as clinically interchangeable because they differ in baseline cognitive status, vascular burden, functional reserve, comorbidity profile, adherence needs, and expected responsiveness to exercise interventions. In addition, middle-aged and older adults with vascular or metabolic risk may experience cognitive vulnerability before the onset of clinically diagnosed dementia, indicating that preventive exercise strategies should not be limited to individuals with established cognitive impairment [6,11]. These baseline differences should be considered when interpreting cerebrovascular adaptation, cognitive outcome sensitivity, adherence, feasibility, and generalizability to clinical practice. A clearer mapping of studied populations, assessed cerebrovascular and cognitive outcomes, and tested exercise modalities is therefore needed to determine not only what evidence exists, but also whether the available evidence is sufficiently integrated across population risk profiles to inform healthcare-oriented exercise prescription [4,5].

**Figure 1:**
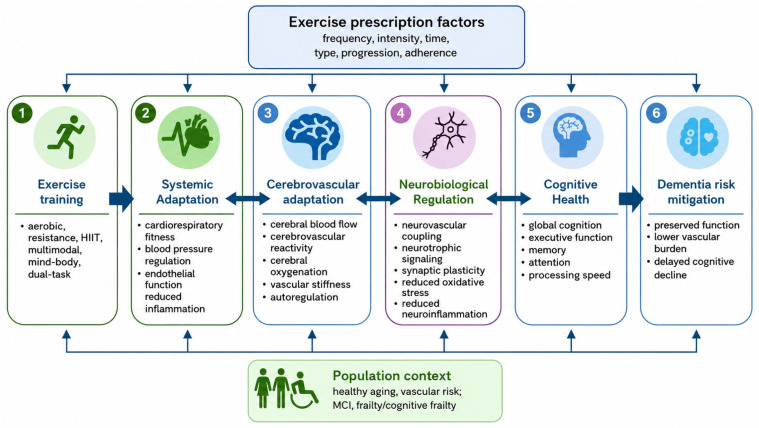
Conceptual framework linking structured exercise training, systemic adaptation, cerebrovascular adaptation, neurobiological regulation, cognitive health, and dementia risk mitigation. The framework is presented as an overall directional model from exercise exposure toward clinically relevant cognitive and preventive outcomes. However, selected relationships are shown as bidirectional to indicate that systemic adaptation, cerebrovascular adaptation, neurobiological regulation, and cognitive health may interact through partially overlapping pathways rather than through a strictly linear causal sequence. Exercise prescription factors and population context are presented as modifying influences that may shape intervention responsiveness, outcome relevance, and healthcare translation.

Exercise training is a multi-system intervention that may influence cerebrovascular and cognitive health through several overlapping pathways [3,4]. Aerobic exercise can improve cardiorespiratory fitness, endothelial function, blood pressure regulation, cerebral perfusion, and cerebrovascular responsiveness [4,5]. However, the current evidence base appears to be affected by modality concentration bias. Direct cerebrovascular outcomes have been examined mainly in aerobic or aerobic-based exercise studies, whereas resistance training, multimodal or multicomponent programs, mind–body exercise, and dual-task or exergaming paradigms are more often represented in cognition-focused studies without concurrent cerebrovascular assessment. Other exercise modalities may support cognitive health through strength, functional reserve, metabolic regulation, motor-cognitive integration, balance, self-regulation, or task-related brain activation, but their cerebrovascular relevance remains less consistently tested [5,12,13,14]. Despite this broad range of intervention approaches, the existing literature remains heterogeneous in exercise dose, program duration, supervision, adherence reporting, outcome selection, and participant risk profile [4,5]. As a result, it remains unclear which exercise modalities have been most frequently studied in relation to cerebrovascular outcomes, which modalities have been linked primarily to cognitive outcomes, and how often both domains are assessed within the same intervention design. This uncertainty limits the readiness of the evidence base for precision healthcare prescription and provides the methodological rationale for mapping exercise modalities against both cerebrovascular and cognitive outcome domains. In this review, precision exercise prescription refers to the ability to tailor exercise modality, dose, progression, supervision, and monitoring to an individual’s cognitive status, vascular or cardiometabolic risk burden, functional capacity, adherence needs, safety profile, and intended cerebrovascular or cognitive outcome target, rather than to genotype-based personalization. Figure 1 summarizes the overall conceptual framework linking exercise training, systemic and cerebrovascular adaptation, neurobiological regulation, cognitive health, and dementia risk mitigation.

A scoping review and evidence map are appropriate for this field because the evidence base is broad, heterogeneous, and mechanistically fragmented. Unlike a meta-analysis, which requires sufficient homogeneity in populations, interventions, outcomes, and effect estimates, a scoping review can clarify how evidence is distributed and where integration gaps limit healthcare translation [15,16]. The novelty of this review lies in its outcome-integration and healthcare-translation framework. Rather than grouping studies only by exercise modality or cognitive outcome, we classified studies according to whether they assessed cerebrovascular outcomes only, cognitive outcomes only, or both domains within the same intervention design. Therefore, this scoping review aimed to map evidence on structured exercise training, cerebrovascular outcomes, and cognitive health in adults and populations at elevated risk of cognitive decline, with emphasis on exercise modality, grouped outcome domains, outcome-integration category, and implementation-relevant gaps for healthcare-oriented exercise prescription.

## 2. Materials and Methods

### 2.1. Study Design and Reporting Framework

This scoping review was designed to map the breadth, characteristics, and translational gaps of the literature on structured exercise training, cerebrovascular function, and cognitive health in adults and populations at elevated risk of cognitive decline. A scoping review design was selected because this research area is characterized by substantial heterogeneity in exercise modalities, participant populations, intervention designs, cerebrovascular assessment methods, cognitive outcome domains, and healthcare-relevant implementation features. The review did not aim to estimate pooled intervention effects or determine comparative effectiveness. Instead, it aimed to clarify how the evidence is distributed, identify areas of research concentration, and highlight underrepresented intervention-outcome combinations that limit the development of healthcare-oriented exercise prescription for cognitive-risk and vascular-risk populations.

This review was conducted in accordance with the Preferred Reporting Items for Systematic Reviews and Meta-Analyses extension for Scoping Reviews, PRISMA-ScR [15]. Eligibility criteria, data charting, and evidence mapping were structured according to the Population, Concept, and Context framework, consistent with methodological guidance for scoping reviews [16]. The review protocol was retrospectively registered with the Open Science Framework on 29 May 2026 (registration link: https://osf.io/yp2rn/ (accessed on 16 June 2026), after completion of the final database search update on 4 May 2026 but before completion of the final evidence-map synthesis and manuscript revision. The registration was used to document the review objective, PCC-based eligibility criteria, search strategy, data charting framework, evidence-mapping approach, and descriptive synthesis methods used in this scoping review. Because registration occurred after the final search update, it should be interpreted as retrospective documentation of the review process rather than prospective protocol registration. This retrospective registration documents the review objective, PCC-based eligibility criteria, search strategy, data charting framework, evidence-mapping approach, and descriptive synthesis methods used in this scoping review. The Population component focused on adults, older adults, and populations at elevated risk of cognitive decline. The Concept component focused on repeated structured exercise training interventions. The Context component covered community, laboratory, outpatient, rehabilitation, health promotion, and aging-related prevention settings in which cerebrovascular or cognitive outcomes were assessed. In this review, repeated structured exercise training was operationalized as a planned exercise intervention delivered over more than one session, with an identifiable exercise modality, training frequency or duration, and an intended physiological, functional, cerebrovascular, or cognitive adaptation. This definition excluded acute single-session exercise studies, observational physical activity studies without a structured training intervention, and interventions in which exercise was not a primary or separable component.

Because the purpose of this review was to map evidence rather than test intervention efficacy, no meta-analysis was planned. Findings were synthesized descriptively and summarized visually through an evidence map. The evidence map was used to classify studies by exercise modality, population risk profile, grouped cerebrovascular outcome domain, grouped cognitive outcome domain, and outcome-integration category. Original study-level outcome labels were retained during data charting, and closely related outcomes were grouped into broader domains for visualization to improve interpretability and reduce excessive fragmentation in the evidence map. This framework was intended to identify not only where evidence is concentrated, but also where current reporting remains insufficient for precision healthcare prescription, including exercise dose, adherence, safety, feasibility, and vascular-cognitive outcome integration. The term evidence maturity was used descriptively to refer to the extent to which the available evidence showed transparent methodological and reporting features, exercise-dose reporting completeness, integration of cerebrovascular and cognitive outcomes, and implementation-relevant reporting such as adherence, attrition, safety, and feasibility. It was not used as a formal risk-of-bias rating, certainty-of-evidence judgment, or quantitative evidence grading system.

### 2.2. Eligibility Criteria Based on the PCC Framework

Eligibility criteria were defined using the Population, Concept, and Context (PCC) framework. The Population of interest included adults aged 18 years or older, with particular attention to middle-aged adults, older adults, sedentary or physically inactive adults, and adults at elevated risk of cognitive decline. Eligible risk groups included individuals with cognitive, vascular, cardiometabolic, or functional risk factors relevant to cerebrovascular or cognitive health. Examples included mild cognitive impairment, subjective cognitive decline, vascular cognitive impairment risk, hypertension, obesity, type 2 diabetes, metabolic syndrome, cardiovascular risk, frailty, cognitive frailty, and sedentary behavior. Studies involving children or adolescents only, animal or cell models, or exclusively elite athletic populations without relevance to cerebrovascular or cognitive health were excluded. The PCC framework and eligibility criteria are summarized in Table 1.

The detailed PCC framework, eligibility criteria, examples, and screening notes are provided in Supplementary Table S3. Original reported outcomes were used for eligibility assessment and data charting, whereas closely related outcomes were grouped into broader cerebrovascular and cognitive domains only after study inclusion for evidence-map visualization.

### 2.3. Information Sources

A systematic literature search was conducted in PubMed/MEDLINE and Scopus. These databases were selected to capture biomedical, clinical, neuroscience, rehabilitation, exercise science, and interdisciplinary health-related intervention literature relevant to structured exercise training, cerebrovascular function, and cognitive health. PubMed/MEDLINE was used to identify biomedical and clinical studies indexed with Medical Subject Headings and title/abstract terms, whereas Scopus was used to broaden coverage across interdisciplinary exercise science, rehabilitation, neuroscience, and health-related journals. The database search was supplemented by manual checking of reference lists from included studies and relevant review articles. Citation chasing was also conducted for key articles when necessary to identify additional eligible studies.

The search was limited to peer-reviewed full-text articles published in English between January 2010 and 4 May 2026, when the final database search update was completed. The January 2010 starting point was selected as a pragmatic date boundary to focus the review on contemporary exercise intervention research relevant to exercise neuroscience, cerebrovascular assessment, cognitive aging, rehabilitation, and exercise-based brain health promotion. This period was considered appropriate for capturing more recent intervention designs, structured exercise-prescription reporting practices, neuroimaging and cerebrovascular assessment methods, and cognitive-health-oriented exercise trials, without implying that earlier studies were unimportant. The complete database-specific search strategies are provided in Supplementary Table S1, and the search log, including database-specific search dates, is provided in Supplementary Table S2.

Database coverage was limited to PubMed/MEDLINE and Scopus because full institutional access to Web of Science, CINAHL, PsycINFO, and Embase was not available. To strengthen coverage within this constraint, the search used broad two-path strategies in both databases and was supplemented by manual reference list checking and citation chasing of key studies and relevant reviews. The potential implications of this database limitation are acknowledged in the Section 4.8 and considered when interpreting the evidence map.

### 2.4. Search Strategy

The search strategy was developed to reflect the PCC framework and to capture two complementary bodies of literature relevant to healthcare-oriented exercise prescription for cognitive-risk and vascular-risk adults. Two search paths were used in each database. Search Path A combined terms related to structured exercise training, cerebrovascular function, vascular regulation, and adult or aging-related populations. This path was designed to identify intervention studies assessing cerebrovascular or vascular outcomes relevant to brain health, including cerebral blood flow, cerebral perfusion, cerebrovascular reactivity, cerebral oxygenation, cerebral blood velocity, cerebral hemodynamics, cerebral autoregulation, and neurovascular coupling. Search Path B combined terms related to structured exercise training, cognitive outcomes, dementia, brain health, and aging-related contexts. This path was designed to identify cognition-focused exercise intervention studies in populations relevant to cognitive aging, mild cognitive impairment, dementia risk, or vascular-cognitive vulnerability.

Search terms were adapted for each database using database-specific syntax. In PubMed/MEDLINE, Medical Subject Headings and title/abstract terms were used. In Scopus, title, abstract, and keyword fields were searched using TITLE-ABS-KEY syntax. Exercise-related terms covered structured exercise training, physical exercise, aerobic exercise, resistance training, strength training, high-intensity interval training, combined training, multimodal or multicomponent exercise, Tai Chi, yoga, qigong, dual-task exercise, and coordinative or balance-oriented exercise. Cerebrovascular-related terms covered cerebral blood flow, brain perfusion, cerebral blood velocity, middle cerebral artery velocity, cerebrovascular reactivity, cerebral oxygenation, cerebral hemodynamics, cerebral autoregulation, neurovascular coupling, endothelial function, arterial stiffness, and vascular function when relevant to brain health or cognitive risk. Cognitive and dementia-related terms covered cognition, cognitive function, executive function, memory, attention, processing speed, cognitive decline, mild cognitive impairment, dementia, Alzheimer’s disease, brain aging, and brain health. These search terms were intentionally broad and were based on original outcome terminology used in the literature; closely related outcomes were grouped later during evidence-map coding and visualization.

Search filters were applied to restrict records to peer-reviewed articles published from 2010 to 2026 and in English. In PubMed/MEDLINE, the search was additionally limited to human studies. No restriction was applied based on exercise modality, intervention setting, or participant risk profile during the database search because the review aimed to map the breadth of structured exercise intervention evidence across adult and aging-related populations. Search results from each database and search path were documented in a search log, including database name, platform, search path, date searched, complete search syntax, filters applied, records retrieved, export format, and export file information. The complete database-specific search strategies are provided in Supplementary Table S1, and the search log is provided in Supplementary Table S2.

### 2.5. Screening and Study Selection Procedure

All records identified through database searching were exported and managed using Zotero 7.0 and Rayyan 1.4.3 [17]. The PubMed/MEDLINE search retrieved 12,924 records, including 4123 records from Search Path A and 8801 records from Search Path B. The Scopus search retrieved 27,189 records, including 11,310 records from Search Path A and 15,879 records from Search Path B. Overall, 40,113 records were identified through database searching.

**Figure 2:**
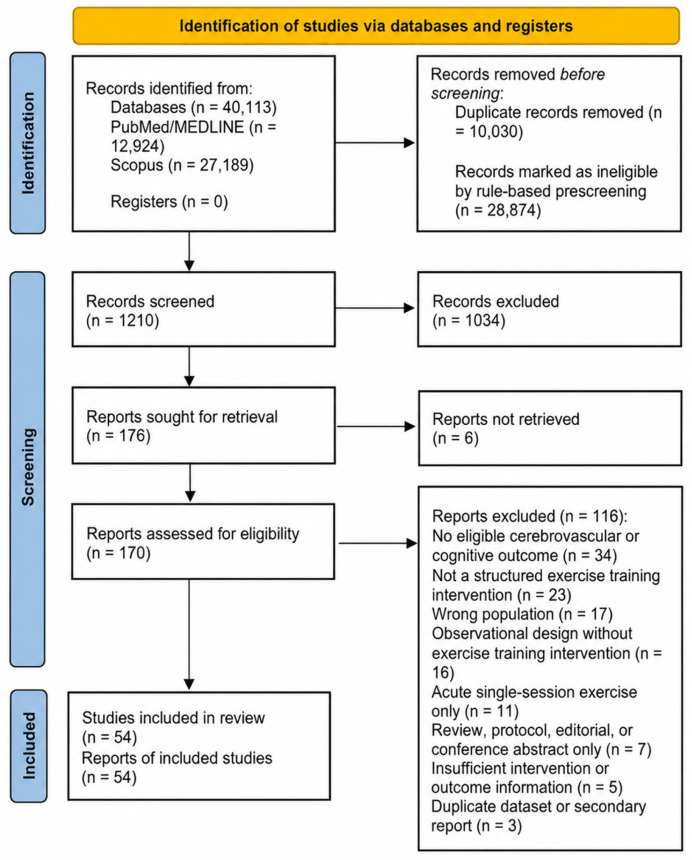
PRISMA-ScR flow diagram of study identification, screening, eligibility assessment, and inclusion. Records were identified from PubMed/MEDLINE and Scopus. A predefined PCC-based prescreening step was applied after duplicate removal, and records with uncertain eligibility were retained for title and abstract screening. A total of 54 studies were included in the descriptive synthesis and evidence map.

Records from each search path were exported as RIS files and deduplicated using bibliographic identifiers and citation metadata, including DOI, title, year, journal, pages, and author information. After the removal of 10,030 duplicate records, 30,084 records remained. The deduplicated RIS file was uploaded to Rayyan for screening. The study identification, deduplication, prescreening, title and abstract screening, full-text assessment, and inclusion process are summarized in Figure 2.

Screening criteria were predefined according to the PCC framework. At the title and abstract stage, records were marked as include, exclude, or maybe. Records were marked as included if they clearly involved adult or older adult participants, a structured exercise training intervention, and at least one originally reported cerebrovascular, vascular-risk, cognitive, dementia-related, or brain-health outcome. Records were excluded if they clearly involved non-human studies, children or adolescents only, acute single-session exercise, observational designs without an exercise training intervention, review articles, protocols, conference abstracts without outcome data, or no eligible cerebrovascular or cognitive outcome. Records were marked as maybe when eligibility could not be determined from the title or abstract. Titles, abstracts, and full texts were screened independently by two reviewers using the predefined PCC criteria. Disagreements were resolved through discussion, and uncertain records were retained for full-text assessment to preserve screening sensitivity. Percentage agreement and inter-rater reliability statistics were not calculated because the screening process was designed to apply the PCC-based eligibility criteria conservatively rather than to quantify reviewer agreement as an outcome of the review. Outcome grouping was not used as an eligibility filter; closely related outcomes were grouped only later during evidence-map coding and visualization.

Because the initial search strategy was intentionally broad and designed to maximize sensitivity across exercise, cerebrovascular, vascular-risk, cognitive, dementia-related, aging, and healthcare-related terms, a rule-based prescreening step was applied before title and abstract screening to improve feasibility while maintaining alignment with the predefined PCC framework. This step was used only to remove records that were clearly unrelated to the review question and was not based on intervention effects, outcome direction, statistical significance, or judgments about study quality. Records were retained for title and abstract screening when the title or abstract indicated an adult or aging-related population, a structured exercise or training intervention, and at least one cerebrovascular, vascular-risk, cognitive, dementia-related, or brain-health outcome. Records were excluded during prescreening only when they were clearly outside the PCC framework, including non-human studies, pediatric-only studies, non-intervention designs, acute single-session exercise studies, sedentary behavior or physical activity studies without a structured exercise-training intervention, peripheral vascular studies without brain-health or cognitive relevance, reviews, protocols, editorials, conference-only records, or records without any eligible exercise, cerebrovascular, vascular-risk, cognitive, dementia-related, or brain-health component. To reduce the risk of inappropriate exclusion, the prescreening rules were applied conservatively, records were retained whenever eligibility was uncertain, and uncertain cases were advanced to title and abstract screening rather than excluded at the prescreening stage. The full prescreening logic, including inclusion signals, exclusion signals, uncertainty rules, and examples of retained and excluded records, is provided in Supplementary Table S3.

Full texts were retrieved for all records marked as include or maybe after title and abstract screening. Full-text eligibility was assessed using the predefined inclusion and exclusion criteria. Reasons for full-text exclusion were recorded using standardized categories, including no structured exercise training intervention, acute single-session exercise only, wrong population, no eligible cerebrovascular or cognitive outcome, observational design without exercise training, review, editorial, protocol, or conference abstract only, insufficient intervention or outcome information, duplicate dataset or secondary report, and other reasons. Full-text exclusion reasons are reported in Supplementary Table S4.

### 2.6. Data Charting Process

A standardized data charting form was developed in accordance with scoping review guidance [16]. Extracted variables included study identification, design, participant characteristics, health-risk profile, recruitment setting, exercise modality and prescription, comparator condition, adherence, dropout, adverse events, cerebrovascular outcomes, cognitive outcomes, biological or mechanistic markers, timing of assessment, main findings, and implementation-relevant reporting features.

**Figure 3:**
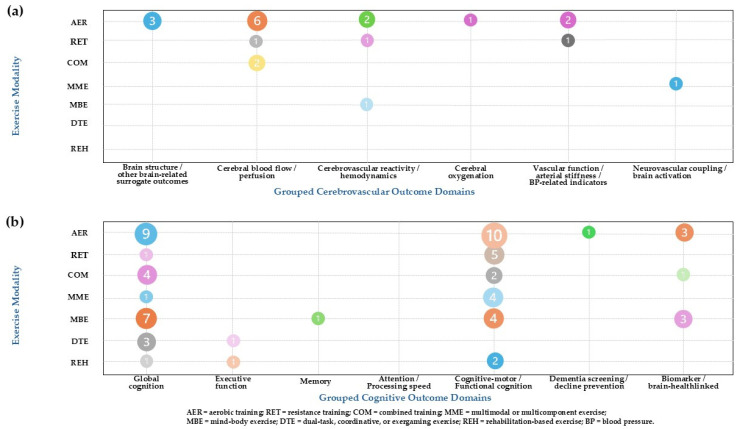
Evidence maps of exercise modalities by grouped cerebrovascular and cognitive outcome domains. (**a**) Panel A summarizes exercise modalities by grouped cerebrovascular or brain-related outcome domains. (**b**) Panel B summarizes exercise modalities by grouped cognitive outcome domains. The figure is structured as a modality × outcome matrix, with exercise modalities displayed against grouped outcome-domain cells. Bubble size and the number inside each bubble represent the number of included studies within each modality outcome cell, while bubble shading indicates relative evidence density. This visual synthesis was used to identify areas of evidence concentration, sparse evidence, evidence voids, underrepresented modality outcome combinations, and vascular cognitive integration gaps relevant to healthcare translation and community implementation. A separate cross-tabulation table is provided in the Section 3 to make the intersection between cerebrovascular and cognitive outcome assessment more explicit. AER = aerobic training; RET = resistance training; COM = combined training; MME = multimodal or multicomponent exercise; MBE = mind–body exercise; DTE = dual-task, coordinative, or exergaming exercise; REH = rehabilitation-based exercise.

Data charting was performed using the standardized form and checked against the predefined PCC criteria and operational coding rules. The form was refined iteratively when recurring study features or reporting gaps were identified. Uncertain extraction or coding decisions were resolved through discussion before final evidence-map coding. Original study-level outcome labels were retained for traceability and later grouped into broader cerebrovascular and cognitive domains for Figure 3 visualization. The data charting framework is summarized in Supplementary Table S5.

Exercise intervention characteristics were extracted according to the FITT-VP framework, including frequency, intensity, time, type, volume, and progression, and were supplemented by reporting elements relevant to intervention replication and healthcare delivery [18,19]. Additional variables included program duration, progression, supervision, delivery setting, adherence, dropout, adverse events, comparator condition, and feasibility-related information when reported. Because incomplete exercise-dose reporting limits replication and interpretation, FITT-VP elements were charted separately when available, with particular attention to whether studies reported intensity prescription and monitoring, weekly or total exercise volume, progression criteria, supervision, and adherence. Exercise modalities were categorized as aerobic training, resistance training, combined aerobic and resistance training, high-intensity interval training, multimodal or multicomponent exercise, mind–body exercise, dual-task exercise, coordinative or balance exercise, exergaming, rehabilitation-based exercise, or other structured exercise. Detailed exercise intervention characteristics are reported in Supplementary Table S6.

Cerebrovascular and brain-related outcomes were first extracted according to the terminology reported in the original studies and then organized by outcome domain and assessment method. Original outcome labels included cerebral blood flow, brain perfusion, cerebral blood velocity, middle cerebral artery velocity, cerebrovascular reactivity, cerebral oxygenation, neurovascular coupling, brain activation, endothelial or vascular function, arterial stiffness, vascular compliance, and blood pressure-related vascular indicators relevant to brain health. For evidence-map visualization, closely related outcomes were grouped into broader domains, including brain structure or other brain-related surrogate outcomes, cerebral blood flow or perfusion, cerebrovascular reactivity or hemodynamics, cerebral oxygenation, vascular function or arterial stiffness or blood pressure-related indicators, and neurovascular coupling or brain activation. Cerebrovascular reactivity was grouped with hemodynamic regulation because both categories reflect dynamic vascular responsiveness to physiological or task-related demands rather than static brain structure. Cerebral blood flow, cerebral perfusion, and cerebral blood velocity were kept conceptually distinct when possible, but were grouped into broader flow or perfusion-related domains for visualization when studies reported closely related indicators of cerebral circulation. These grouping decisions were made only for evidence-map visualization and did not replace the original outcome labels recorded during data charting. Grouped cerebrovascular or brain-related domains should therefore be interpreted as descriptive evidence-map categories rather than equivalent physiological constructs or interchangeable measurement endpoints. Reported assessment methods in the included studies included transcranial Doppler ultrasound, arterial spin labeling magnetic resonance imaging, functional magnetic resonance imaging, near-infrared spectroscopy, carbon dioxide challenge, breath-holding tasks, vascular function testing, and brain activation or structural imaging methods, when reported. Because these tools differ in the physiological constructs they capture, as well as in spatial and temporal resolution, sensitivity to regional or task-related change, participant burden, and ecological validity, assessment methods were charted together with outcome domains to distinguish measures of blood velocity, regional perfusion, oxygenation, vascular responsiveness, brain activation, and structural or vascular surrogate outcomes.

Cognitive outcomes were first extracted according to the terminology and assessment tools reported in the original studies and then classified into broader domains for evidence-map visualization. Original cognitive outcomes included global cognition, executive function, memory, attention, processing speed, working memory, inhibitory control, cognitive flexibility, verbal fluency, cognitive-motor performance, functional cognition, dementia-related screening outcomes, biomarker-linked cognitive outcomes, and other relevant cognitive measures. Assessment tools included the Mini-Mental State Examination, Montreal Cognitive Assessment, Trail Making Test, Stroop test, Digit Span, verbal fluency tasks, memory recall tests, and computerized cognitive batteries, when reported. For Figure 3, closely related cognitive outcomes were grouped into global cognition, executive function, memory, attention or processing speed, cognitive-motor or functional cognition, dementia-related screening or decline prevention, and biomarker-linked or brain-health-related cognitive outcomes. Working memory was grouped under executive function when the task primarily involved updating, attentional control, manipulation of information, or cognitive control processes. When studies reported global screening tools such as the MMSE or MoCA, these outcomes were grouped under global cognition or dementia-related screening rather than treated as domain-specific measures. Grouped cognitive domains were therefore used to reduce fragmentation in the evidence map, but they should not be interpreted as equivalent constructs across different neuropsychological tests. For example, tests grouped under executive function, memory, attention or processing speed, global cognition, or cognitive-motor performance may differ substantially in task demands, scoring procedures, psychometric properties, and sensitivity to exercise-induced change. Detailed cerebrovascular, brain-related, and cognitive outcome information is provided in Supplementary Table S7, and study-level grouped evidence-map coding is provided in Supplementary Table S9.

Outcome-integration coding was conducted to determine whether each study assessed cerebrovascular outcomes only, cognitive outcomes only, or both cerebrovascular and cognitive outcomes within the same intervention design. Studies reporting both domains were coded as especially relevant for healthcare translation because they allowed a more direct assessment of whether exercise-related vascular adaptation was examined alongside cognitive outcomes. This coding approach supported the identification of evidence gaps that may limit mechanism-informed exercise prescription in healthcare, rehabilitation, and community-based prevention settings.

### 2.7. Data Synthesis and Evidence Mapping

Findings were synthesized descriptively because this scoping review aimed to map the distribution, characteristics, and translational readiness of the evidence rather than estimate pooled intervention effects [15,16]. Descriptive summaries were used to report the distribution of studies by publication year, country, study design, population category, health-risk profile, exercise modality, intervention duration, originally reported outcome domain, assessment method, grouped outcome domain, and outcome-integration category. Because of heterogeneity in populations, exercise prescriptions, outcome measures, assessment methods, and study designs, no meta-analysis or pooled effect estimation was performed.

An evidence map was developed to visualize how exercise modalities were studied in relation to grouped cerebrovascular and cognitive outcome domains. The visual synthesis was structured as a modality × outcome matrix. Exercise modalities were placed on one axis, and grouped cerebrovascular, brain-related, or cognitive outcome domains were placed on the other axis. This matrix structure was used to show not only which exercise modalities had been studied, but also which outcome domains were paired with each modality. Evidence density was represented through bubble size, the numeric count within each bubble, and shading intensity, thereby allowing cells with concentrated, sparse, or absent evidence to be identified visually. Each included study was coded according to exercise modality, population category, original cerebrovascular or cognitive outcome label, grouped cerebrovascular or cognitive outcome domain, outcome-integration category, evidence density, methodological and reporting maturity, and exercise dose reporting quality. These coding categories were selected to identify not only areas of evidence concentration, but also gaps that may limit healthcare translation, including incomplete exercise dose reporting, limited adherence or safety information, limited feasibility reporting, and insufficient integration of vascular and cognitive outcomes within the same intervention design. The evidence map coding framework was developed to identify areas of evidence concentration, sparse evidence, evidence voids, underrepresented modality–outcome combinations, and gaps in vascular–cognitive outcome integration.

The evidence map was presented as a two-panel bubble matrix. Panel A summarized exercise modalities by grouped cerebrovascular and brain-related outcome domains, whereas Panel B summarized exercise modalities by grouped cognitive outcome domains. Closely related outcome labels were grouped before visualization to improve interpretability and reduce excessive fragmentation in the evidence map. Bubble size and the number inside each bubble represented the number of studies within each exercise modality by grouped outcome-domain cell. Bubble shading visually reinforced relative evidence density, with darker bubbles indicating cells with a larger number of studies. The final evidence map is presented in Figure 3, and the original study-level outcome labels, grouped domain assignments, and detailed evidence-map coding are provided in Supplementary Table S9.

The evidence map was used to identify areas of evidence concentration, underrepresented exercise modality by outcome-domain combinations, and the extent to which cerebrovascular and cognitive outcomes were assessed within the same intervention designs. For the purpose of gap mapping, cells with no studies were interpreted as evidence voids, cells with only one or very few studies were interpreted as sparse evidence areas, and cells dominated by cognitive outcomes without corresponding cerebrovascular assessment were interpreted as vascular cognitive integration gaps. This approach allowed the evidence map to function not only as a descriptive summary, but also as a visual tool for identifying modality-specific and outcome-specific limitations relevant to healthcare translation. Studies reporting both cerebrovascular and cognitive outcomes were identified through outcome-integration coding because they provide the most direct evidence for evaluating whether exercise-related vascular adaptations were examined alongside cognitive outcomes. This outcome-integration approach was used to determine where the current evidence base remains insufficient for mechanism-informed exercise prescription in healthcare, rehabilitation, and community-based dementia prevention settings.

### 2.8. Methodological Quality Mapping

Because the review aimed to map the evidence rather than determine comparative effectiveness, a formal risk-of-bias assessment was not used to exclude studies. Instead, selected methodological and reporting features were charted to contextualize evidence maturity and translational readiness [15,16]. These features included study design, randomization, comparator type, allocation procedures when reported, blinding of outcome assessment, attrition, adherence reporting, adverse event reporting, clarity of exercise prescription, use of objective intensity monitoring, completeness of outcome reporting, and feasibility-related information. This approach was intended to support evidence mapping and healthcare translation, and should not be interpreted as a formal risk-of-bias assessment, certainty-of-evidence judgment, or comparative effectiveness appraisal.

Methodological and reporting maturity were summarized descriptively and coded for evidence mapping purposes. Studies were categorized descriptively as having higher, moderate, or preliminary methodological and reporting maturity based on predefined evidence-map indicators, including study design, comparator condition, exercise prescription clarity, adherence reporting, adverse event reporting, and relevant outcome assessment. Higher methodological and reporting maturity generally reflected randomized controlled designs with an appropriate comparator, clearly described exercise prescription, adherence reporting, and relevant outcome assessment. Moderate methodological and reporting maturity reflected controlled or quasi-experimental designs with some methodological limitations, whereas preliminary methodological and reporting maturity reflected single-arm, pilot, feasibility, or small pre-post studies with limited control of bias. These categories were used to contextualize the evidence map and identify differences in evidence maturity across exercise modalities and grouped outcome domains. They should not be interpreted as formal risk-of-bias ratings or as evidence that one intervention modality has higher causal certainty than another.

Exercise dose reporting quality was also coded because incomplete intervention reporting limits replication, implementation, and translation into exercise prescription. Studies were categorized as having complete, partial, or limited FITT reporting based on whether frequency, intensity, time, type, progression, and supervision were clearly described. This methodological quality mapping supported the interpretation of the evidence map and the identification of future research priorities relevant to healthcare delivery, rehabilitation planning, and community-based dementia prevention. The operational definitions and coding rules for methodological and reporting maturity and exercise dose reporting quality are provided in Supplementary Table S8, and the study-level evidence-map coding is provided in Supplementary Table S9. The completed PRISMA-ScR checklist with section-based manuscript references is provided in Supplementary Table S10. It is placed after the search, screening, data-charting, intervention-characteristics, outcome-domain, and evidence-map coding supplements to preserve the sequence of methodological documentation across the Supplementary Files.

## 3. Results

### 3.1. Study Selection Results

The study selection process is summarized in Figure 2. A total of 40,113 records were identified through database searching, including 12,924 records from PubMed/MEDLINE and 27,189 records from Scopus. After export and reference management, 40,114 records were imported into the screening workflow. The one-record discrepancy between the database search count and the imported record count was identified during reference management and resolved during deduplication.

After the removal of 10,030 duplicate records, 30,084 records remained for further processing. Because the initial search strategy was intentionally broad and designed to maximize sensitivity across exercise, cerebrovascular, cognitive, aging, and healthcare-related terms, a predefined rule-based prescreening step was applied before title and abstract screening. This step was used to improve feasibility while maintaining alignment with the PCC framework and to remove only records that were clearly unrelated to structured exercise training, adult or aging-related populations, and cerebrovascular, vascular-risk, cognitive, dementia-related, or brain-health outcomes. Records with uncertain eligibility were retained for title and abstract screening rather than excluded during prescreening. After prescreening, 1210 records were retained for title and abstract screening.

During title and abstract screening, 1034 records were excluded because they did not meet the eligibility criteria. A total of 176 reports were sought for retrieval, of which 6 reports could not be retrieved. Therefore, 170 full-text reports were assessed for eligibility. Of these, 116 reports were excluded for predefined reasons, including no eligible cerebrovascular or cognitive outcome, no structured exercise training intervention, wrong population, observational design without exercise training, acute single-session exercise only, review, protocol, editorial, or conference abstract only, insufficient intervention or outcome information, or duplicate dataset or secondary report.

Finally, 54 studies met the eligibility criteria and were included in the descriptive synthesis and evidence map. Outcome-integration coding and grouped outcome-domain coding were conducted after study inclusion to support the evidence-map analysis.

### 3.2. General Characteristics of Included Studies

A total of 54 studies were included in this scoping review and evidence map [13,14,20,21,22,23,24,25,26,27,28,29,30,31,32,33,34,35,36,37,38,39,40,41,42,43,44,45,46,47,48,49,50,51,52,53,54,55,56,57,58,59,60,61,62,63,64,65,66,67,68,69,70,71]. The distribution of included studies by outcome-integration category, exercise modality, population risk profile, and grouped outcome domain is summarized in Table 2. Detailed study-level characteristics, original outcome labels, and grouped evidence-map coding are provided in Supplementary Table S9. Study designs varied across the 54 included studies. Based on the reported study design labels and study-level extraction, 27 studies were described as randomized controlled trials, randomized clinical trials, waitlist randomized trials, group-randomized trials, or other randomized trials without an explicit pilot label. Four additional studies were explicitly described as pilot randomized or pilot three-arm randomized trials. Five studies were described as controlled, comparative, simultaneous, combined nutrition-exercise, or non-randomized clinical or intervention studies. One study was described as a community-based pilot study. The remaining 17 reports were intervention reports, trial-related analyses, rehabilitation-based studies, or studies in which the randomization or comparator structure was not clearly specified in the extracted design label. These categories were used descriptively to characterize the evidence base and should not be interpreted as formal risk-of-bias ratings.

The included studies were grouped into three outcome-integration categories. Thirty-eight studies reported cognitive outcomes only [13,14,26,27,37,38,39,40,41,42,43,44,45,46,47,48,49,50,51,52,53,54,55,56,57,58,59,60,62,63,64,65,66,67,68,69,70,71], nine studies reported cerebrovascular or vascular outcomes relevant to brain health without direct cognitive testing [20,21,22,23,29,30,32,35,36], and seven studies assessed both cerebrovascular and cognitive outcomes within the same intervention design [24,25,28,31,33,34,61]. Thus, cognitive outcomes represented the largest evidence category, whereas fewer studies directly examined cerebrovascular outcomes or integrated vascular and cognitive endpoints.

Exercise modalities were diverse across the included studies. Aerobic training was the most frequently represented modality, accounting for 17 studies [20,21,22,23,24,25,26,27,28,29,33,34,35,36,37,38,39], followed by multimodal or multicomponent exercise with 12 studies [13,31,32,40,41,42,43,45,57,65,66,67]. Combined aerobic and resistance training or combined physical-cognitive exercise accounted for six studies [44,48,49,59,60,64], and dual-task, coordinative, or exergaming exercise also accounted for six studies [30,46,47,61,62,63]. Mind–body exercise accounted for five studies [14,50,51,52,53], whereas resistance training and rehabilitation-based exercise each accounted for four studies [54,55,56,58,68,69,70,71].

Overall, the included studies covered a broad range of intervention approaches, but the distribution of evidence was uneven. Cognitive outcomes were assessed across nearly all exercise modalities, whereas direct cerebrovascular or brain-related outcomes were concentrated mainly in aerobic or aerobic-based interventions. Integrated vascular-cognitive designs remained uncommon, limiting the current basis for mechanism-informed exercise prescription across different population risk profiles.

### 3.3. Population Characteristics and Health-Risk Profiles

The included studies enrolled adult and older adult populations with varying levels of cognitive, vascular, metabolic, and functional risk [13,14,20,21,22,23,24,25,26,27,28,29,30,31,32,33,34,35,36,37,38,39,40,41,42,43,44,45,46,47,48,49,50,51,52,53,54,55,56,57,58,59,60,61,62,63,64,65,66,67,68,69,70,71]. The most frequently represented population category was mild cognitive impairment or mild neurocognitive disorder [13,14,20,21,22,23,24,28,29,30,31,32,37,38,39,40,41,42,43,44,46,47,48,50,51,53,56,57,58,59,60,62,63,64,65,66,67]. Studies in this category examined a range of exercise approaches in relation to cognitive function, cerebral blood flow or perfusion, brain structure, and other brain-health-related outcomes. This concentration indicates that most available intervention evidence has been generated in populations with already identifiable cognitive vulnerability, rather than in earlier healthcare prevention stages.

Healthy older adults and sedentary or physically inactive adults were also represented [25,26,27,33,34,36,45]. These studies addressed exercise training as a preventive or health-promoting strategy for maintaining cerebrovascular or cognitive function during aging. In these populations, aerobic training was commonly used to examine cerebrovascular or brain-related outcomes, including cerebral blood flow or perfusion, cerebrovascular reactivity or hemodynamics, cerebral oxygenation, and related vascular indicators [25,33,34,36], whereas other studies assessed cognitive outcomes after structured aerobic, multimodal, or mind–body exercise programs [26,27,45]. These studies are relevant to community-based prevention and healthy aging programs, but their outcome selection was not always sufficient to determine whether cerebrovascular adaptation and cognitive response occurred together.

Several studies included adults with cardiometabolic or vascular risk factors, including hypertension, obesity, type 2 diabetes, cardiovascular disease, heart failure, or resistant hypertension [25,35,51,69,70,71]. These populations are highly relevant to healthcare translation because vascular and metabolic risk factors are modifiable contributors to cognitive decline. However, studies in these groups were fewer and were mainly represented by aerobic training, exercise-based lifestyle interventions, and rehabilitation-based programs when eligible cognitive, cerebrovascular, vascular, or brain-related outcomes were reported [68,69,70,71]. This indicates that adults with early vascular-cognitive risk remain underrepresented relative to their importance for dementia prevention and clinical risk management.

A smaller number of studies focused on subjective cognitive decline, cognitive frailty, frailty-related risk, or memory complaints [49,52,54,55,61,68]. Across these studies, interventions included combined training, resistance training, mind–body exercise, dual-task or exergaming approaches, or home-based physical activity programs targeting physical and cognitive function. These populations are important for healthcare and rehabilitation planning because they may require feasible, scalable, and functionally oriented exercise prescriptions. However, the limited number of studies and heterogeneous outcome measures make it difficult to determine which intervention models are most appropriate for different risk profiles.

Overall, the included populations were concentrated in older adults and cognitively at-risk groups, with fewer studies directly targeting middle-aged adults, adults with clearly defined early vascular-cognitive risk profiles, or participants recruited from routine healthcare, rehabilitation, or community prevention settings. This population distribution limits the current readiness of the evidence base for precision healthcare prescription because exercise recommendations may need to differ according to cognitive status, vascular risk burden, functional capacity, supervision needs, and care setting.

### 3.4. Exercise Intervention Characteristics

The included studies examined a diverse range of structured exercise training approaches. Aerobic training was the most frequently represented modality, accounting for 17 studies [20,21,22,23,24,25,26,27,28,29,33,34,35,36,37,38,39]. These interventions included walking, treadmill exercise, cycling, aerobic dance, aquatic treadmill exercise, and other endurance-based protocols. Aerobic training was also the most common modality among studies assessing cerebrovascular or brain-related outcomes, including cerebral blood flow or perfusion, cerebrovascular reactivity or hemodynamics, cerebral oxygenation, vascular function, arterial stiffness, blood pressure-related indicators, and related vascular-risk outcomes [20,21,22,23,24,25,28,29,33,34,35,36]. This pattern suggests that aerobic training currently provides the most developed intervention category for linking exercise prescription with cerebrovascular assessment, although the extent to which these vascular outcomes were paired with cognitive outcomes varied across studies.

Multimodal or multicomponent exercise was the second most common intervention category, accounting for 12 studies [13,31,32,40,41,42,43,45,57,65,66,67]. These programs typically combine aerobic exercise, resistance exercise, balance training, flexibility, functional training, coordination, and cognitive-motor tasks. Combined aerobic and resistance training or combined physical-cognitive exercise accounted for six studies [44,48,49,59,60,64]. Together, these intervention categories were mainly used in older adults, individuals with mild cognitive impairment, or populations with functional risk, and primarily assessed cognitive, functional-cognitive, or mobility-related outcomes. These approaches are highly relevant to healthcare and rehabilitation settings because they resemble real-world exercise prescriptions for older adults, but many studies did not include direct cerebrovascular assessment or detailed implementation-related reporting.

Dual-task, coordinative, or exergaming interventions accounted for six studies [30,46,47,61,62,63]. These interventions emphasized cognitive-motor integration through dual-task exercise, virtual reality-based exercise, exergame balance training, cognitive-motor training, or coordinative movement tasks. Mind–body exercise accounted for five studies and included Tai Chi, yoga, or related integrative approaches [14,50,51,52,53]. These modalities may be clinically attractive because they are often adaptable for older adults, community programs, and individuals with functional limitations. However, they were mainly represented in cognition-focused studies, and only selected studies included cerebral blood flow or perfusion, neurovascular coupling or brain activation, brain structure or other brain-related surrogate outcomes, biomarker-linked cognitive outcomes, or immunological markers [30,46,50,51,52,53,61]. Therefore, their potential cerebrovascular mechanisms remain less clearly mapped than those of aerobic training.

Resistance training accounted for four studies [54,55,56,58]. These interventions generally involved progressive strength training or resistance-based exercise in older adults, individuals with mild cognitive impairment, or cognitively frail populations, and mainly assessed cognitive outcomes, frailty-related outcomes, physical performance, strength gains, or brain structure-related measures. Rehabilitation-based exercise also accounted for 4 studies [68,69,70,71] and included cardiac rehabilitation or exercise-based lifestyle interventions in adults with cardiovascular or vascular-risk conditions. These studies are particularly relevant to healthcare translation because they involve clinical or rehabilitation contexts, but they remain a relatively small evidence cluster within the overall literature.

Overall, aerobic training and multimodal exercise represented the largest intervention categories, whereas resistance training, mind–body exercise, dual-task or exergaming interventions, and rehabilitation-based exercise were smaller evidence clusters. Across modalities, exercise prescription reporting varied in intensity, progression, supervision, adherence, adverse events, and feasibility. More consistent reporting is needed before modality-specific exercise prescriptions can be confidently translated into clinical, rehabilitation, or community prevention settings.

Clinical translation metrics were also inconsistently reported. Based on information extractable from the full texts and accessible study reports, adherence or attendance information was reported or operationally defined in at least 26 study reports, dropout, attrition, or completion information was reported in at least 29 study reports, and adverse events or safety-related outcomes were explicitly reported in at least 15 study reports. Reporting formats varied across studies, including attendance percentages, compliance with prescribed sessions, training logs, intervention completion rates, attrition counts, reasons for withdrawal, and qualitative safety statements. This variability limited direct comparison of feasibility and safety across exercise modalities and population risk profiles.

The methodological and reporting maturity categories further indicated uneven translational readiness across the evidence base. Based on the predefined descriptive evidence-map coding framework, 26 study reports were categorized as having higher methodological and reporting maturity, 22 as having moderate methodological and reporting maturity, and 6 as having preliminary methodological and reporting maturity. Based on the predefined FITT reporting-quality coding, 26 study reports were categorized as having complete FITT reporting, 24 as having partial FITT reporting, and 4 as having limited FITT reporting. Thus, although many studies used randomized or controlled designs, incomplete exercise-dose reporting remained common. This pattern limits replication, comparison of intervention dose, interpretation of dose–response patterns, and translation into healthcare-oriented exercise prescription. These descriptive categories should be interpreted with caution because they were not formal risk-of-bias ratings or certainty-of-evidence judgments. They identify patterns in study design, reporting completeness, and translational readiness, but they do not establish internal validity, causal certainty, or comparative effectiveness across exercise modalities. Therefore, findings related to evidence maturity should be interpreted as evidence-map indicators rather than as conclusions about intervention efficacy or risk of bias.

### 3.5. Cerebrovascular Outcome Domains

Among the 54 included studies, 16 reported cerebrovascular, vascular, or brain-related outcomes relevant to brain health [20,21,22,23,24,25,28,29,30,31,32,33,34,35,36,61]. Of these, 9 reported cerebrovascular or vascular outcomes without direct cognitive testing [20,21,22,23,29,30,32,35,36], whereas 7 assessed both cerebrovascular and cognitive outcomes within the same intervention design [24,25,28,31,33,34,61]. This distribution indicates that direct cerebrovascular or vascular assessment was included in a minority of exercise intervention studies, and that integrated vascular-cognitive assessment was even less common.

For evidence-map visualization, originally reported cerebrovascular, vascular, and brain-related outcomes were grouped into broader domains. Cerebral blood flow or perfusion was the most frequently represented domain [20,22,23,24,30,31,32,33,36]. Other domains included cerebrovascular reactivity or hemodynamics, cerebral oxygenation, vascular function, arterial stiffness, blood pressure-related indicators, neurovascular coupling or brain activation, and brain structure or other brain-related surrogate outcomes [20,21,25,28,29,34,35,61]. Assessment methods varied across magnetic resonance imaging-based perfusion measures, transcranial Doppler ultrasound, near-infrared spectroscopy, vascular stiffness assessment, hemodynamic testing, and brain activation or structural imaging. This methodological diversity supports mechanistic breadth but limits direct comparability across studies.

Aerobic training was the dominant modality among studies assessing cerebrovascular, vascular, or brain-related outcomes [20,21,22,23,24,25,28,29,33,34,35,36]. Of the 17 aerobic-focused studies, 12 reported cerebrovascular, vascular, or brain-related outcomes, including 7 cerebrovascular-only studies [20,21,22,23,29,35,36] and 5 studies that also reported cognitive outcomes [24,25,28,33,34]. This indicates that aerobic training currently represents the most developed evidence cluster for linking structured exercise training with cerebrovascular assessment. However, even within aerobic-focused studies, not all investigations paired vascular assessment with cognitive testing, limiting the ability to determine whether cerebrovascular changes were accompanied by cognitive responses in the same participants.

Non-aerobic modalities were less frequently represented in direct cerebrovascular or brain-related assessment. Dual-task or coordinative exercise accounted for two studies with cerebral blood flow, brain activation, or related brain-health outcomes [30,61], and multimodal or multicomponent exercise accounted for two studies with cerebral blood flow or perfusion-related outcomes [31,32]. Resistance training, mind–body exercise, combined training, and rehabilitation-based exercise were not represented in the cerebrovascular-only or integrated cerebrovascular-cognitive categories in the current outcome-integration coding. This gap is important from a healthcare perspective because these modalities are commonly used or highly adaptable in older adults, rehabilitation programs, community exercise settings, and individuals with functional limitations.

Overall, cerebrovascular, vascular, and brain-related outcomes were concentrated in aerobic or aerobic-based interventions, particularly in adults with mild cognitive impairment, older adults, sedentary or cardiometabolic-risk populations, and postmenopausal women. Several clinically feasible modalities, including resistance, mind–body, dual-task, and rehabilitation-based exercise, were rarely paired with direct cerebrovascular assessment. This imbalance limits interpretation of which modalities can produce vascular adaptations relevant to cognitive health.

### 3.6. Cognitive Outcome Domains

Cognitive outcomes were more frequently assessed than cerebrovascular outcomes. Among the 54 included studies, 45 reported at least one cognitive outcome [13,14,24,25,26,27,28,31,33,34,37,38,39,40,41,42,43,44,45,46,47,48,49,50,51,52,53,54,55,56,57,58,59,60,61,62,63,64,65,66,67,68,69,70,71]. This included 38 studies reporting cognitive outcomes only [13,14,26,27,37,38,39,40,41,42,43,44,45,46,47,48,49,50,51,52,53,54,55,56,57,58,59,60,62,63,64,65,66,67,68,69,70,71] and 7 studies reporting both cognitive and cerebrovascular outcomes [24,25,28,31,33,34,61]. Cognitive outcomes were assessed in individuals with mild cognitive impairment, healthy older adults, adults with subjective cognitive decline or memory complaints, cognitively frail older adults, and adults with cardiometabolic or vascular risk. This distribution indicates that cognitive testing has become a common endpoint in exercise intervention studies targeting aging and cognitive-risk populations.

For evidence-map visualization, originally reported cognitive outcomes were grouped into broader cognitive domains to improve interpretability and reduce excessive fragmentation. The grouped cognitive domains included global cognition, executive function, memory, attention or processing speed, cognitive-motor or functional cognition, dementia-related screening or decline prevention, and biomarker-linked or brain-health-related cognitive outcomes. Several studies used global cognitive screening measures or composite cognitive outcomes, whereas others assessed domain-specific outcomes such as executive function, memory, attention, processing speed, or cognitive-motor performance. Based on the study-level outcome coding, 45 studies reported at least one cognitive outcome. Of these, 42 studies included global cognition or broad cognitive screening/composite outcomes, whereas 11 studies included at least one domain-specific cognitive domain, such as executive function, memory, or cognitive-motor or functional cognition. Ten studies included both global cognition and at least one domain-specific cognitive domain, while 34 studies primarily reported global or broad cognitive outcomes without a clearly coded domain-specific cognitive outcome. This distribution suggests that much of the cognitive evidence relied on global or broad cognitive assessment rather than domain-specific neuropsychological testing. Although global screening tools such as the MMSE and MoCA are useful for clinical characterization and dementia-related screening, they may be less sensitive to subtle exercise-induced changes in executive function, attention, processing speed, working memory, memory, or cognitive-motor performance. Studies involving dual-task, exergaming, functional task, or multimodal interventions frequently included cognitive-motor or functional cognition outcomes [41,42,46,47,61,62,63,64,65,66]. This diversity reflects the broad relevance of cognitive outcomes to healthcare, rehabilitation, and community-based prevention, but it also limits comparability across studies when different tools, domains, labels, and composite measures are used.

Compared with cerebrovascular outcomes, cognitive outcomes were distributed across a wider range of exercise modalities. Cognitive-only studies included multimodal or multicomponent exercise, combined exercise, mind–body exercise, resistance training, dual-task or exergaming interventions, rehabilitation-based exercise, and aerobic exercise. Multimodal or multicomponent exercise accounted for 10 cognitive-only studies [13,40,41,42,43,45,57,65,66,67], followed by combined training with 6 studies [44,48,49,59,60,64], mind–body exercise with 5 studies [14,50,51,52,53], and resistance training, dual-task or exergaming exercise, and rehabilitation-based exercise with 4 studies each [46,47,54,55,56,58,62,63,68,69,70,71]. Aerobic-focused exercise accounted for five cognitive-only studies [26,27,37,38,39]. These findings show that cognition-focused exercise research is broader than cerebrovascular-focused exercise research in terms of intervention modality.

Overall, cognitive outcomes were represented across nearly all exercise modalities, whereas direct cerebrovascular or vascular assessments were less frequently included in cognition-focused studies. Cognitive findings therefore cannot be interpreted as evidence of exercise-induced cerebrovascular adaptation unless vascular outcomes are assessed within the same intervention design. Future studies should integrate domain-specific cognitive testing with cerebrovascular assessment, exercise dose reporting, adherence monitoring, safety reporting, and feasibility evaluation.

### 3.7. Evidence Map of Exercise Modalities and Outcome Domains

Figure 3 visualizes how the 54 included studies were distributed across exercise modalities and grouped cerebrovascular and cognitive outcome domains. The coding framework is summarized in Table 3, with operational definitions and decision rules provided in Supplementary Table S8. Original study-level outcome labels, grouping decisions, and detailed coding data are provided in Supplementary Table S9.

Figure 3a shows that cerebrovascular outcomes were concentrated in fewer modalities and outcome categories than cognitive outcomes. Aerobic training formed the largest cerebrovascular cluster, particularly for cerebral blood flow or perfusion, cerebrovascular reactivity or hemodynamics, cerebral oxygenation, arterial stiffness or blood pressure-related indicators, and related vascular or brain-health outcomes [20,21,22,23,24,25,28,29,33,34,35,36]. Non-aerobic modalities contributed fewer cerebrovascular or brain-related outcome cells, mainly involving multimodal exercise and dual-task or coordinative exercise [30,31,32,61].

Figure 3b shows that cognitive outcomes were distributed across a broader range of exercise modalities [13,14,24,25,26,27,28,31,33,34,37,38,39,40,41,42,43,44,45,46,47,48,49,50,51,52,53,54,55,56,57,58,59,60,61,62,63,64,65,66,67,68,69,70,71]. Outcome labels varied from broad cognitive function measures to domain-specific outcomes such as memory, executive function, cognitive-motor function, frailty-related cognition, biomarker-linked cognition, and health benefit-related cognition. This pattern indicates wider modality coverage but less standardized cognitive outcome reporting.

Taken together, Figure 3 indicates a major outcome-integration gap. Cerebrovascular outcomes were concentrated in fewer modalities, whereas cognitive outcomes were more widely distributed but less consistently labeled. Few studies combined exercise dose reporting, cerebrovascular assessment, cognitive testing, adherence monitoring, safety reporting, feasibility information, and mechanistic biomarkers within the same intervention design. Future trials should integrate vascular and cognitive endpoints while reporting implementation features needed for rehabilitation, community-based prevention, and dementia risk management. To complement the modality-specific evidence maps in Figure 3, a cross-tabulation of outcome-domain intersection was added to show the degree of overlap between cerebrovascular and cognitive assessment across the 54 included studies. Only 7 studies assessed both cerebrovascular and cognitive outcomes within the same intervention design, whereas 38 studies assessed cognitive outcomes only and 9 assessed cerebrovascular or vascular outcomes only. This cross-domain distribution highlights the central vascular-cognitive integration gap in the current evidence base. Detailed coding definitions and study-level evidence-map characteristics are provided in Supplementary Tables S8 and S9 to preserve traceability while keeping the main text focused on the principal evidence patterns (Table 4).

### 3.8. Methodological Characteristics and Reporting Features

Across the 54 included studies, methodological characteristics varied by study design, exercise modality, population risk profile, and outcome domain [13,14,20,21,22,23,24,25,26,27,28,29,30,31,32,33,34,35,36,37,38,39,40,41,42,43,44,45,46,47,48,49,50,51,52,53,54,55,56,57,58,59,60,61,62,63,64,65,66,67,68,69,70,71]. The evidence base included randomized controlled trials, pilot randomized trials, quasi-experimental studies, controlled pre-post interventions, single-arm training studies, and rehabilitation-based interventions. Randomized and controlled designs were more common among cognition-focused trials, whereas several cerebrovascular-focused studies used smaller mechanistic or pilot designs, reflecting the technical demands of cerebral blood flow, cerebrovascular reactivity, cerebral oxygenation, and brain activation assessment. This pattern suggests that mechanistic cerebrovascular studies and pragmatic cognition-focused trials have developed along partly separate pathways.

Exercise prescription reporting varied across studies. Many studies described the general exercise modality and intervention duration, but details regarding intensity prescription, progression, supervision, adherence, and adverse events were not consistently reported. Aerobic training studies more frequently reported exercise intensity using heart rate, workload, or cardiorespiratory fitness-related targets. Resistance training studies often reported training type and progression, whereas multimodal, dual-task, exergaming, mind–body, and rehabilitation-based interventions were generally more complex and made it more difficult to distinguish the contribution of each exercise component. This variability limits the ability to compare exercise dose across studies and reduces the usefulness of the evidence for modality-specific healthcare prescriptions.

Adherence and supervision were also variable. Supervised laboratory or clinical interventions provided clearer control over exercise dose, whereas home-based or community-based programs offered greater ecological relevance but often depended on self-monitoring, caregiver support, remote supervision, or adherence tracking. Rehabilitation-based and exercise-based lifestyle interventions were distinguished from exercise-only trials because cognitive outcomes may also have been influenced by broader clinical care, diet, education, risk-factor management, or behavioral support. These features affect whether an intervention can be transferred to routine community, outpatient, or long-term care settings.

Overall, methodological quality mapping showed that the field includes both clinically oriented intervention trials and smaller mechanistic studies. Studies with randomized controlled designs, appropriate comparators, clear exercise prescriptions, adherence reporting, and relevant outcome assessment were generally categorized as having higher methodological and reporting maturity, whereas pilot, feasibility, single-arm, or small pre-post studies were categorized as preliminary evidence. Key reporting gaps included incomplete FITT reporting, limited follow-up, inconsistent adverse event reporting, heterogeneous cognitive test batteries, limited feasibility reporting, and few integrated vascular-cognitive designs. These gaps should be addressed in future trials to improve replication, interpretation, and translation into rehabilitation, community-based prevention, and dementia risk management.

## 4. Discussion

### 4.1. Principal Findings

This scoping review shows that the exercise and brain health literature has developed along two partly separate tracks: one has evaluated exercise training as a strategy for improving cognition, whereas the other has examined cerebrovascular or vascular adaptation as a physiological response to training. Across the 54 included studies, only 7 assessed both domains within the same intervention design, indicating that the central limitation is not simply underreporting of cerebrovascular outcomes, but insufficient integration of vascular and cognitive endpoints in the same participants [4,5,72,73,74,75,76]. In this review, precision exercise prescription refers to the clinically informed tailoring of exercise modality, dose, progression, supervision, and monitoring according to baseline cognitive status, vascular or cardiometabolic risk burden, functional capacity, adherence needs, safety profile, and the intended cerebrovascular or cognitive outcome target. It does not refer to genotype-based personalization or to a currently established biomarker-driven exercise algorithm. In this review, precision exercise prescription refers to the clinically informed tailoring of exercise modality, dose, progression, supervision, and monitoring according to baseline cognitive status, vascular or cardiometabolic risk burden, functional capacity, adherence needs, safety profile, and the intended cerebrovascular or cognitive outcome target. It does not refer to genotype-based personalization or to a currently established biomarker-driven exercise algorithm.

The grouped evidence map in Figure 3 further clarifies this imbalance. Cerebrovascular outcomes were concentrated in fewer modality-outcome cells, especially within aerobic training and cerebral blood flow or perfusion-related domains. In contrast, cognitive outcomes were distributed across a broader range of exercise modalities, including aerobic, resistance, combined, multimodal, mind–body, dual-task or exergaming, and rehabilitation-based interventions. This contrast shows that cognition-focused exercise research is broader in intervention scope, whereas cerebrovascular-focused research remains more concentrated in specific modalities and assessment domains.

Aerobic training emerged as the most developed platform for studying exercise-related cerebrovascular adaptation. This pattern is biologically plausible because aerobic exercise provides a sustained cardiovascular stimulus that can be prescribed, monitored, and progressed using relatively objective indicators such as heart rate, workload, or cardiorespiratory fitness-related targets. The greater representation of aerobic training in cerebrovascular studies should therefore be interpreted not only as a finding about exercise modality, but also as a reflection of methodological feasibility in vascular and hemodynamic research [20,21,22,23,24,25,28,29,33,34,35,36]. However, this concentration also highlights an important translational gap. Several clinically relevant non-aerobic modalities, including resistance training, mind–body exercise, dual-task or exergaming interventions, and rehabilitation-based exercise, remain less frequently examined with direct cerebrovascular measures despite their potential feasibility in older adults, rehabilitation programs, and community-based prevention settings.

Cognitive outcomes were distributed across a broader set of exercise modalities, suggesting that exercise may influence cognition through multiple pathways, including cardiorespiratory adaptation, muscular strengthening, cognitive-motor challenge, attentional regulation, functional training, social engagement, and vascular-risk management [5,77,78,79,80,81,82,83,84]. However, because most cognition-focused studies did not include direct cerebrovascular assessment, these findings cannot establish that cognitive changes were mediated by vascular or cerebrovascular mechanisms.

Taken together, the evidence map identifies a central limitation in the current literature. Exercise training is frequently proposed to support cognitive health through vascular and cerebrovascular mechanisms, yet the vascular and cognitive components of this pathway are often evaluated separately [6,8,9,11,72,73,76]. This separation between mechanistic vascular assessment and clinical cognitive evaluation limits the ability to move from general exercise recommendations toward mechanism-informed healthcare prescription. Future trials should therefore integrate exercise dose reporting, cerebrovascular assessment, domain-specific cognitive testing, adherence monitoring, safety reporting, feasibility evaluation, and relevant physiological or behavioral mediators to support more precise exercise prescriptions for brain health, rehabilitation planning, and dementia risk mitigation [18,19,85].

### 4.2. Aerobic Training as the Dominant Cerebrovascular Evidence Cluster

Aerobic training formed the most developed evidence cluster for cerebrovascular and vascular outcomes relevant to brain health. In the grouped evidence map, aerobic-focused studies contributed the largest share of direct cerebrovascular and brain-related assessments, particularly within cerebral blood flow or perfusion, cerebrovascular reactivity or hemodynamics, cerebral oxygenation, vascular function or arterial stiffness or blood pressure-related indicators, and related brain-health surrogate outcomes [20,21,22,23,24,25,28,29,33,34,35,36]. This concentration suggests that aerobic training has become the primary model for examining exercise-related cerebrovascular adaptation, particularly in older adults and adults with vascular or cognitive risk.

The prominence of aerobic training also reflects its methodological suitability for cerebrovascular research. Aerobic interventions can usually be prescribed and monitored using heart rate, oxygen uptake, workload, walking speed, cycling power, or perceived exertion, allowing clearer dose quantification than many complex multicomponent interventions. This makes aerobic exercise well-suited for studies of cerebral blood flow, brain perfusion, cerebrovascular reactivity, arterial stiffness, cerebral oxygenation, and related hemodynamic outcomes [74,86,87]. Its relative ease of prescription and monitoring also supports use in outpatient, rehabilitation, and community-based prevention settings.

Mechanistically, aerobic training may support cerebrovascular health through both systemic and brain-specific pathways. Improvements in cardiorespiratory fitness may enhance central hemodynamic efficiency and oxygen delivery, whereas reductions in blood pressure and arterial stiffness may lower pulsatile stress transmitted to the cerebral microcirculation [74,75,76]. Enhanced endothelial function and nitric oxide bioavailability may further improve vascular reactivity, including cerebrovascular responses to metabolic or carbon dioxide-related stimuli [6,9,74,75]. Aerobic training may also influence angiogenesis, neurotrophic signaling, inflammatory regulation, oxidative stress balance, and metabolic health, all of which may contribute indirectly to cognitive resilience [3,4,5,84]. These mechanisms provide a plausible rationale for the emphasis on aerobic exercise in cerebrovascular exercise research.

However, the presence of an aerobic evidence cluster should not be interpreted as uniform evidence for a single cerebrovascular effect. The included studies used diverse assessment methods, including magnetic resonance imaging-based perfusion measures, transcranial Doppler ultrasound, near-infrared spectroscopy, vascular stiffness assessment, and hemodynamic or brain activation measures [20,21,22,23,24,25,28,29,30,31,32,33,34,35,36,61]. These methods capture different physiological constructs, such as resting perfusion, regional flow, blood velocity, vascular responsiveness, task-related oxygenation, and systemic vascular load [73,86,87]. A related limitation is the lack of standardized cerebrovascular measurement protocols across studies. ASL MRI-, SPECT-, TCD-, NIRS-, fMRI-based activation measures, and peripheral vascular assessments differ not only in technical implementation but also in the physiological constructs they represent. ASL MRI and SPECT provide information about regional perfusion, TCD primarily captures blood velocity in large cerebral arteries, NIRS reflects cortical oxygenation within a limited tissue depth, and task-based fMRI or NIRS measures hemodynamic activation rather than resting vascular status. As a result, findings labeled broadly as cerebrovascular outcomes may reflect different biological processes, including perfusion, velocity, oxygen extraction, vascular reactivity, neural activation, or systemic vascular load. This measurement heterogeneity limits direct comparison across studies and makes it difficult to determine whether inconsistent findings reflect true differences in exercise-induced vascular adaptation, differences in population responsiveness, or differences in measurement sensitivity and construct validity. Future studies should therefore specify the vascular construct being assessed, standardize acquisition and analytic procedures where possible, and align cerebrovascular measures with cognitive outcomes measured in the same participants.

These methodological differences also have implications for reliability, sensitivity, and ecological validity. For example, transcranial Doppler ultrasound provides high temporal resolution for blood velocity but does not directly quantify regional tissue perfusion; arterial spin labeling MRI provides regional perfusion information but is more resource-intensive and less ecologically flexible; and near-infrared spectroscopy can capture oxygenation changes during task-related or exercise-related conditions but may be affected by probe placement, extracerebral signal contamination, and limited regional coverage. Therefore, differences across TCD, NIRS, MRI-based perfusion methods, SPECT, and related tools may influence whether cerebrovascular change is detected, how that change is interpreted, and whether findings can be compared across intervention settings.

Similar caution applies to the grouped outcome domains used in the evidence map. These domains were created to improve readability and to visualize evidence distribution across exercise modalities, but they do not imply that all outcomes within a category measure the same construct. For example, cerebral blood flow, perfusion, blood velocity, cerebrovascular reactivity, oxygenation, and neurovascular coupling reflect related but distinct aspects of cerebrovascular physiology. Likewise, cognitive domains may include heterogeneous neuropsychological tests with different task demands, scoring systems, and sensitivity to intervention effects. Therefore, evidence-map cells should be interpreted as indicators of where outcomes have been studied, rather than as evidence that grouped measures are mechanistically or psychometrically equivalent.

They also differ in reliability, sensitivity, and ecological validity. Transcranial Doppler ultrasound provides high temporal resolution for blood velocity responses but does not directly quantify regional tissue perfusion. MRI-based perfusion methods, such as arterial spin labeling, provide spatially resolved estimates of cerebral perfusion but are more resource-intensive and less feasible for frequent repeated assessments. Near-infrared spectroscopy is more portable and suitable for task-based or exercise-adjacent assessment, but it reflects cortical oxygenation within a limited measurement depth and may be influenced by extracerebral signals. These methodological differences mean that apparently similar cerebrovascular outcomes may not be directly comparable across studies. Studies also differed in population risk profile, intervention duration, intensity prescription, supervision, adherence reporting, and comparator conditions. Therefore, aerobic training should be viewed as the most mature platform for studying exercise-related cerebrovascular adaptation, rather than as evidence that a uniform vascular response occurs across all populations and outcomes.

Although aerobic training currently has the strongest cerebrovascular evidence base among the modalities mapped in this review, the evidence remains limited by heterogeneity in dose reporting, outcome selection, follow-up duration, and integration with cognitive testing. Future aerobic exercise trials should combine standardized cerebrovascular outcomes, domain-specific cognitive testing, adherence monitoring, safety reporting, feasibility evaluation, and clearly defined dose progression before aerobic training can be translated into mechanism-informed prescriptions for adults at risk of cognitive decline [72,73].

### 4.3. Cognitive Outcomes Are Broadly Studied but Mechanistically Underlinked

The cognitive evidence base was broader than the cerebrovascular evidence base, but it was also more conceptually diffuse. Cognitive outcomes were grouped into seven domains, including global cognition, executive function, memory, attention or processing speed, cognitive-motor or functional cognition, dementia-related screening or decline prevention, and biomarker-linked or brain-health-related cognitive outcomes. These outcomes appeared across nearly all exercise modalities [13,14,24,25,26,27,28,31,33,34,37,38,39,40,41,42,43,44,45,46,47,48,49,50,51,52,53,54,55,56,57,58,59,60,61,62,63,64,65,66,67,68,69,70,71], indicating that exercise is widely studied as a candidate strategy for cognitive health. However, the diversity of tests, labels, and outcome domains makes it difficult to determine whether different modalities influence cognition through shared pathways or modality-specific mechanisms, especially in older adults and populations with cognitive, frailty-related, vascular, or cardiometabolic risk [3,5,83,84,88].

Different exercise modalities may support cognition through partially distinct pathways. Multimodal and multicomponent exercise may target cardiorespiratory fitness, strength, balance, mobility, coordination, functional independence, and cognitive-motor integration [13,31,32,40,41,42,43,45,57,65,66,67,78,79]. Dual-task, coordinative, and exergaming interventions may engage executive control, attention, task-switching, balance, and motor-cognitive coordination [30,46,47,61,62,63,80,81]. Mind–body exercise may support cognition through attentional regulation, postural control, autonomic modulation, stress reduction, breathing regulation, and movement complexity [14,50,51,52,53,82]. Resistance training may influence cognition through muscular strength, metabolic regulation, functional reserve, inflammatory regulation, and neurotrophic or myokine-related pathways [54,55,56,58,77]. These modality-specific pathways matter clinically because exercise programs are often selected according to functional capacity, safety, adherence, accessibility, and supervision needs.

Despite this clinical promise, most cognition-focused studies did not measure cerebrovascular or vascular mechanisms. Cognitive changes after exercise training cannot, therefore, be confidently attributed to cerebrovascular adaptation alone. Changes in global cognition, executive function, memory, attention, processing speed, cognitive-motor performance, or functional cognition may reflect multiple mechanisms, including improved fitness, enhanced mobility, greater social engagement, better sleep, reduced depressive symptoms, improved metabolic regulation, increased confidence, or practice effects from repeated cognitive testing [3,4,5,83,84]. Without concurrent vascular or cerebrovascular assessment, it remains difficult to determine whether observed cognitive changes are mediated by vascular adaptation or by other physiological, behavioral, or psychosocial pathways [6,8,9,11,72,73,76].

This mechanistic underlinking is a key finding of the evidence map. Cognition-only studies are valuable for determining where exercise interventions have been evaluated in relation to cognitive outcomes, while cerebrovascular-only studies are valuable for identifying vascular and hemodynamic adaptation. However, neither approach alone can determine whether vascular adaptation explains cognitive response. A more mechanism-informed approach is needed, in which cognitive outcomes are selected according to the expected mechanism of the intervention rather than added as generic secondary measures. Such designs would help determine whether different exercise modalities influence cognition through shared or distinct vascular, neural, metabolic, behavioral, and functional pathways [18,19,72,73,85]. From a healthcare translation perspective, this integration is necessary before cognitive findings can be converted into precision exercise prescriptions for specific cognitive-risk or vascular-risk populations.

### 4.4. Resistance, Multimodal, Mind–Body, and Dual-Task Exercise: Clinical Promise and Mechanistic Gaps

Beyond aerobic training, several modalities showed clinical relevance for cognitive health but were less frequently examined with direct cerebrovascular assessment. Resistance training, multimodal or multicomponent exercise, mind–body exercise, dual-task exercise, coordinative exercise, exergaming, and rehabilitation-based interventions were represented across older adults and cognitively or functionally at-risk populations [13,14,30,31,32,40,41,42,43,45,46,47,50,51,52,53,54,55,56,57,58,61,62,63,64,65,66,67]. Their value should not be judged only by whether they reproduce the vascular profile of aerobic training. These modalities may support cognition through complementary pathways involving strength, mobility, balance, attention, motor-cognitive integration, metabolic regulation, self-regulation, social engagement, and functional independence [77,78,79,80,81,82].

Resistance training may support cognitive health through mechanisms that extend beyond traditional endurance-based vascular adaptation. Improvements in muscular strength, functional capacity, insulin sensitivity, metabolic regulation, inflammatory profile, and anabolic or myokine-related signaling may indirectly influence brain health and cognitive performance [77]. In older adults and individuals with mild cognitive impairment or cognitive frailty, resistance training may also help preserve mobility, independence, and resilience against functional decline [54,55,56,58]. However, in the evidence map, resistance training contributed mainly to grouped cognitive domains, especially global cognition and cognitive-motor or functional cognition, rather than to direct cerebrovascular outcome domains. This indicates that its effects on cerebral blood flow or perfusion, cerebrovascular reactivity or hemodynamics, cerebral oxygenation, vascular function, and neurovascular coupling remain insufficiently characterized.

Multimodal and multicomponent exercise programs formed one of the largest intervention categories [13,31,32,40,41,42,43,45,57,65,66,67]. They combine aerobic exercise, resistance training, balance, flexibility, coordination, functional training, and cognitive-motor tasks, and may better reflect real-world practice than single-modality trials [78,79]. However, this complexity also makes mechanistic interpretation difficult because the active components, optimal dose, progression strategy, and interactions among components are often unclear. These programs were also more often represented in cognitive or functional outcome domains than in direct cerebrovascular assessment.

Mind–body exercise and dual-task, coordinative, or exergaming interventions represent additional approaches relevant for cognitive aging and healthcare delivery [14,30,46,47,50,51,52,53,61,62,63,80,81,82]. These modalities may influence cognition through pathways that are not purely cardiovascular, including attentional regulation, sensorimotor integration, postural control, balance, breathing regulation, self-regulation, task-specific learning, and cognitive-motor challenge. They may also be more acceptable or adaptable for some older adults because they can be delivered in group-based, home-based, low-impact, or community settings. In this context, the absence of direct cerebrovascular assessment should be interpreted as a mechanistic gap, not as evidence that these modalities lack relevance for brain health. Their potential role in dementia risk mitigation may depend on pathways that differ from, overlap with, or complement aerobic mechanisms.

Taken together, non-aerobic modalities should not be viewed as secondary to brain health research. The current limitation is the limited use of integrated outcome batteries capable of distinguishing vascular, neural, metabolic, functional, and behavioral mechanisms. Future studies should combine cerebrovascular or vascular measures with domain-specific cognitive testing, physical function outcomes, adherence monitoring, safety reporting, feasibility evaluation, and detailed dose reporting. Such work would clarify whether these modalities support cognition through vascular adaptation, cognitive-motor stimulation, functional enhancement, metabolic regulation, psychosocial engagement, or combined mechanisms [18,19,72,73,85].

### 4.5. The Need to Integrate Cerebrovascular and Cognitive Outcomes

A central finding of this review is that cerebrovascular and cognitive outcomes have not been sufficiently integrated within exercise intervention research [24,25,28,31,33,34,61]. This limited integration matters because exercise is often proposed to preserve cognition through vascular and cerebrovascular mechanisms, yet most intervention studies do not directly test this pathway [6,8,9,11,72,73,76]. Cognitive outcomes were distributed across many exercise modalities, whereas cerebrovascular and brain-related outcomes were concentrated in fewer modality-outcome domains. The evidence base, therefore, remains divided between cognition-focused trials without vascular assessment and cerebrovascular-focused studies without direct evaluation of cognitive response.

This division also reflects a methodological asymmetry across the field. Many cognition-focused studies were designed as randomized or controlled trials with larger samples and clinically relevant follow-up periods, but they often lacked direct cerebrovascular or vascular outcome assessment. Conversely, studies that included direct cerebrovascular measures were frequently smaller mechanistic studies, pilot trials, secondary analyses, or analyses based on outcome-specific subsamples. This imbalance has important implications for interpretation. Larger cognitive trials may provide stronger evidence for clinical feasibility and cognitive change, but they cannot determine whether those changes are mediated by cerebrovascular adaptation. Cerebrovascular studies may offer more direct physiological insight, but their smaller samples, selective analytic cohorts, shorter follow-up, and heterogeneous measurement protocols increase vulnerability to imprecision, selection bias, attrition bias, and limited generalizability. As a result, the current literature remains poorly positioned to establish whether exercise-induced cerebrovascular changes are causal mechanisms, parallel physiological adaptations, or unrelated correlates of cognitive improvement.

Future trials should test the proposed vascular-cognitive pathway more directly. Rather than treating cerebrovascular and cognitive outcomes as separate endpoints, studies should examine whether changes in perfusion, cerebrovascular reactivity, cerebral oxygenation, vascular function, or neurovascular coupling correspond to changes in domain-specific cognitive performance. Cognitive outcomes should be selected according to the expected mechanism of the intervention rather than added as generic secondary measures. For example, aerobic training trials may prioritize domains related to perfusion, vascular responsiveness, and cardiometabolic adaptation, whereas resistance, multimodal, mind–body, dual-task, or exergaming trials may additionally assess cognitive-motor performance, functional cognition, mobility-related cognition, and daily functioning.

Integrated designs should also report exercise dose, adherence, safety, feasibility, and relevant mediators. Based on these findings, future trials should adopt a minimum reporting standard for exercise prescription and implementation. At minimum, studies should report exercise frequency, intensity targets and monitoring methods, session duration, exercise type, weekly or total training volume, progression rules, supervision level, adherence, dropout, adverse events, comparator condition, and the timing of cerebrovascular and cognitive outcome assessments. Without these elements, it remains difficult to compare exercise doses across studies, evaluate dose–response patterns, replicate intervention protocols, or translate findings into healthcare-oriented exercise prescription. Frequency, intensity, time, type, progression, supervision, and adherence help determine whether null or mixed findings reflect ineffective interventions, inadequate dose, poor compliance, insensitive outcomes, limited feasibility, or true absence of effect [18,19,85]. Dose–response interpretation also remains limited in the current evidence base. Longer and progressively intensified aerobic interventions were more often represented among studies assessing cerebrovascular outcomes, particularly those examining cerebral blood flow, cerebrovascular reactivity, arterial stiffness, or hemodynamic regulation. These studies suggest that sustained aerobic exposure and improved cardiorespiratory fitness may be relevant to cerebrovascular adaptation, but the direction and magnitude of responses were not uniform across outcomes, populations, or measurement tools. In cognition-focused studies, intervention duration, intensity, and progression varied widely across resistance training, multimodal exercise, mind–body exercise, dual-task or exergaming interventions, and rehabilitation-based programs. Some studies suggested that higher adherence, longer exposure, or progressive task difficulty may support cognitive gains, especially in executive function, attention, memory, or cognitive-motor outcomes. However, the evidence was not sufficient to establish a clear dose–response relationship because many studies differed in baseline risk profile, supervision, comparator condition, adherence, outcome sensitivity, and timing of post-intervention assessment. Future trials should therefore report FITT-VP elements, adherence, progression criteria, and outcome timing in sufficient detail to determine whether cerebrovascular and cognitive benefits depend on exercise intensity, cumulative duration, progression, or achieved physiological adaptation. Fitness, strength, balance, mobility, physical function, and biological markers related to inflammation, oxidative stress, neurotrophic signaling, metabolic regulation, or vascular function may further clarify whether cognitive changes arise through vascular, neural, metabolic, functional, behavioral, or psychosocial pathways [3,4,5,83,84].

Such designs would move the field beyond simple pre-post comparisons and toward more explanatory models of exercise response. They could clarify whether cerebrovascular adaptation mediates cognitive improvement, reflects broader physiological adaptation, or occurs as a parallel response without measurable cognitive benefit [24,25,28,31,33,34,61,72,73]. This distinction matters because improvement in a vascular marker cannot be assumed to produce cognitive change, particularly when studies differ in baseline vascular risk, cognitive reserve, intervention duration, dose, supervision, adherence, and cognitive assessment sensitivity. Future research should therefore identify which modalities, doses, delivery models, mechanisms, and populations are most likely to produce vascular-cognitive benefits.

### 4.6. Toward Precision Exercise Prescription for Brain Health

The findings support a shift from general physical activity promotion toward mechanism-informed exercise prescription for brain health. Exercise modalities should not be treated as interchangeable because they may influence brain health through partly distinct vascular, metabolic, neuromuscular, cognitive-motor, psychosocial, and behavioral pathways. Prescriptions should therefore be guided by target population, baseline risk profile, intended outcome domain, hypothesized mechanism, supervision requirement, safety profile, and feasibility of long-term adherence [5,10,18,19,84,85].

For adults with mild cognitive impairment or early cognitive vulnerability, multimodal or multicomponent exercise may be particularly relevant because it can combine aerobic, resistance, balance, coordination, flexibility, functional, and cognitive-motor elements within a single program [13,31,32,40,41,42,43,45,57,65,66,67]. For adults with vascular or cardiometabolic risk, aerobic or combined aerobic and resistance training may be more appropriate when the primary targets include blood pressure control, cardiorespiratory fitness, endothelial function, arterial stiffness, metabolic health, cerebral blood flow, or brain perfusion [25,28,33,34,35,69,70,71,74,75,76]. For frail older adults or adults with cognitive frailty, resistance training, balance training, dual-task exercise, and multimodal exercise may be especially relevant because they target strength, mobility, fall risk, cognitive-motor function, and functional independence [54,55,61,77]. These examples show that exercise prescription should be matched to both biological pathways and functional needs.

For sedentary community-dwelling older adults, progressive aerobic training remains a practical foundation for improving cardiorespiratory fitness and potentially supporting cerebrovascular health [25,26,27,33,34,36,74,75]. However, adherence, supervision, intensity progression, behavioral support, and safety monitoring remain essential. A technically sound prescription may fail if participation is insufficient, intensity is too low, progression is poorly maintained, or the delivery model is not feasible. Dose, adherence, progression, supervision, adverse events, and feasibility should therefore be reported clearly to distinguish insufficient implementation from true lack of biological or cognitive effect [18,19,85,89,90].

Overall, exercise prescription for brain health should consider five interacting dimensions: modality, dose, population, outcome target, and delivery context. Modality should be linked to the hypothesized mechanism, dose should be described using FITT and progression elements, population characteristics should include cognitive status, vascular risk, functional capacity, frailty, baseline activity, comorbidities, and feasibility of sustained participation, and outcome targets should include cognitive, cerebrovascular, physiological, behavioral, and functional measures. Delivery context should specify whether the intervention is intended for laboratory testing, outpatient rehabilitation, community-based prevention, home-based delivery, or long-term care. This framework would support more risk-stratified and clinically usable exercise prescriptions for dementia risk mitigation and brain health promotion [18,19,84,85].

### 4.7. Methodological Gaps and Future Research Priorities

The methodological mapping identified several gaps that limit interpretation, replication, and clinical implementation. One major issue was inconsistent exercise prescription reporting. Although most studies described modality and duration, details regarding frequency, intensity, session duration, progression, supervision, adherence, and dose modification were not consistently reported. Because cerebrovascular and cognitive adaptations are likely dose-sensitive, incomplete FITT reporting makes it difficult to determine whether null or mixed findings reflect inadequate dose, poor adherence, insufficient progression, insensitive outcomes, limited feasibility, or true absence of effect [18,19,85].

Adherence, safety, and feasibility reporting also require greater standardization, particularly in older adults and populations with mild cognitive impairment, frailty, cardiometabolic risk, or cardiovascular disease. Attendance, compliance with prescribed intensity, progression achieved, reasons for dropout, adverse events, safety-related modifications, and adherence-support strategies determine whether an intervention can be feasible, acceptable, and sustainable outside controlled settings [18,89]. These issues are especially important in rehabilitation, community-based prevention, home-based delivery, and long-term care.

Several studies were limited by small samples, pilot designs, short intervention durations, or lack of long-term follow-up. These constraints are understandable in mechanistic cerebrovascular research, which often requires technically demanding assessments such as cerebral perfusion imaging, transcranial Doppler ultrasound, near-infrared spectroscopy, or cerebrovascular reactivity testing. However, small and short-term designs reduce confidence in the durability and clinical relevance of observed effects. Longer interventions and follow-up assessments are needed when feasible because short-term changes in vascular or cognitive outcomes may not indicate sustained protection against cognitive deterioration [90].

Outcome selection also remains heterogeneous. Cognitive outcomes were assessed using global screening tools, domain-specific neuropsychological tests, computerized batteries, cognitive-motor tasks, functional cognitive measures, dementia-related outcomes, and biomarker-linked outcomes. This diversity reflects the multidimensional nature of cognition but complicates comparison across studies. Although this review grouped related outcomes into broader domains for Figure 3, future trials should still report original cognitive tests and domain-specific outcomes clearly to preserve transparency and reproducibility.

A more mechanism-informed approach to cognitive testing is needed. Studies focused on vascular or cerebrovascular adaptation should prioritize domains sensitive to vascular cognitive aging, including executive function, processing speed, attention, working memory, and cognitive flexibility. Global screening tools such as MMSE or MoCA remain useful for clinical characterization, but they may not capture the full pattern of domain-specific cognitive change expected after exercise training [6,7,83]. Cerebrovascular outcomes should likewise be selected according to the hypothesized intervention mechanism. For example, aerobic exercise studies may prioritize cerebral blood flow or perfusion, cerebrovascular reactivity or hemodynamics, vascular function, arterial stiffness, or blood pressure-related indicators, whereas dual-task, exergaming, or multimodal interventions may also require measures of neurovascular coupling, brain activation, functional cognition, and cognitive-motor performance.

Integrated mechanistic assessment and subgroup analysis remain important priorities. At present, many studies do not combine exercise dose reporting, cerebrovascular assessment, cognitive testing, cardiorespiratory fitness or strength outcomes, adherence monitoring, safety reporting, feasibility evaluation, and biological markers within the same design. This limits the ability to distinguish whether cognitive change is related primarily to vascular adaptation, neurotrophic signaling, inflammatory regulation, metabolic improvement, functional gains, behavioral engagement, psychosocial effects, or combined pathways [72,73,84]. Greater attention to sex-specific, risk-specific, and population-specific responses is also needed because exercise adaptations may vary according to sex, age, menopausal status, baseline fitness, vascular risk burden, cognitive status, frailty, medication use, comorbidities, and metabolic health. Moving beyond average treatment effects would strengthen the basis for precision exercise prescription and improve the clinical relevance of exercise-based strategies for cerebrovascular and cognitive health. These reporting and design gaps are reflected in the study-level coding and grouped evidence-map domains provided in Supplementary Tables S8 and S9.

### 4.8. Strengths and Limitations

This scoping review has several strengths. First, the review was guided by the PRISMA-ScR framework and used a predefined PCC structure to organize eligibility criteria, data charting, and evidence mapping [15,16]. This approach was appropriate because the review aimed to map the breadth, distribution, and translational readiness of the evidence rather than estimate pooled intervention effects. Second, the search was conducted systematically in PubMed/MEDLINE and Scopus and was supplemented by reference list checking and citation chasing. Third, the review included 54 intervention studies and organized them according to outcome-integration categories, including cerebrovascular or vascular outcomes only, cognitive outcomes only, and studies assessing both cerebrovascular and cognitive outcomes within the same intervention design [13,14,20,21,22,23,24,25,26,27,28,29,30,31,32,33,34,35,36,37,38,39,40,41,42,43,44,45,46,47,48,49,50,51,52,53,54,55,56,57,58,59,60,61,62,63,64,65,66,67,68,69,70,71]. This classification directly addressed whether the current literature links vascular adaptation with cognitive outcomes, which is central to mechanism-informed exercise prescription.

A further strength is that this review did not treat exercise as a single homogeneous exposure. Instead, it distinguished major exercise modalities, including aerobic training, resistance training, combined training, multimodal or multicomponent exercise, mind–body exercise, dual-task or exergaming exercise, and rehabilitation-based exercise. This modality-specific approach made it possible to identify where evidence is concentrated and where important outcome-integration gaps remain. Another strength is the use of grouped cerebrovascular and cognitive outcome domains in the evidence map. Original study-level outcome labels were retained in the supplementary coding tables, while closely related outcomes were grouped into broader domains for Figure 3 to improve interpretability and reduce excessive fragmentation. This approach strengthened the transparency and readability of the evidence map while preserving traceability between the original extracted outcomes and the final grouped visualization.

Several limitations should also be acknowledged. First, no meta-analysis was conducted. This decision was consistent with the scoping review objective, but it means that the review does not provide pooled effect estimates for specific exercise modalities, populations, or outcomes [15,16]. The included studies varied substantially in population characteristics, exercise prescription, intervention duration, comparator conditions, outcome measures, and assessment tools, limiting the appropriateness of quantitative synthesis. Therefore, the findings should be interpreted as a map of evidence distribution, outcome integration, methodological reporting, and healthcare translation gaps rather than as a quantitative estimate of intervention effectiveness. Relatedly, no formal risk-of-bias assessment or certainty-of-evidence grading was performed. Although methodological strength, FITT reporting quality, evidence maturity, and translational readiness were charted descriptively, these categories should not be interpreted as substitutes for formal methodological quality appraisal. This limits the ability to draw conclusions about internal validity, effect credibility, causal certainty, or comparative effectiveness across studies.

Another methodological limitation is that the OSF registration was retrospective. The final database search update was completed on 4 May 2026, whereas the OSF registration was completed on 29 May 2026. Although the registration documented the review objective, PCC-based eligibility criteria, search strategy, data charting framework, evidence-mapping approach, and descriptive synthesis plan, it was not a prospective protocol registration completed before searching. This timing may increase concerns about selective reporting or post hoc methodological decisions, and the findings should be interpreted with this limitation in mind.

In addition, the search was limited to English-language peer-reviewed articles indexed in PubMed/MEDLINE and Scopus. Although these databases provide strong coverage of biomedical, clinical, neuroscience, rehabilitation, exercise science, and interdisciplinary health-related literature, studies indexed only in other specialized databases may have been missed. This limitation is particularly relevant to rehabilitation, allied health, nursing, psychology, mental health, behavioral intervention, and community-based exercise research, where relevant studies may be indexed in CINAHL, PsycINFO, Embase, Web of Science, or other specialized databases rather than PubMed/MEDLINE or Scopus alone. As a result, the evidence map may underrepresent some rehabilitation-based, psychosocial, behavioral, functioning-oriented, mental health-related, or community-based exercise interventions, especially those emphasizing participation, daily function, adherence, self-management, mood, or community implementation rather than cerebrovascular or biomedical outcomes. Reference list checking and citation chasing were used to reduce this risk, but they cannot fully replace broader database coverage. Accordingly, some relevant studies, particularly those published in psychology, rehabilitation, behavioral health, allied health, or interdisciplinary implementation journals, may not have been captured. Publication bias should also be considered when interpreting the evidence map. Studies using expensive or technically demanding cerebrovascular and neuroimaging assessments may be more likely to involve small samples, selective analytic subsamples, or mechanistic outcomes, and studies reporting positive or biologically coherent findings may be more likely to reach publication than null or inconclusive studies. As a result, the mapped evidence landscape may overrepresent favorable or mechanistically interpretable exercise-related findings and underrepresent unpublished, null, or less conclusive results. Because this scoping review did not conduct a meta-analysis or formal publication-bias assessment, the evidence map should be interpreted as a distribution of published evidence rather than as an unbiased estimate of the full research landscape.

Third, a rule-based prescreening step was applied after deduplication to improve feasibility and align the large search set with the PCC framework. The rules were applied conservatively, and records with uncertain eligibility were retained for title and abstract screening whenever possible. Nevertheless, this non-standard prescreening step may have introduced selection bias despite these safeguards. Some relevant studies may have been missed if titles or abstracts used non-standard terminology or provided limited information about the population, intervention, or outcome. The prescreening logic is therefore reported in Supplementary Table S3 to support transparency, including inclusion signals, exclusion signals, uncertainty rules, and examples of retained and excluded records.

Fourth, some included studies involved exercise-based lifestyle or rehabilitation programs rather than exercise-only interventions [68,69,70,71]. These studies were retained when structured exercise was a central component and eligible cognitive, cerebrovascular, vascular, or brain-related outcomes were reported. However, they should be interpreted separately from trials isolating exercise as the sole intervention component, because cognitive or vascular outcomes may also have been influenced by education, diet, clinical care, behavioral counseling, risk-factor management, or broader rehabilitation support. This issue is particularly relevant for healthcare translation, where multicomponent delivery may improve ecological validity but complicate causal interpretation.

Finally, the descriptive methodological quality mapping should be interpreted as an evidence-mapping aid rather than as a formal appraisal of study validity. This approach is consistent with the purpose of a scoping review, which is to characterize the breadth and distribution of evidence rather than judge comparative effectiveness [15,16]. However, the absence of a formal risk-of-bias assessment limits conclusions about the internal validity of individual studies. Accordingly, the findings should be understood as an evidence map of current research activity, outcome-integration patterns, methodological reporting features, and future research priorities, rather than as definitive evidence that any specific exercise modality improves cerebrovascular or cognitive outcomes. Future systematic reviews or meta-analyses may be appropriate when more studies use comparable populations, exercise prescriptions, outcome domains, and integrated vascular-cognitive assessment designs.

## 5. Conclusions

This scoping review mapped 54 intervention studies on structured exercise training, cerebrovascular outcomes, and cognitive health in adults at risk of cognitive decline. The evidence indicates that exercise training has been widely investigated as a strategy for supporting cognitive health, but cerebrovascular and cognitive endpoints remain insufficiently integrated within the same intervention designs. In the grouped evidence map, cognitive outcomes were represented across a broad range of exercise modalities, whereas cerebrovascular and brain-related outcomes were concentrated mainly in aerobic or aerobic-based interventions.

Aerobic training currently represents the most developed modality for studying exercise-related cerebrovascular adaptation. However, resistance training, multimodal or multicomponent exercise, mind–body exercise, dual-task or exergaming approaches, and rehabilitation-based exercise remain comparatively underexamined using direct cerebrovascular measures, despite their relevance for older adults and populations with cognitive, functional, vascular, or cardiometabolic risk. This imbalance suggests that the current evidence base is not yet sufficient to support strong modality-specific precision exercise prescriptions for cerebrovascular and cognitive health.

A key implication of this evidence map is that cognitive change after exercise cannot be assumed to reflect cerebrovascular adaptation unless both domains are assessed within the same intervention design. Future studies should examine vascular, cognitive, physiological, behavioral, and functional mediators within the same intervention logic rather than treating cognitive change as a stand-alone endpoint. Such designs are needed to clarify whether different exercise modalities support cognitive health through shared or distinct vascular, neural, metabolic, functional, psychosocial, or behavioral pathways.

Overall, structured exercise training remains a promising healthcare-relevant strategy for brain health promotion and dementia risk mitigation. The next stage of research should move beyond the general question of whether exercise benefits cognition and toward a more precise understanding of which exercise modalities, doses, mechanisms, and delivery models are most appropriate for specific populations, risk profiles, care settings, and outcome targets. This precision-oriented approach may strengthen the translation of exercise training into practical, mechanism-informed interventions for aging, cognitive-risk, vascular-risk, rehabilitation, and community prevention contexts. Because vascular and cognitive outcomes are routinely assessed in separate studies or separate analytic frameworks, the field cannot yet establish a definitive mechanistic pathway linking exercise-induced cerebrovascular adaptations to actual cognitive improvements. Future exercise trials should therefore integrate cerebrovascular assessment, domain-specific cognitive testing, standardized measurement approaches, detailed FITT-VP exercise prescription reporting, adherence and safety monitoring, and follow-up assessment within the same intervention designs to determine whether vascular adaptations precede, accompany, mediate, or merely correlate with cognitive change.

## Figures and Tables

**Table 1:** Concise Population, Concept, and Context framework and eligibility criteria.

PCC Component	Eligibility Focus	Main Exclusion Criteria
Population	Adults aged 18 years or older, with emphasis on middle-aged adults, older adults, sedentary or physically inactive adults, and adults at elevated risk of cognitive decline. Eligible risk profiles included cognitive, vascular, cardiometabolic, or functional risk factors relevant to cerebrovascular or cognitive health, such as mild cognitive impairment, subjective cognitive decline, hypertension, obesity, type 2 diabetes, metabolic syndrome, cardiovascular risk, frailty, cognitive frailty, and sedentary behavior.	Children or adolescents only; animal or cell models; exclusively elite athletic populations without relevance to cerebrovascular or cognitive health; populations with conditions unrelated to cerebrovascular or cognitive health, unless eligible outcomes were directly reported.
Concept	Repeated structured exercise training designed to induce physiological, functional, cerebrovascular, or cognitive adaptations. Eligible interventions included aerobic, resistance, combined aerobic and resistance, high-intensity interval, multimodal or multicomponent, mind–body, dual-task, coordinative, balance-oriented, exergaming, and rehabilitation-based exercise programs.	Acute single-session exercise only; observational physical activity studies without a structured training intervention; sedentary behavior studies without exercise intervention; diet-only, drug-only, cognitive-training-only, or non-exercise interventions; interventions in which exercise was not a primary or separable component.
Context	Community, laboratory, university, outpatient, rehabilitation, health promotion, home-based, and aging-related prevention settings in which structured exercise training was delivered and cerebrovascular, vascular, brain-related, or cognitive outcomes were assessed.	Inpatient acute care, surgical-only, pharmacological-only, or non-exercise clinical settings, unless a structured exercise training intervention was tested.
Outcomes	Studies were eligible if they reported at least one cerebrovascular, vascular, brain-related, or cognitive outcome relevant to cognitive aging, dementia risk, or brain health. Eligible outcomes included cerebral blood flow or perfusion, cerebral blood velocity, cerebrovascular reactivity, cerebral oxygenation, neurovascular coupling, endothelial or vascular function, arterial stiffness, blood pressure-related vascular indicators, global cognition, executive function, memory, attention, processing speed, cognitive-motor performance, MMSE, and MoCA.	Studies without eligible cerebrovascular, vascular, brain-related, cognitive, neuropsychological, or dementia-related outcomes; studies reporting only peripheral fitness or general cardiovascular outcomes without relevance to brain health or cognitive risk.
Study design and publication characteristics	Original human intervention studies, including randomized controlled trials, non-randomized controlled trials, quasi-experimental studies, controlled pre-post studies, pilot trials, feasibility trials, and single-arm intervention studies. Eligible records were peer-reviewed full-text articles published in English between January 2010 and 4 May 2026.	Reviews, systematic reviews, meta-analyses, editorials, commentaries, letters, protocols without results, conference abstracts without full-text data, case reports, cross-sectional studies without training intervention, non-English articles, unavailable full texts, grey literature, theses, and dissertations.

Table note: PCC = Population, Concept, and Context; MMSE = Mini-Mental State Examination; MoCA = Montreal Cognitive Assessment. Studies were eligible if they included adult participants, repeated structured exercise training, and at least one cerebrovascular, vascular, brain-related, or cognitive outcome relevant to cognitive aging or dementia risk. Studies assessing both cerebrovascular and cognitive outcomes were highlighted for outcome-integration analysis.

**Table 2:** Summary of included studies by category.

Category	Number of Studies	Representative Exercise Modalities	Main Outcome Domains
Total included studies	54	Aerobic training; resistance training; combined training; multimodal exercise; mind–body exercise; dual-task training; exergaming; rehabilitation-based exercise	Cerebrovascular function; cognitive function; brain structure or activation; vascular-risk-related cognitive outcomes
Cerebrovascular outcomes only	9	Aerobic training; dual-task training; whole-body vibration; multicomponent exercise; clinical exercise intervention	Cerebral blood flow; cerebral perfusion; cerebral blood velocity; cerebrovascular reactivity; cerebral oxygenation; cerebral hemodynamics
Cognitive outcomes only	38	Aerobic exercise; resistance training; multicomponent exercise; Tai Chi; yoga; exergaming; dual-task exercise; functional task exercise	Global cognition; executive function; memory; attention; processing speed; cognitive decline prevention; MoCA; MMSE
Both cerebrovascular and cognitive outcomes	7	Aerobic training; aquatic treadmill exercise; aerobic exercise with nutritional co-intervention; whole-body vibration; exercise training in MCI or sedentary older adults	Cerebral blood flow or perfusion combined with cognition; cerebrovascular reactivity and cognitive function; cerebral oxygenation and cognitive outcomes
Aerobic-focused studies	17	Aerobic exercise; aerobic dance; treadmill exercise; aquatic treadmill exercise; walking or endurance-based training	Cerebral blood flow; brain perfusion; cerebrovascular reactivity; global cognition; executive function; memory
Resistance-focused studies	4	Progressive resistance training; strength training; home-based resistance exercise; resistance training in cognitive frailty or MCI	Cognitive function; executive function; cortical thickness; frailty-related cognitive outcomes; strength-related cognitive adaptation
Combined training studies	6	Combined aerobic and resistance training; combined physical-cognitive exercise; integrated exercise programs	Global cognition; executive function; memory; mobility-cognition outcomes; functional cognition
Multimodal or multicomponent exercise studies	12	Multicomponent exercise; functional task exercise; multimodal physical therapy; day-care exercise programs; exercise programs combining aerobic, resistance, balance, flexibility, or coordination components	Global cognition; executive function; mobility-cognition outcomes; functional cognition; brain activation
Mind–body exercise studies	5	Tai Chi; yoga; qigong-related or integrative mind–body approaches	Cognitive function; memory; executive function; brain-derived neurotrophic factor (BDNF) or immunological markers; fall-risk-related cognitive outcomes
Dual-task, coordinative, or exergaming studies	6	Dual-task training; virtual reality-based exercise; exergame balance training; SMARTfit training; cognitive-motor exercise	Cognitive function; cognitive-motor integration; brain activation; balance-related cognitive outcomes
Rehabilitation-based exercise studies	4	Cardiac rehabilitation; exercise-based lifestyle intervention; clinical rehabilitation exercise; structured exercise within broader clinical care	Cognitive function; vascular-risk-related cognition; functional outcomes in cardiovascular or clinical-risk populations
Vascular-risk or cardiometabolic-risk populations	6	Aerobic training; lifestyle intervention including exercise; cardiac rehabilitation; clinical rehabilitation exercise	Cognitive function in hypertension, cardiovascular disease, heart failure, obesity, or metabolic risk contexts
MCI or cognitive-risk populations	37	Aerobic exercise; resistance training; multicomponent exercise; Tai Chi; yoga; exergaming; dual-task exercise	Global cognition; executive function; memory; MoCA; MMSE; cognitive decline prevention; brain structure or perfusion
Studies with mechanistic or biological markers	11	Aerobic training; Tai Chi; yoga; resistance training; multimodal exercise	BDNF; cerebral oxygenation; cortical thickness; hippocampal volume; brain activation; vascular stiffness; cerebrovascular reactivity

Table note: Values summarize the 54 included studies. Counts are descriptive and based on study-level evidence-map coding; some studies may contribute to more than one category because they included multiple exercise components, population risk features, or outcome domains. MCI = mild cognitive impairment; MMSE = Mini-Mental State Examination; MoCA = Montreal Cognitive Assessment. Detailed study-level coding is provided in Supplementary Table S9.

**Table 3:** Evidence map coding framework for included studies.

Coding Domain	Main Categories	Coding Purpose	Use in Evidence Map
Exercise modality	Aerobic training; resistance training; combined aerobic and resistance training; high-intensity interval training; multimodal or multicomponent exercise; mind–body exercise; dual-task, coordinative, or exergaming exercise; rehabilitation-based exercise.	To classify the primary type of structured exercise training or exercise-based intervention used in each included study.	Used as the main intervention classification for mapping exercise modalities against grouped cerebrovascular and cognitive outcome domains.
Population category	Healthy adults; healthy older adults; sedentary or inactive adults; mild cognitive impairment; subjective cognitive decline; cognitive frailty or frailty risk; cardiometabolic or vascular risk.	To identify the target population and the level of risk for cognitive decline or cerebrovascular dysfunction.	Used to describe whether evidence is concentrated in healthy aging populations, cognitively at-risk groups, or vascular or metabolic risk groups.
Cerebrovascular or brain-related outcome domain	Brain structure or other brain-related surrogate outcomes; cerebral blood flow or perfusion; cerebrovascular reactivity or hemodynamics; cerebral oxygenation; vascular function, arterial stiffness, or blood pressure-related indicators; neurovascular coupling or brain activation.	To group cerebrovascular, vascular, and brain-related outcomes into clinically and mechanistically meaningful domains relevant to brain health and healthcare translation.	Used for Panel A of Figure 3, which summarizes exercise modalities by grouped cerebrovascular outcome domains.
Cognitive outcome domain	Global cognition; executive function; memory; attention or processing speed; cognitive-motor or functional cognition; dementia-related screening or decline prevention; biomarker-linked or brain-health-related cognitive outcomes.	To classify cognitive outcomes into interpretable domains relevant to aging, vascular cognitive risk, dementia prevention, rehabilitation, and healthcare-oriented exercise prescription.	Used for Panel B of Figure 3, which summarizes exercise modalities by grouped cognitive outcome domains.
Outcome integration category	Cerebrovascular or brain-related outcomes only; cognitive outcomes only; both cerebrovascular or brain-related and cognitive outcomes.	To determine whether included studies assessed vascular mechanisms, cognitive outcomes, or both within the same intervention design.	Used to highlight studies that directly link exercise-related cerebrovascular adaptation with cognitive health.
Direction of findings	Positive; mixed; null; negative; unclear.	To summarize the dominant direction of findings for each study or evidence map cell.	Coded descriptively to support interpretation of the evidence map, but not used as a primary visual encoding variable in Figure 3.
Methodological and reporting maturity	Higher; moderate; preliminary.	To contextualize evidence maturity and reporting completeness based on study design, comparator condition, exercise prescription clarity, adherence reporting, feasibility information, and outcome assessment.	Coded descriptively to contextualize evidence maturity, reporting completeness, and translational readiness, but not used as a primary visual encoding variable in Figure 3.
Exercise dose reporting quality	Complete; partial; limited.	To evaluate whether exercise prescription details were sufficiently reported for interpretation, replication, and healthcare translation.	Coded descriptively to identify reporting gaps related to frequency, intensity, time, type, progression, supervision, and adherence.

Table note: Original study-level outcome labels were retained during data charting, while closely related cerebrovascular, vascular, brain-related, and cognitive outcomes were grouped into broader domains for Figure 3 visualization. Full operational definitions and coding rules are provided in Supplementary Table S8, and detailed study-level coding is provided in Supplementary Table S9.

**Table 4:** Cross-tabulation of cerebrovascular and cognitive outcome integration across the 54 included studies.

Cerebrovascular or Vascular Outcomes	Cognitive Outcomes Assessed	Number of Studies
Yes	Yes	7
Yes	No	9
No	Yes	38

## Data Availability

All data analyzed in this scoping review were extracted from previously published studies cited in the manuscript and Supplementary Materials. No new datasets were generated.

## Supplementary Materials

The following supporting information can be downloaded at https://www.mdpi.com/article/10.3390/healthcare14121774/s1, Table S1: Database-specific search strategies; Table S2: Search log; Table S3: Detailed PCC framework, eligibility criteria, examples, screening notes, and prescreening logic; Table S4: Full-text exclusion reasons; Table S5: Data charting framework; Table S6: Exercise intervention characteristics; Table S7: Cerebrovascular, brain-related, and cognitive outcome domains; Table S8: Evidence map coding framework; Table S9: Study-level characteristics and evidence-map coding; Table S10: PRISMA-ScR checklist with section-based manuscript references.

## Abbreviations

The following abbreviations are used in this manuscript:

AER	Aerobic training
BDNF	Brain-derived neurotrophic factor
BP	Blood pressure
COM	Combined training
DTE	Dual-task, coordinative, or exergaming exercise
FITT	Frequency, intensity, time, and type
HIIT	High-intensity interval training
MBE	Mind–body exercise
MCI	Mild cognitive impairment
MME	Multimodal or multicomponent exercise
MMSE	Mini-Mental State Examination
MoCA	Montreal Cognitive Assessment
PCC	Population, Concept, and Context
RET	Resistance training
