# Supplementary material for: Exercise Training for Cerebrovascular and Cognitive Health in Adults at Risk of Cognitive Decline: A Scoping Review of Healthcare Translation and Evidence Gaps
Source: Healthcare (Basel). 2026 Jun 19;14(12):1774. doi: 10.3390/healthcare14121774 (PMC13299165; doi:10.3390/healthcare14121774)
Supplement: Supplementary file 1 [file healthcare-14-01774-s001.zip › Supplementary Table S10_PRISMA_ScR_section_based_compact.pdf]

Supplementary Table S10. PRISMA-ScR checklist with section-based manuscript references

Stable manuscript section, table, figure, and supplementary-file references are used instead of page and line numbers to avoid mismatch after template formatting, revision, and production typesetting.

PRISMA-ScR item	Checklist focus	Stable manuscript location / reporting details
1	Title	Title identifies the article as a scoping review and evidence map.
2	Structured summary	Abstract reports background/objectives, methods, results, conclusions, eligibility framework, sources of evidence, mapping approach, included studies, and implications.
3	Rationale	Introduction explains dementia burden, cerebrovascular dysfunction, physical inactivity, exercise training, evidence heterogeneity, and rationale for a scoping review and evidence map.
4	Objectives and PCC elements	Final paragraph of the Introduction states the objective and scope: adults at risk of cognitive decline, structured exercise training, cerebrovascular/cognitive outcomes, grouped domains, outcome integration, and healthcare translation.
5	Protocol and registration	Methods, Section 2.1. Add or retain: “No review protocol was registered.” If a protocol is later registered, replace this with protocol access or registration information.
6	Eligibility criteria	Methods 2.2 and Table 1 define PCC-based eligibility, outcomes, designs, publication criteria, and exclusions; Supplementary Table S3 provides screening notes.
7	Information sources	Methods 2.3 reports PubMed/MEDLINE, Scopus, reference list checking, citation chasing, and final search date; Supplementary Table S2 provides the search log.
8	Search strategy	Methods 2.4 summarizes two search paths, terms, syntax, and filters; Supplementary Table S1 provides full database-specific strategies.
9	Selection process	Methods 2.5 describes Zotero/Rayyan management, deduplication, conservative PCC-based prescreening, title/abstract screening, full-text eligibility, uncertainty rules, and exclusion categories; Supplementary Tables S3–S4 provide details.
10	Data charting process	Methods 2.6 describes standardized charting, iterative refinement, checking against PCC and coding rules, and resolution of uncertainty; no investigator contact was used for data confirmation.
11	Data items	Methods 2.6 lists study, population, intervention, comparator, adherence, adverse event, outcome, marker, timing, finding, and implementation variables; Supplementary Tables S5–S7 define charted variables and outcome domains.
12	Critical appraisal rationale and use	Methods 2.8 explains that formal risk-of-bias assessment was not used for exclusion; methodological and reporting features were charted descriptively for evidence maturity and translational readiness; Supplementary Table S8 provides rules.
13	Synthesis of results	Methods 2.7 describes descriptive synthesis and two-panel evidence mapping by exercise modality, grouped outcome domains, evidence density, and outcome-integration category; Figure 3 presents the map.
14	Selection results	Results 3.1 and Figure 2 report records identified, duplicates, prescreening, screening, reports retrieved, full-text assessment, exclusions, and 54 included studies.
15	Characteristics of included studies	Results 3.2 and Table 2 summarize included studies; Supplementary Table S9 provides study-level characteristics, original outcome labels, grouped domains, and evidence-map coding.

PRISMA-ScR item	Checklist focus	Stable manuscript location / reporting details
16	Critical appraisal results	Results 3.8 presents descriptive methodological characteristics, reporting maturity, FITT reporting quality, adherence, supervision, and reporting gaps; not formal risk-of-bias ratings.
17	Results of individual sources	Results 3.2–3.8 present population, intervention, cerebrovascular/brain-related, cognitive, evidence-map, and methodological reporting results; Supplementary Table S9 provides study-level data.
18	Synthesis of charting results	Results 3.2–3.8 synthesize outcome-integration categories, exercise modalities, population risk profiles, grouped domains, evidence concentration, underrepresented combinations, and methodological gaps.
19	Summary of evidence	Discussion 4.1–4.7 summarize main concepts and evidence types, including vascular-cognitive separation, aerobic evidence cluster, mechanistic underlinking of cognition, non-aerobic gaps, integration needs, and future priorities.
20	Limitations	Discussion 4.8 addresses no meta-analysis, database/language restrictions, rule-based prescreening, lifestyle/rehabilitation programs, and descriptive rather than formal risk-of-bias assessment.
21	Interpretation and implications	Discussion 4.1–4.8 and Conclusions interpret findings for vascular-cognitive integration, healthcare translation, precision exercise prescription, and future trials.
22	Funding	Funding statement reports that the review received no external funding. Funding for individual included studies was not charted as a review variable.

Table note: PRISMA-ScR = Preferred Reporting Items for Systematic Reviews and Meta-Analyses extension for Scoping Reviews; PCC = Population, Concept, and Context; FITT = frequency, intensity, time, and type. Items 12 and 16 refer to descriptive methodological quality mapping, not formal risk-of-bias assessment.
