# Supplementary material for: Exercise Training for Cerebrovascular and Cognitive Health in Adults at Risk of Cognitive Decline: A Scoping Review of Healthcare Translation and Evidence Gaps
Source: Healthcare (Basel). 2026 Jun 19;14(12):1774. doi: 10.3390/healthcare14121774 (PMC13299165; doi:10.3390/healthcare14121774)
Supplement: Supplementary file 1 [file healthcare-14-01774-s001.zip › Supplementary Table S1_Search Strategies.pdf]

Supplementary Table S1. Complete database-specific search strategies

Manuscript: Exercise Training for Cerebrovascular and Cognitive Health in Adults at Risk of Cognitive Decline: A Scoping Review and Evidence Map

This table provides the complete database-specific search strategies used for PubMed/MEDLINE and Scopus. Search Path A targeted structured exercise training, cerebrovascular function, and adult or aging-related populations. Search Path B targeted structured exercise training, cognitive outcomes, and dementia, brain health, or aging-related contexts.

Database	Path	Search Purpose	Limits / Filters	Records Retrieved	Date Searched	Search Syntax
PubMed/MEDLINE	Path A	Structured exercise training AND cerebrovascular function AND adult or aging-related populations	Publication date: 2010–2026; Language: English; Species: Humans	4,123	2026-05-04	("Exercise Therapy"[Mesh] OR "Exercise"[Mesh] OR "Resistance Training"[Mesh] OR "High-Intensity Interval Training"[Mesh] OR "Tai Ji"[Mesh] OR "Yoga"[Mesh] OR "exercise training"[Title/Abstract] OR "aerobic training"[Title/Abstract] OR "aerobic exercise"[Title/Abstract] OR "resistance training"[Title/Abstract] OR "strength training"[Title/Abstract] OR "high-intensity interval training"[Title/Abstract] OR HIIT[Title/Abstract] OR "combined training"[Title/Abstract] OR "multimodal exercise"[Title/Abstract] OR "multicomponent exercise"[Title/Abstract] OR "tai chi"[Title/Abstract] OR yoga[Title/Abstract] OR qigong[Title/Abstract]) AND ("Cerebrovascular Circulation"[Mesh] OR "Regional Blood Flow"[Mesh] OR "Blood Flow Velocity"[Mesh] OR "cerebral blood flow"[Title/Abstract] OR "brain blood flow"[Title/Abstract] OR "cerebral perfusion"[Title/Abstract] OR "brain perfusion"[Title/Abstract] OR "cerebral blood velocity"[Title/Abstract] OR "middle cerebral artery velocity"[Title/Abstract] OR "cerebrovascular reactivity"[Title/Abstract] OR "cerebral oxygenation"[Title/Abstract] OR "cerebral hemodynamics"[Title/Abstract] OR "cerebral autoregulation"[Title/Abstract] OR "neurovascular coupling"[Title/Abstract]) AND ("Adult"[Mesh] OR "Aged"[Mesh] OR adult*[Title/Abstract] OR "older adult*"[Title/Abstract] OR aging[Title/Abstract] OR ageing[Title/Abstract] OR elderly[Title/Abstract] OR "mild cognitive

Database	Path	Search Purpose	Limits / Filters	Records Retrieved	Date Searched	Search Syntax
						impairment"[Title/Abstract] OR dementia[Title/Abstract] OR "Alzheimer disease"[Title/Abstract] OR "brain health"[Title/Abstract])
PubMed/MEDLINE	Path B	Structured exercise training AND cognitive outcomes AND dementia, brain health, or aging-related contexts	Publication date: 2010–2026; Language: English; Species: Humans	8,801	2026-05-04	("Exercise Therapy"[Mesh] OR "Exercise"[Mesh] OR "Resistance Training"[Mesh] OR "High-Intensity Interval Training"[Mesh] OR "Tai Ji"[Mesh] OR "Yoga"[Mesh] OR "exercise training"[Title/Abstract] OR "aerobic training"[Title/Abstract] OR "aerobic exercise"[Title/Abstract] OR "resistance training"[Title/Abstract] OR "strength training"[Title/Abstract] OR "high-intensity interval training"[Title/Abstract] OR HIIT[Title/Abstract] OR "combined training"[Title/Abstract] OR "multimodal exercise"[Title/Abstract] OR "multicomponent exercise"[Title/Abstract] OR "tai chi"[Title/Abstract] OR yoga[Title/Abstract] OR qigong[Title/Abstract]) AND ("Cognition"[Mesh] OR "Cognitive Dysfunction"[Mesh] OR "Dementia"[Mesh] OR "Alzheimer Disease"[Mesh] OR cognition[Title/Abstract] OR "cognitive function"[Title/Abstract] OR "cognitive performance"[Title/Abstract] OR "executive function"[Title/Abstract] OR memory[Title/Abstract] OR attention[Title/Abstract] OR "processing speed"[Title/Abstract] OR "working memory"[Title/Abstract] OR "cognitive decline"[Title/Abstract] OR "cognitive impairment"[Title/Abstract] OR "mild cognitive impairment"[Title/Abstract] OR dementia[Title/Abstract] OR "Alzheimer disease"[Title/Abstract] OR "brain aging"[Title/Abstract] OR "brain ageing"[Title/Abstract] OR "brain health"[Title/Abstract]) AND ("mild cognitive impairment"[Title/Abstract] OR dementia[Title/Abstract] OR "Alzheimer disease"[Title/Abstract] OR "cognitive decline"[Title/Abstract] OR "cognitive impairment"[Title/Abstract] OR "brain aging"[Title/Abstract] OR "brain ageing"[Title/Abstract] OR "brain health"[Title/Abstract] OR "older adult*"[Title/Abstract] OR aging[Title/Abstract] OR ageing[Title/Abstract])
Scopus	Path A	Structured exercise training AND cerebrovascular function	Publication year: 2010–2026; Language:	11,310	2026-05-04	TITLE-ABS-KEY ("exercise training" OR "aerobic training" OR "aerobic exercise" OR "resistance training" OR "strength training" OR "high-intensity interval training" OR HIIT OR "combined

Database	Path	Search Purpose	Limits / Filters	Records Retrieved	Date Searched	Search Syntax
		AND adult or aging-related populations	English			training" OR "multimodal exercise" OR "multicomponent exercise" OR "tai chi" OR yoga OR qigong) AND TITLE-ABS-KEY ("cerebral blood flow" OR "brain blood flow" OR "cerebral perfusion" OR "brain perfusion" OR "cerebral blood velocity" OR "middle cerebral artery velocity" OR "cerebrovascular reactivity" OR "cerebral oxygenation" OR "cerebral hemodynamics" OR "cerebral autoregulation" OR "neurovascular coupling") AND TITLE-ABS-KEY (adult* OR "older adult*" OR aging OR ageing OR elderly OR "mild cognitive impairment" OR dementia OR "Alzheimer disease" OR "brain health") AND PUBYEAR > 2009 AND PUBYEAR < 2027 AND (LIMIT-TO (LANGUAGE, "English"))
Scopus	Path B	Structured exercise training AND cognitive outcomes AND dementia, brain health, or aging-related contexts	Publication year: 2010–2026; Language: English	15,879	2026-05-04	TITLE-ABS-KEY ("exercise training" OR "aerobic training" OR "aerobic exercise" OR "resistance training" OR "strength training" OR "high-intensity interval training" OR HIIT OR "combined training" OR "multimodal exercise" OR "multicomponent exercise" OR "tai chi" OR yoga OR qigong) AND TITLE-ABS-KEY (cognition OR "cognitive function" OR "cognitive performance" OR "executive function" OR memory OR attention OR "processing speed" OR "working memory" OR "cognitive decline" OR "cognitive impairment" OR "mild cognitive impairment" OR dementia OR "Alzheimer disease" OR "brain aging" OR "brain ageing" OR "brain health") AND TITLE-ABS-KEY ("mild cognitive impairment" OR dementia OR "Alzheimer disease" OR "cognitive decline" OR "cognitive impairment" OR "brain aging" OR "brain ageing" OR "brain health" OR "older adult*" OR aging OR ageing) AND PUBYEAR > 2009 AND PUBYEAR < 2027 AND (LIMIT-TO (LANGUAGE, "English"))

Table note: PubMed/MEDLINE searches used Medical Subject Headings and title/abstract terms. Scopus searches used TITLE-ABS-KEY syntax. Search filters and record counts are reported as applied on 4 May 2026. Search Path A targeted structured exercise training, cerebrovascular function, and adult or aging-related populations. Search Path B targeted structured exercise training, cognitive outcomes, and dementia, brain health, or aging-related contexts.
