# Supplementary material for: Exercise Training for Cerebrovascular and Cognitive Health in Adults at Risk of Cognitive Decline: A Scoping Review of Healthcare Translation and Evidence Gaps
Source: Healthcare (Basel). 2026 Jun 19;14(12):1774. doi: 10.3390/healthcare14121774 (PMC13299165; doi:10.3390/healthcare14121774)
Supplement: Supplementary file 1 [file healthcare-14-01774-s001.zip › Supplementary Table S2_Search Log.pdf]

Supplementary Table S2. Search log

Manuscript: Exercise Training for Cerebrovascular and Cognitive Health in Adults at Risk of Cognitive Decline: A Scoping Review and Evidence Map

This table documents the search log for the database searches and the PRISMA-ScR record counts used to construct the flow diagram.

Database	Platform	Path	Date searched	Filters	Records retrieved	Export file	Notes
PubMed/ MEDLINE	NCBI	Path A	2026-05-04	2010–2026; English; Humans	4,123	Life_CerebrovascularCognition_Zotero_PubMed_PathA_20260504.ris	Exported as RIS and imported into Zotero.
PubMed/ MEDLINE	NCBI	Path B	2026-05-04	2010–2026; English; Humans	8,801	Life_CerebrovascularCognition_Zotero_PubMed_PathB_20260504.ris	Exported as RIS and imported into Zotero.
Scopus	Elsevier	Path A	2026-05-04	2010–2026; English	11,310	Life_CerebrovascularCognition_Zotero_Scopus_Combined_PathAB_20260504.ris	Scopus Path A and Path B records were exported and managed using a combined Path A/B export workflow before deduplication.
Scopus	Elsevier	Path B	2026-05-04	2010–2026; English	15,879	Life_CerebrovascularCognition_Zotero_Scopus_Combined_PathAB_20260504.ris	Scopus Path A and Path B records were exported and managed using a combined Path A/B export workflow before deduplication.

PRISMA-ScR record counts used for Figure 2

Stage	Count
Records identified from PubMed/MEDLINE Path A	4,123
Records identified from PubMed/MEDLINE Path B	8,801
Records identified from Scopus Path A	11,310
Records identified from Scopus Path B	15,879

Stage	Count
Total records identified through database searching	40,113
Records imported into screening workflow	40,114
Duplicate records removed	10,030
Records after deduplication	30,084
Records excluded by rule-based prescreening	28,874
Records retained for title and abstract screening	1,210
Records excluded after title and abstract screening	1,034
Reports sought for retrieval	176
Reports not retrieved	6
Reports assessed for eligibility	170
Reports excluded after full-text assessment	116
Studies included in the scoping review and evidence map	54

Table note: Search Path A targeted structured exercise training, cerebrovascular function, and adult or aging-related populations. Search Path B targeted structured exercise training, cognitive outcomes, and dementia, brain health, or aging-related contexts. The one-record discrepancy between total database records and imported records was identified during reference management and resolved during deduplication, consistent with the manuscript Methods section and Figure 2.
