# Supplementary material for: Exercise Training for Cerebrovascular and Cognitive Health in Adults at Risk of Cognitive Decline: A Scoping Review of Healthcare Translation and Evidence Gaps
Source: Healthcare (Basel). 2026 Jun 19;14(12):1774. doi: 10.3390/healthcare14121774 (PMC13299165; doi:10.3390/healthcare14121774)
Supplement: Supplementary file 1 [file healthcare-14-01774-s001.zip › Supplementary Table S3_Detailed PCC Framework.pdf]

Supplementary Table S3. Detailed PCC Framework, Eligibility Criteria, Screening Notes, and Prescreening Logic

This supplementary table provides the detailed Population, Concept, and Context (PCC) eligibility framework and the rule-based prescreening logic used before title and abstract screening. The concise PCC framework is summarized in the main manuscript Table 1.

A. Detailed PCC framework and eligibility criteria

PCC Component	Inclusion Criteria	Exclusion Criteria	Examples	Screening Notes
Population	Adults aged 18 years or older, with emphasis on middle-aged adults, older adults, sedentary or physically inactive adults, and adults at elevated risk of cognitive decline.	Children or adolescents only; animal or cell models; exclusively elite athletic populations without relevance to cerebrovascular or cognitive health.	Healthy older adults; sedentary adults; adults with mild cognitive impairment; subjective cognitive decline; hypertension; obesity; type 2 diabetes; metabolic syndrome; cardiovascular risk factors.	Include when the population is adult-based and relevant to cognitive aging, vascular risk, or brain health. If age or risk status is unclear from the abstract, retain for full-text review.
Population risk profile	Adults with vascular, cardiometabolic, functional, or cognitive risk factors relevant to cognitive decline or cerebrovascular dysfunction.	Populations with conditions unrelated to cerebrovascular or cognitive health unless the study directly reports eligible outcomes.	Mild cognitive impairment; subjective cognitive decline; hypertension; obesity; type 2 diabetes; metabolic syndrome; cardiovascular disease risk; sedentary behavior; frailty; cognitive frailty.	Prioritize populations at elevated risk of cognitive decline, but do not exclude healthy older adults if cerebrovascular or cognitive outcomes are assessed.
Concept: structured exercise training	Repeated structured exercise training programs designed to induce physiological, functional, cerebrovascular, or cognitive adaptations.	Acute single-session exercise only; observational physical activity studies; sedentary behavior studies without exercise intervention; diet-only, drug-only, or cognitive-training-only interventions.	Aerobic training; resistance training; combined training; high-intensity interval training; multimodal exercise; tai chi; yoga; qigong; dual-task exercise; coordinative or balance training.	Include only repeated training interventions. If the abstract does not clearly distinguish acute exercise from training, retain for full-text review.
Exercise modality	Aerobic, resistance, combined aerobic and resistance, high-intensity interval, multimodal or multicomponent, mind-body, dual-task, coordinative, or balance-oriented exercise programs.	Interventions in which exercise is not a primary component or cannot be separated from other interventions.	Walking; treadmill; cycling; strength training; elastic band training; HIIT; multicomponent exercise; tai chi; yoga; qigong; exergaming; dual-task training.	Code the dominant modality. If multiple exercise components are delivered, classify as combined or multimodal according to intervention structure.
Context	Community, laboratory, university, outpatient, rehabilitation, health promotion, and aging-related prevention settings.	Inpatient acute care, surgical-only, pharmacological-only, or non-exercise clinical settings unless a structured exercise training intervention is tested.	Community exercise program; supervised laboratory intervention; outpatient rehabilitation; university-based training program; home-based exercise with supervision or monitoring.	Include both supervised and home-based interventions if the exercise prescription is structured and repeated.

PCC Component	Inclusion Criteria	Exclusion Criteria	Examples	Screening Notes
Cerebrovascular outcomes	Studies reporting at least one cerebrovascular or vascular outcome relevant to brain health.	Studies reporting only peripheral fitness or general cardiovascular outcomes without cerebrovascular, vascular, or brain-health relevance.	Cerebral blood flow; cerebral perfusion; cerebral blood velocity; middle cerebral artery velocity; cerebrovascular reactivity; cerebral oxygenation; neurovascular coupling; endothelial function; arterial stiffness; blood pressure-related vascular indicators.	Peripheral vascular outcomes may be retained if explicitly linked to brain health, cognitive aging, or vascular cognitive risk.
Cognitive outcomes	Studies reporting at least one cognitive outcome or dementia-related cognitive screening measure.	Studies without cognitive, neuropsychological, or dementia-related outcomes.	Global cognition; executive function; memory; attention; processing speed; working memory; inhibitory control; cognitive flexibility; verbal fluency; MMSE; MoCA.	Include domain-specific cognitive tests and global screening measures. If cognitive outcomes are mentioned but not specified in the abstract, retain for full-text review.
Outcome integration	Studies reporting cerebrovascular outcomes only, cognitive outcomes only, or both cerebrovascular and cognitive outcomes.	Studies reporting neither cerebrovascular nor cognitive outcomes.	Cerebrovascular only: cerebral blood flow or cerebrovascular reactivity. Cognitive only: MoCA, MMSE, executive function. Both: cerebral perfusion plus cognitive testing.	Studies reporting both domains should be highlighted because they provide the strongest evidence for linking vascular adaptation with cognitive health.
Study design	Original human intervention studies, including randomized controlled trials, non-randomized controlled trials, quasi-experimental studies, controlled pre-post studies, pilot trials, feasibility trials, and single-arm intervention studies.	Reviews, systematic reviews, meta-analyses, editorials, commentaries, letters, protocols without results, conference abstracts without full-text data, case reports, and cross-sectional studies without training intervention.	Randomized exercise trial; controlled training intervention; pre-post supervised exercise program; pilot exercise intervention; feasibility trial.	At title and abstract screening, retain uncertain intervention studies for full-text review. Exclude clearly non-intervention designs.
Publication characteristics	Peer-reviewed full-text articles published in English between January 2010 and 4 May 2026.	Non-English articles; unavailable full texts; grey literature; theses; dissertations; conference-only records without full data.	Peer-reviewed journal articles indexed in PubMed/MEDLINE or Scopus.	Publication restrictions should be reported transparently as limitations.

Note: HIIT = high-intensity interval training; MCI = mild cognitive impairment; MMSE = Mini-Mental State Examination; MoCA = Montreal Cognitive Assessment; PCC = Population, Concept, and Context. Studies were eligible if they reported cerebrovascular outcomes, cognitive outcomes, or both. Studies assessing both cerebrovascular and cognitive outcomes were highlighted because they provide the most direct evidence for evaluating whether exercise-induced vascular adaptations are linked to cognitive health.

B. PCC-based prescreening logic

A rule-based prescreening step was applied after deduplication to improve feasibility while maintaining alignment with the PCC framework. This step was used only to remove records that were clearly unrelated to the review question. Records with uncertain eligibility were retained for title and abstract screening.

Prescreening Decision	Operational Rule	Examples / Notes
Retain for title and abstract screening	Retain records when the title or abstract indicated an adult or aging-related population, a structured exercise or training intervention, and a cerebrovascular, vascular-risk, cognitive, dementia-related, or brain-health outcome.	Examples included exercise training in older adults, mild cognitive impairment, subjective cognitive decline, sedentary adults, frailty, hypertension, obesity, diabetes, cardiovascular risk, or studies reporting cognition, cerebral blood flow, perfusion, cerebrovascular reactivity, cerebral oxygenation, vascular function, or brain-health outcomes.
Retain as uncertain	Retain records when eligibility could not be determined from the title or abstract, including unclear age group, unclear distinction between acute exercise and training, unclear intervention structure, or unclear outcome relevance.	Uncertain records were advanced to title and abstract screening rather than excluded during prescreening. This rule was used to reduce inappropriate exclusion.
Exclude as clearly outside PCC framework	Exclude records clearly unrelated to adult structured exercise training and eligible cerebrovascular or cognitive outcomes.	Examples included non-human studies, pediatric-only studies, non-intervention designs, acute single-session exercise only, reviews, protocols, editorials, conference-only records, studies without eligible exercise components, and studies without eligible cerebrovascular, vascular, brain-related, or cognitive outcomes.
Exclude peripheral-only outcomes without brain-health relevance	Exclude studies reporting only peripheral fitness or general cardiovascular outcomes when no cerebrovascular, vascular-cognitive, brain-health, or cognitive relevance was indicated.	Peripheral vascular or blood pressure outcomes could be retained only when explicitly linked to brain health, cognitive aging, dementia risk, or vascular cognitive risk.
Full-text review decision	Full texts were retrieved for records marked include or maybe after title and abstract screening. Full-text exclusion reasons were recorded using standardized categories.	Reasons included no structured exercise intervention, acute single-session exercise only, wrong population, no eligible outcome, observational design without training, review/editorial/protocol/conference-only record, insufficient information, duplicate dataset, or secondary report.

Note: The prescreening logic was intended to improve workflow feasibility after deduplication and was not used to determine final eligibility. Final inclusion was based on title and abstract screening followed by full-text assessment using the predefined PCC criteria.
