# Supplementary material for: Exercise Training for Cerebrovascular and Cognitive Health in Adults at Risk of Cognitive Decline: A Scoping Review of Healthcare Translation and Evidence Gaps
Source: Healthcare (Basel). 2026 Jun 19;14(12):1774. doi: 10.3390/healthcare14121774 (PMC13299165; doi:10.3390/healthcare14121774)
Supplement: Supplementary file 1 [file healthcare-14-01774-s001.zip › Supplementary Table S4_Full Text Exclusion Reasons.pdf]

Supplementary Table S4. Full-text exclusion reasons after eligibility assessment

This table summarizes the standardized reasons used to exclude reports during full-text eligibility assessment. Counts correspond to the 116 reports excluded after full-text assessment in the PRISMA-ScR flow diagram.

Full-text Exclusion Reason	Operational Definition / Decision Rule	Examples of Excluded Records	Number of Reports Excluded
No eligible cerebrovascular or cognitive outcome	The report did not include any eligible cerebrovascular, vascular-risk, cognitive, brain-health, or dementia-related outcome relevant to the review question.	Exercise intervention studies reporting only unrelated physiological, musculoskeletal, quality-of-life, or peripheral outcomes without eligible cerebrovascular or cognitive endpoints.	34
Not a structured exercise training intervention	The report did not involve a repeated, planned, or structured exercise training program.	General lifestyle advice without a defined exercise component, health education-only interventions, passive modalities, or interventions in which exercise was not a central component.	23
Wrong population	The participant population was outside the PCC framework.	Children or adolescents only, animal or cell studies, exclusively elite athletic populations without brain-health relevance, or populations not relevant to adult cognitive or cerebrovascular health.	17
Observational design without exercise training intervention	The report used an observational, cross-sectional, cohort, or association design and did not test a structured exercise training intervention.	Habitual physical activity studies, sedentary behavior studies, epidemiological analyses, or baseline associations without intervention data.	16
Acute single-session exercise only	The report examined only immediate responses to a single exercise bout and did not include repeated training.	Acute exercise challenge studies, single-session cerebral blood flow or cognitive testing, or immediate post-exercise physiological response studies.	11
Review, protocol, editorial, or conference abstract only	The report did not provide original full-text intervention outcome data.	Narrative reviews, systematic reviews, meta-analyses, protocols, editorials, letters, commentaries, and conference abstracts without full-text data.	7
Insufficient intervention or outcome information	The report lacked sufficient details to determine intervention eligibility, outcome eligibility, or extractable study-level information.	Reports with unclear exercise prescription, unclear training duration, unavailable outcome data, or insufficient description of the cerebrovascular or cognitive outcome.	5
Duplicate dataset or secondary report	The report represented a duplicate publication, overlapping dataset, or secondary analysis that did not contribute distinct eligible intervention-outcome information for the evidence map.	Secondary analyses or duplicate reports of an already included intervention cohort without unique eligible data.	3

Full-text Exclusion Reason	Operational Definition / Decision Rule	Examples of Excluded Records	Number of Reports Excluded
Total reports excluded after full-text assessment	Sum of all predefined full-text exclusion categories.	Not applicable.	116

Table note: Full-text eligibility was assessed using the predefined PCC-based inclusion and exclusion criteria. Each excluded report was assigned one primary exclusion reason to avoid double counting. Reports were excluded only after full-text assessment when they failed to meet criteria related to adult or aging-related populations, structured exercise training, eligible cerebrovascular or cognitive outcomes, original intervention data, or sufficient extractable information. The total number of full-text exclusions was 116 reports, consistent with Figure 2.
