# Supplementary material for: Exercise Training for Cerebrovascular and Cognitive Health in Adults at Risk of Cognitive Decline: A Scoping Review of Healthcare Translation and Evidence Gaps
Source: Healthcare (Basel). 2026 Jun 19;14(12):1774. doi: 10.3390/healthcare14121774 (PMC13299165; doi:10.3390/healthcare14121774)
Supplement: Supplementary file 1 [file healthcare-14-01774-s001.zip › Supplementary Table S5_Data charting framework.pdf]

Supplementary Table S5. Data charting framework for included studies

Charting Domain	Variables Extracted	Operational Definition / Extraction Rule	Use in Synthesis or Evidence Map
Study identification	First author; publication year; article title; journal; country or region; DOI; study record number.	Extract bibliographic information from the final included full-text article and assign each study a unique study number used across the main text and supplementary materials.	Supports traceability between the included study list, supplementary tables, evidence map coding, and reference list.
Study design	Randomized controlled trial; pilot randomized trial; non-randomized controlled trial; quasi-experimental study; controlled pre-post study; single-arm intervention; feasibility study; rehabilitation-based intervention.	Classify study design based on the methods section of each full-text article. When terminology differed across studies, classify according to actual design characteristics rather than title wording alone.	Used to describe methodological characteristics and to support methodological strength mapping.
Participant characteristics	Sample size; age; sex distribution; baseline cognitive status; health status; inclusion criteria; exclusion criteria.	Extract baseline participant characteristics from the methods and baseline tables. When several analysis samples were reported, prioritize the sample used for cerebrovascular, brain-related, or cognitive outcomes.	Supports population description and interpretation of generalizability.
Population risk profile	Healthy adults; healthy older adults; sedentary or inactive adults; mild cognitive impairment; subjective cognitive decline or memory complaint; cognitive frailty or frailty risk; cardiometabolic or vascular risk.	Assign the primary population category according to diagnosis, recruitment criteria, baseline risk profile, or trial objective. Multiple risk descriptors may be noted when applicable.	Used in descriptive synthesis and evidence map subgroup interpretation.
Recruitment and setting	Community; university or laboratory; outpatient clinic;	Extract setting from the methods section. If multiple	Supports assessment of ecological

Charting Domain	Variables Extracted	Operational Definition / Extraction Rule	Use in Synthesis or Evidence Map
	rehabilitation setting; health promotion program; day-care or community center; home-based program.	settings were used, record the main delivery setting and note secondary settings.	relevance, supervision, feasibility, and healthcare translation.
Exercise modality	Aerobic training; resistance training; combined aerobic and resistance training; high-intensity interval training; multimodal or multicomponent exercise; mind-body exercise; dual-task, coordinative, or exergaming exercise; rehabilitation-based exercise.	Code the primary exercise modality according to the dominant intervention component. Multicomponent programs were coded separately when they combined several exercise domains rather than only aerobic plus resistance training. Exercise-based lifestyle and rehabilitation programs were retained when structured exercise was a central intervention component.	Used as the primary exercise modality axis for evidence mapping.
Exercise prescription	Frequency; intensity; time or session duration; type; program duration; progression; supervision; delivery mode; home-based or center-based format.	Extract exercise prescription using the FITT principle and additional implementation details. Intensity was extracted as reported, including heart rate, oxygen uptake, workload, rating of perceived exertion, repetition maximum, or qualitative intensity descriptors.	Supports intervention characterization, dose interpretation, and exercise dose reporting quality mapping.
Comparator or control condition	Usual care; health education; stretching; social activity; cognitive training; waitlist control; active control; no-	Extract comparator details from the trial design and intervention descriptions. When multiple comparison groups were present, record	Supports interpretation of intervention context and methodological strength.

Charting Domain	Variables Extracted	Operational Definition / Extraction Rule	Use in Synthesis or Evidence Map
	treatment control; alternative exercise comparator.	the comparator most relevant to the exercise contrast.	
Adherence and attendance	Attendance rate; session completion; adherence to prescribed intensity or dose; dropout; retention; compliance monitoring; reasons for withdrawal.	Extract adherence and retention data when reported. If adherence was not reported, record as not reported rather than assuming nonadherence.	Supports methodological quality mapping, feasibility interpretation, and healthcare-translation assessment.
Adverse events and safety	Adverse events; serious adverse events; exercise-related injuries; safety monitoring; intervention modification for safety.	Extract safety information from methods, results, and adverse event sections. Record not reported when no safety information was provided.	Supports methodological gaps assessment and clinical translation.
Originally reported cerebrovascular or brain-related outcomes	Cerebral blood flow; brain perfusion; cerebral blood velocity; middle cerebral artery velocity; cerebrovascular reactivity; cerebral oxygenation; cerebral hemodynamics; cerebrovascular impedance; neurovascular coupling; brain activation; brain structure; hippocampal volume; cortical thickness; vascular stiffness; blood pressure-related vascular indicators; endothelial or microvascular function.	Extract cerebrovascular, vascular, or brain-related outcomes using the terminology reported in each original study. Peripheral vascular outcomes were charted only when they were interpreted as relevant to vascular risk, brain health, or cognitive decline. Original outcome labels were retained to preserve traceability.	Supports descriptive synthesis, original outcome traceability, and mapping to grouped cerebrovascular or brain-related domains for Figure 3a.
Grouped cerebrovascular or brain-related	Brain structure or other brain-related surrogate outcomes; cerebral blood flow or perfusion; cerebrovascular	Assign each originally reported cerebrovascular, vascular, or brain-related outcome to a broader grouped	Used for Panel A of Figure 3, which summarizes exercise modalities by

Charting Domain	Variables Extracted	Operational Definition / Extraction Rule	Use in Synthesis or Evidence Map
domain for Figure 3a	reactivity or hemodynamics; cerebral oxygenation; vascular function, arterial stiffness, or blood pressure-related indicators; neurovascular coupling or brain activation.	domain for evidence-map visualization. Closely related labels were grouped to improve interpretability and reduce excessive fragmentation. When no eligible cerebrovascular or brain-related outcome was reported, code as not applicable.	grouped cerebrovascular and brain-related outcome domains.
Cerebrovascular or brain-related assessment methods	Arterial spin labeling MRI; functional MRI; SPECT or nuclear medicine imaging; transcranial Doppler ultrasound; near-infrared spectroscopy; CO_2 challenge; breath-holding task; vascular stiffness or blood pressure assessment; endothelial function testing; brain structural or functional imaging methods.	Extract measurement modality and testing condition. When a study used multiple methods, chart each method linked to its original outcome label and grouped outcome domain.	Supports methodological interpretation of heterogeneity across cerebrovascular and brain-related outcomes.
Originally reported cognitive outcomes	Global cognition; cognitive function; neurocognitive function; executive function; memory; attention; processing speed; working memory; inhibitory control; cognitive flexibility; verbal fluency; cognitive-motor performance; functional cognition; mobility-related cognition; dementia-related screening outcomes; cognitive decline prevention; cognition-related risk;	Extract cognitive outcomes and test names using the terminology reported in each original study. Original outcome labels and assessment tools were retained when available to preserve traceability.	Supports descriptive synthesis, original outcome traceability, and mapping to grouped cognitive domains for Figure 3b.

Charting Domain	Variables Extracted	Operational Definition / Extraction Rule	Use in Synthesis or Evidence Map
	biomarker-linked cognitive outcomes.		
Grouped cognitive domain for Figure 3b	Global cognition; executive function; memory; attention or processing speed; cognitive-motor or functional cognition; dementia-related screening or decline prevention; biomarker-linked or brain-health-related cognitive outcomes.	Assign each originally reported cognitive outcome to a broader grouped cognitive domain for evidence-map visualization. Closely related labels were grouped to improve interpretability and reduce excessive fragmentation. When no eligible cognitive outcome was reported, code as not applicable.	Used for Panel B of Figure 3, which summarizes exercise modalities by grouped cognitive outcome domains.
Cognitive assessment tools	MMSE; MoCA; ADAS-Cog; Trail Making Test; Stroop test; Digit Span; verbal fluency tests; memory recall tests; computerized cognitive batteries; dual-task or functional cognitive assessments.	Extract assessment tools from methods and outcome sections. When tools measured multiple domains, assign to the most relevant domain based on the authors' description or standard neuropsychological interpretation.	Supports interpretation of heterogeneity in cognitive outcome measurement.
Biological or mechanistic markers	Cardiorespiratory fitness; muscular strength; blood pressure; arterial stiffness; metabolic markers; inflammatory markers; oxidative stress markers; neurotrophic factors such as BDNF; brain structural or functional imaging markers.	Extract mechanistic markers when reported as outcomes or mediators. Do not infer mechanisms when not measured.	Supports discussion of mechanistic integration and future research priorities.
Timing of assessment	Baseline; post-intervention; follow-up time points; midpoint assessments; acute	Extract timing of outcome assessment relative to the intervention. When follow-up	Supports interpretation of durability and

Charting Domain	Variables Extracted	Operational Definition / Extraction Rule	Use in Synthesis or Evidence Map
	testing embedded within training study.	was present, record follow-up duration separately from intervention duration.	follow-up limitations.
Main findings	Direction and summary of main cerebrovascular, brain-related, cognitive, functional, and mechanistic findings.	Summarize findings descriptively without recalculating effects. Record positive, mixed, null, negative, or unclear direction when relevant for evidence mapping.	Supports descriptive synthesis and direction-of-findings coding.
Outcome-integration category	Cerebrovascular or brain-related outcomes only; cognitive outcomes only; both cerebrovascular or brain-related and cognitive outcomes.	Assign CVO when at least one eligible cerebrovascular, vascular, or brain-related outcome was reported without cognitive testing; COG when at least one cognitive outcome was reported without eligible cerebrovascular, vascular, or brain-related outcomes; BOTH when at least one outcome from each domain was reported within the same intervention design.	Used to quantify outcome integration and identify both-domain studies that most directly assess vascular-cognitive integration.
Methodological reporting features	Randomization; comparator type; allocation procedures; blinding of outcome assessment; attrition; adherence reporting; adverse event reporting; clarity of exercise prescription; objective intensity monitoring; completeness of outcome reporting; feasibility-related information.	Extract selected features to contextualize evidence quality and translational readiness. These features were mapped descriptively and were not used to exclude studies.	Supports methodological quality mapping and identification of future research priorities.

Charting Domain	Variables Extracted	Operational Definition / Extraction Rule	Use in Synthesis or Evidence Map
Methodological strength	Higher; moderate; preliminary.	Classify methodological strength descriptively based on study design, comparator condition, clarity of exercise prescription, adherence reporting, and relevant outcome assessment. These categories were not formal risk-of-bias ratings.	Used to contextualize evidence maturity across exercise modalities and grouped outcome domains.
Exercise dose reporting quality	Complete FITT; partial FITT; limited FITT.	Classify reporting as complete when frequency, intensity, time, type, progression, and supervision were clearly described; partial when key elements were present but incomplete; limited when prescription details were insufficient for replication or interpretation of exercise dose.	Supports interpretation of replicability, implementation readiness, and exercise prescription translation.

Table note: This supplementary table provides the standardized data charting framework used for the 54 studies included in the scoping review and evidence map. Original study-level outcome labels were retained during data charting to preserve traceability, while closely related cerebrovascular, vascular, brain-related, and cognitive outcomes were grouped into broader domains for Figure 3 visualization. The charted information supported descriptive synthesis, evidence mapping, and methodological quality mapping. Abbreviations: ADAS-Cog, Alzheimer's Disease Assessment Scale-Cognitive Subscale; BDNF, brain-derived neurotrophic factor; BOTH, both cerebrovascular or brain-related and cognitive outcomes; COG, cognitive outcomes only; CO_2, carbon dioxide; CVO, cerebrovascular or brain-related outcomes only; FITT, frequency, intensity, time, and type; MMSE, Mini-Mental State Examination; MoCA, Montreal Cognitive Assessment; MRI, magnetic resonance imaging; SPECT, single-photon emission computed tomography.
