# Supplementary material for: Exercise Training for Cerebrovascular and Cognitive Health in Adults at Risk of Cognitive Decline: A Scoping Review of Healthcare Translation and Evidence Gaps
Source: Healthcare (Basel). 2026 Jun 19;14(12):1774. doi: 10.3390/healthcare14121774 (PMC13299165; doi:10.3390/healthcare14121774)
Supplement: Supplementary file 1 [file healthcare-14-01774-s001.zip › Supplementary Table S6_Exercise Intervention Characteristics.pdf]

Supplementary Table S6. Detailed exercise intervention characteristics extracted from included studies

This table summarizes the exercise-intervention characteristics extracted during data charting. It supports Methods Section 2.6, in which exercise characteristics were extracted according to the FITT principle and supplemented by intervention-reporting elements relevant to replication and clinical translation.

Extraction Domain	Variable Extracted	Operational Definition / Coding Instruction	Examples or Allowed Values	Purpose for Synthesis
Exercise modality	Primary exercise modality	Identify the main structured exercise approach used in the intervention. When more than one component was present, classify according to the dominant or intended intervention structure.	Aerobic training; resistance training; combined aerobic and resistance training; high-intensity interval training; multimodal or multicomponent exercise; mind-body exercise; dual-task, coordinative, or exergaming exercise; rehabilitation-based exercise; other structured exercise.	To classify studies by intervention modality and support descriptive synthesis, evidence mapping, and modality-specific interpretation.
Frequency	Training frequency	Extract the planned number of sessions per week or intervention contacts per week.	Sessions/week; supervised sessions/week; home sessions/week.	To describe exercise dose and compare intervention exposure across studies.
Intensity	Exercise intensity prescription	Extract how intensity was prescribed, monitored, or progressed. Record both absolute and relative intensity measures when reported.	%HRmax; %HRR; %VO_2peak; workload; RPE; walking speed; resistance load; repetition maximum; balance task difficulty; individualized progression criteria.	To assess whether intervention dose was clearly reported and clinically replicable.
Time	Session duration	Extract the planned duration of each exercise session and, when available, active exercise time excluding warm-up and cool-down.	Minutes/session; total active training time; warm-up and cool-down duration.	To support FITT-based description of exercise dose.
Type	Exercise type and content	Extract the specific training activities included in the intervention.	Walking; treadmill; cycling; aerobic dance; aquatic treadmill; resistance machines; elastic bands; body-weight exercise; Tai Chi; yoga; dual-task walking; virtual reality or exergaming; cardiac rehabilitation exercise.	To identify the practical content of each intervention and distinguish exercise types within broad modalities.
Program duration	Intervention length	Extract the total duration of the training program.	Weeks; months; total intervention period.	To compare short-term, medium-term, and longer-term training exposure.
Progression	Progression strategy	Extract whether exercise dose or task difficulty was progressed over time and how progression was implemented.	Increased duration; increased intensity; increased resistance; increased cognitive-motor difficulty; individualized progression; no progression reported.	To evaluate whether interventions provided sufficient adaptive stimulus and to support

Extraction Domain	Variable Extracted	Operational Definition / Coding Instruction	Examples or Allowed Values	Purpose for Synthesis
				methodological quality mapping.
Supervision	Supervision format	Extract whether sessions were supervised, partially supervised, remotely supervised, or unsupervised.	Laboratory supervised; clinical supervised; community supervised; home-based; remotely supervised; mixed delivery.	To contextualize adherence, safety, dose control, and real-world applicability.
Delivery setting	Intervention setting	Extract the physical or clinical setting in which the intervention was delivered.	University laboratory; hospital or outpatient clinic; rehabilitation center; community center; day-care program; home-based program; remote delivery.	To support translation and interpretation of feasibility across clinical and community contexts.
Comparator condition	Comparison group or control condition	Extract the comparator used in controlled studies. For single-arm studies, record absence of comparator.	Usual care; waitlist; health education; stretching; sham or low-intensity activity; cognitive training; alternative exercise; non-exercise control; single-arm design.	To contextualize study design and methodological strength.
Adherence	Attendance and adherence reporting	Extract attendance, compliance with prescribed dose, or adherence monitoring when reported.	Attendance rate; completed sessions; adherence percentage; training logs; wearable monitoring; self-report; not reported.	To interpret intervention exposure and translational feasibility.
Dropout / attrition	Dropout and retention	Extract the number or percentage of participants who withdrew, were lost to follow-up, or did not complete the intervention.	Dropout count; attrition percentage; reasons for withdrawal; completion rate.	To support methodological mapping and assess feasibility.
Adverse events	Safety reporting	Extract whether adverse events, injuries, symptoms, falls, cardiovascular events, or other safety concerns were reported.	No adverse events; minor musculoskeletal symptoms; falls; cardiovascular symptoms; serious adverse events; not reported.	To evaluate safety and clinical translation, especially in older or at-risk populations.
Exercise dose reporting quality	Completeness of FITT and additional reporting elements	Code whether the core FITT elements, frequency, intensity, time, and type, and additional reporting elements, including progression and supervision, were adequately reported.	Complete FITT; partial FITT; limited FITT.	To summarize intervention reporting quality and identify replication gaps.
Outcome timing	Assessment timing related to intervention	Extract when post-intervention outcomes were assessed and whether follow-up was included.	Immediate post-intervention; delayed follow-up; multiple assessment time points; no follow-up.	To support interpretation of durability and timing of cerebrovascular or cognitive adaptations.
Intervention complexity	Single versus multicomponent structure	Record whether the intervention was a single-modality exercise program or contained multiple exercise, cognitive,	Exercise-only; exercise plus cognitive training; exercise-based lifestyle program; cardiac rehabilitation; multimodal exercise program.	To distinguish exercise-specific effects from broader lifestyle or rehabilitation effects.

Extraction Domain	Variable Extracted	Operational Definition / Coding Instruction	Examples or Allowed Values	Purpose for Synthesis
		behavioral, dietary, rehabilitation, or lifestyle components.		
Notes for extraction	Reviewer notes and verification needs	Record any uncertainty about classification, dose reporting, comparator, or intervention content requiring verification.	Unclear intensity; mixed modality; lifestyle component present; full text needed for dose details; verify adherence or adverse event reporting.	To improve transparency of data charting and support final eligibility or coding decisions.

Table note: FITT = frequency, intensity, time, and type; HRmax = maximal heart rate; HRR = heart rate reserve; VO_2peak = peak oxygen uptake; RPE = rating of perceived exertion. This supplementary table provides the intervention-characteristic extraction framework used to chart the 54 studies included in the scoping review. The framework was designed to capture exercise dose, delivery features, comparator condition, adherence, attrition, adverse events, and intervention complexity. Progression and supervision were treated as additional intervention-reporting elements relevant to replication and clinical translation. These variables were used to support descriptive synthesis, evidence mapping, and methodological quality mapping.
