# Supplementary material for: Exercise Training for Cerebrovascular and Cognitive Health in Adults at Risk of Cognitive Decline: A Scoping Review of Healthcare Translation and Evidence Gaps
Source: Healthcare (Basel). 2026 Jun 19;14(12):1774. doi: 10.3390/healthcare14121774 (PMC13299165; doi:10.3390/healthcare14121774)
Supplement: Supplementary file 1 [file healthcare-14-01774-s001.zip › Supplementary Table S7_Cerebrovascular brain-related and cognitive outcome information charted.pdf]

Supplementary Table S7. Cerebrovascular, brain-related, and cognitive outcome information charted from included studies

Outcome Category	Originally Reported Outcome or Outcome Label	Common Assessment Tools or Methods	Grouped Domain for Figure 3	Charting Notes and Use in Synthesis
Cerebrovascular or brain-related outcomes	Brain structure, hippocampal volume, cortical thickness, brain vitality, brain-related outcomes, or other brain-health surrogate outcomes not clearly reported as direct vascular measures.	Structural MRI, volumetric MRI, cortical thickness analysis, hippocampal volume assessment, study-specific brain-health indicators, or related imaging-based methods when reported.	Brain structure or other brain-related surrogate outcomes	Charted when studies reported brain-related outcomes relevant to brain health but not clearly classifiable as direct cerebrovascular measures. Used to preserve brain-health-related outcome information and to distinguish structural or surrogate markers from direct vascular outcomes.
Cerebrovascular or brain-related outcomes	Cerebral blood flow, regional cerebral blood flow, resting CBF, task-related CBF, brain perfusion, cerebral perfusion, regional perfusion, or perfusion change after training.	Arterial spin labeling MRI, perfusion MRI, SPECT, PET when reported, transcranial Doppler-derived flow-related measures when used as proxy indicators.	Cerebral blood flow or perfusion	Charted as direct cerebrovascular evidence. Used to identify studies assessing brain perfusion and to populate the cerebral blood flow or perfusion domain in Figure 3a.
Cerebrovascular or brain-related outcomes	Cerebral blood velocity, middle cerebral artery	Transcranial Doppler ultrasound, transcranial color-	Cerebrovascular reactivity or hemodynamics	Charted when studies reported velocity-based

Outcome Category	Originally Reported Outcome or Outcome Label	Common Assessment Tools or Methods	Grouped Domain for Figure 3	Charting Notes and Use in Synthesis
	velocity, resting velocity, velocity responses during physiological or task conditions, or velocity-based cerebrovascular indices.	coded Doppler ultrasound, or related Doppler-based methods.		hemodynamic outcomes rather than direct perfusion measures. These outcomes were grouped with cerebrovascular reactivity or hemodynamics for Figure 3a when they represented flow dynamics, vascular responsiveness, or hemodynamic regulation.
Cerebrovascular or brain-related outcomes	Cerebrovascular reactivity, vasomotor reactivity, CO_2 reactivity, breath-holding response, hypercapnia response, or reactivity to vasodilatory or physiological stimuli.	Transcranial Doppler ultrasound with CO_2 challenge, breath-holding index, MRI-based cerebrovascular reactivity methods, or comparable reactivity protocols when reported.	Cerebrovascular reactivity or hemodynamics	Used to represent vascular responsiveness and capacity to regulate flow in response to metabolic or physiological demand. Grouped under cerebrovascular reactivity or hemodynamics in Figure 3a.
Cerebrovascular or brain-related outcomes	Cerebral hemodynamic response, cerebrovascular impedance, pulsatility,	Transcranial Doppler ultrasound, vascular ultrasound, hemodynamic modeling, cerebral	Cerebrovascular reactivity or hemodynamics	Charted as hemodynamic or flow-resistance outcomes relevant to cerebrovascular

Outcome Category	Originally Reported Outcome or Outcome Label	Common Assessment Tools or Methods	Grouped Domain for Figure 3	Charting Notes and Use in Synthesis
	flow resistance, hemodynamic regulation, vascular load, cerebral autoregulation, or related flow-resistance indicators.	impedance or pulsatility indices, or related physiological methods.		regulation. Grouped with cerebrovascular reactivity or hemodynamics in Figure 3a because these outcomes reflect dynamic vascular or hemodynamic function.
Cerebrovascular or brain-related outcomes	Cerebral oxygenation, oxygenated hemoglobin, deoxygenated hemoglobin, tissue oxygenation index, prefrontal oxygenation, or cerebral oxygenation responses during rest or task conditions.	Near-infrared spectroscopy, functional near-infrared spectroscopy, or related optical measures when reported.	Cerebral oxygenation	Charted as a cerebrovascular or cerebral hemodynamic outcome when linked to brain oxygenation or oxygen delivery. Used to populate the cerebral oxygenation domain in Figure 3a.
Cerebrovascular or brain-related outcomes	Endothelial function, microvascular function, vascular reactivity, flow-mediated dilation, vascular compliance, vascular markers, or nitric oxide-related vascular indicators relevant to vascular risk.	Flow-mediated dilation, microvascular function testing, vascular ultrasound, endothelial function testing, blood-based endothelial markers, or related vascular assessments when reported.	Vascular function, arterial stiffness, or blood pressure-related indicators	Charted when vascular function was plausibly relevant to cerebrovascular or cognitive health even if not a direct brain perfusion measure. Grouped with vascular function, arterial

Outcome Category	Originally Reported Outcome or Outcome Label	Common Assessment Tools or Methods	Grouped Domain for Figure 3	Charting Notes and Use in Synthesis
				stiffness, or blood pressure-related indicators for Figure 3a.
Cerebrovascular or brain-related outcomes	Carotid stiffness, pulse wave velocity, arterial stiffness, blood pressure, ambulatory blood pressure, vascular compliance, vascular-risk indicators, or blood pressure-related vascular outcomes.	Pulse wave velocity, carotid ultrasound, tonometry, brachial blood pressure, ambulatory blood pressure monitoring, vascular stiffness indices, or related clinical vascular assessments.	Vascular function, arterial stiffness, or blood pressure-related indicators	Charted as supporting vascular outcomes relevant to brain health and vascular cognitive risk. Used to populate the vascular function, arterial stiffness, or blood pressure-related domain in Figure 3a.
Cerebrovascular or brain-related outcomes	Neurovascular coupling, task-related hemodynamic response, cortical activation, brain activation during cognitive or motor tasks, or hemodynamic response to neural activity.	Functional MRI, functional near-infrared spectroscopy, task-based neuroimaging, brain activation measures, or related neurovascular coupling methods.	Neurovascular coupling or brain activation	Charted as an integrated vascular-neural outcome when the outcome reflected hemodynamic response to neural activity. Used to populate the neurovascular coupling or brain activation domain in Figure 3a.
Cognitive outcomes	Global cognitive score, global cognition, cognitive function, neurocognitive function, composite	Mini-Mental State Examination, Montreal Cognitive Assessment, ADAS-Cog, global cognitive	Global cognition	Used to summarize broad cognitive outcomes across intervention studies. Global

Outcome Category	Originally Reported Outcome or Outcome Label	Common Assessment Tools or Methods	Grouped Domain for Figure 3	Charting Notes and Use in Synthesis
	cognitive outcome, overall cognitive performance, MMSE, MoCA, ADAS-Cog, or comparable broad cognition outcomes.	batteries, composite z-scores, or study-specific global cognition measures when reported.		screening tools were retained as original measures but grouped under global cognition for Figure 3b.
Cognitive outcomes	Executive function, planning, set-shifting, cognitive control, frontal-executive performance, or comparable executive-function outcomes.	Trail Making Test Part B, Stroop test, verbal fluency tasks when interpreted as executive function, task-switching tests, computerized executive function tasks, or comparable tests.	Executive function	Prioritized because executive function is commonly sensitive to vascular cognitive aging and exercise-related change. Used to populate the executive function domain in Figure 3b.
Cognitive outcomes	Verbal memory, visual memory, episodic memory, delayed recall, recognition, associative memory, memory complaints, or subjective memory-related outcomes.	Word-list learning and recall tests, logical memory tasks, visual reproduction tasks, delayed recall tasks, recognition tasks, computerized memory batteries, or memory-specific questionnaires when reported.	Memory	Charted as a domain-specific cognitive outcome. Used to populate the memory domain in Figure 3b.
Cognitive outcomes	Attention, reaction time, processing speed, psychomotor speed, timed cognitive performance, symbol substitution, or	Trail Making Test Part A, Digit Symbol Substitution Test, reaction time tasks, computerized attention tasks,	Attention or processing speed	Charted when studies reported attention or speed-based outcomes. Attention and processing speed

Outcome Category	Originally Reported Outcome or Outcome Label	Common Assessment Tools or Methods	Grouped Domain for Figure 3	Charting Notes and Use in Synthesis
	comparable speed-based cognitive measures.	computerized processing speed tasks, or timed neuropsychological tests.		were grouped together for Figure 3b to reduce excessive fragmentation.
Cognitive outcomes	Working memory, short-term memory, digit span, spatial span, updating, n-back performance, or comparable working-memory outcomes.	Digit Span forward or backward, spatial span tasks, n-back tasks, computerized working memory tests, or comparable short-term cognitive control measures.	Executive function or attention or processing speed, depending on the authors' interpretation and test context	Working memory outcomes were retained as original labels during charting. For Figure 3b, working memory was grouped into the closest broader cognitive domain, usually executive function or attention or processing speed, according to the study's interpretation and assessment context.
Cognitive outcomes	Inhibitory control, interference control, cognitive flexibility, task switching, set shifting, go/no-go performance, or comparable executive subdomains.	Stroop interference score, go/no-go tasks, task-switching tests, Trail Making Test ratio or difference scores, or computerized inhibition and flexibility tasks.	Executive function	Charted when studies specified executive subdomains beyond global executive function. These outcomes were grouped under executive function for Figure 3b.

Outcome Category	Originally Reported Outcome or Outcome Label	Common Assessment Tools or Methods	Grouped Domain for Figure 3	Charting Notes and Use in Synthesis
Cognitive outcomes	Verbal fluency, semantic fluency, phonemic fluency, language-related cognitive performance, or comparable fluency outcomes.	Verbal fluency tests, semantic category fluency, phonemic fluency tasks, or related neuropsychological measures.	Executive function or global cognition, depending on the authors' interpretation	Verbal fluency was retained as an original outcome label. For Figure 3b, it was grouped into executive function when interpreted as an executive-language task, or global cognition when reported only as part of a broad cognitive battery.
Cognitive outcomes	Dual-task performance, mobility-cognition integration, functional tasks, cognitive-motor performance, everyday functional cognitive outcomes, mobility-related cognition, frailty-related cognition, fall-risk-related cognition, or function-linked cognitive outcomes.	Dual-task walking tests, functional task performance, mobility-cognition outcomes, exergaming assessments, cognitive-motor assessments, functional cognition tasks, or mobility-related cognitive measures.	Cognitive-motor or functional cognition	Used for interventions combining physical and cognitive demands, especially dual-task, exergaming, functional task, rehabilitation-based, and multimodal programs. Used to populate the cognitive-motor or functional cognition domain in Figure 3b.
Cognitive outcomes	Dementia-related screening, cognitive decline prevention, Alzheimer's disease risk-related cognition,	Dementia screening tools, cognitive decline prevention endpoints, Alzheimer's disease	Dementia-related screening or decline prevention	Charted when studies framed cognitive outcomes in relation to dementia

Outcome Category	Originally Reported Outcome or Outcome Label	Common Assessment Tools or Methods	Grouped Domain for Figure 3	Charting Notes and Use in Synthesis
	cognition-related risk, cognitive health risk, or outcomes explicitly framed as dementia prevention or decline mitigation.	risk-related cognitive measures, study-specific dementia prevention outcomes, or cognition-related health benefit indicators.		prevention, Alzheimer's disease risk, or cognitive decline mitigation. Used to populate the dementia-related screening or decline prevention domain in Figure 3b.
Cognitive outcomes	Cognitive outcomes reported together with BDNF, inflammatory markers, immunological outcomes, brain vitality, hippocampal volume, cortical thickness, brain activation, neuropsychological outcomes linked to biomarkers, or brain-health-related cognitive endpoints.	Blood-based biomarker assays, neurotrophic marker assessment, inflammatory or immunological markers, structural or functional neuroimaging, study-specific brain-health outcomes, or cognitive tests interpreted together with biological markers.	Biomarker-linked or brain-health-related cognitive outcomes	Charted when cognitive outcomes were explicitly linked with biomarkers, neurobiological markers, brain structure, brain activation, or broader brain-health indicators. Used to populate the biomarker-linked or brain-health-related cognitive domain in Figure 3b.
Biological and mechanistic markers	BDNF, inflammatory markers, immunological outcomes, metabolic indicators, vascular-risk biomarkers, cardiorespiratory fitness, muscular	Blood-based biomarker assays, cardiorespiratory fitness testing, strength testing, physical performance tests, metabolic testing, vascular-risk	Not a standalone Figure 3 domain unless linked to cerebrovascular, brain-related, or cognitive outcomes	Charted when reported as secondary or mechanistic variables relevant to interpretation of cognitive or vascular

Outcome Category	Originally Reported Outcome or Outcome Label	Common Assessment Tools or Methods	Grouped Domain for Figure 3	Charting Notes and Use in Synthesis
	strength, balance, mobility, physical function, or mechanistic correlates of cognitive or cerebrovascular adaptation.	assessments, or functional performance measures.		adaptation. Mechanisms were not inferred when markers were not measured. When biomarkers were explicitly linked to cognitive or brain-health outcomes, they informed the biomarker-linked or brain-health-related cognitive domain.
Assessment timing	Baseline, post-intervention, follow-up, interim assessment, long-term follow-up, retention assessment, or acute testing embedded within a training study.	Study-specific testing schedules.	Not applicable	Used to distinguish immediate post-training outcomes from longer-term maintenance or follow-up effects.
Outcome interpretation	Whether outcomes favored exercise, were mixed, null, negative, or unclear; whether studies assessed cerebrovascular or brain-related outcomes only, cognitive outcomes only, or both.	Derived from reported study results and coded according to the evidence map framework.	Not applicable	Used to support descriptive synthesis, outcome-integration categories, direction-of-findings coding, and interpretation of the evidence map.

Table note: This supplementary table summarizes the cerebrovascular, brain-related, cognitive, biological, and mechanistic outcome information charted from the included studies. Outcome labels

reported in individual studies were retained during data charting to preserve traceability, and closely related outcomes were grouped into broader domains for Figure 3 visualization. The grouped domains shown in this table were used to support descriptive synthesis, outcome-integration classification, evidence-map construction, and methodological interpretation. Abbreviations: ADAS-Cog, Alzheimer's Disease Assessment Scale-Cognitive Subscale; BDNF, brain-derived neurotrophic factor; CBF, cerebral blood flow; CO_2, carbon dioxide; MMSE, Mini-Mental State Examination; MoCA, Montreal Cognitive Assessment; MRI, magnetic resonance imaging; PET, positron emission tomography; SPECT, single-photon emission computed tomography.
