# Supplementary material for: Exercise Training for Cerebrovascular and Cognitive Health in Adults at Risk of Cognitive Decline: A Scoping Review of Healthcare Translation and Evidence Gaps
Source: Healthcare (Basel). 2026 Jun 19;14(12):1774. doi: 10.3390/healthcare14121774 (PMC13299165; doi:10.3390/healthcare14121774)
Supplement: Supplementary file 1 [file healthcare-14-01774-s001.zip › Supplementary Table S9_Detailed study-level characteristics.pdf]

Supplementary Table S9. Detailed study-level characteristics and evidence-map coding of the 54 included studies

No.	Author, Year	Population / Risk Profile	Exercise Modality	Study Design / Intervention Duration	Main Outcome Domain	Grouped Cerebrovascular or Brain-Related Domain for Figure 3a	Grouped Cognitive Domain for Figure 3b	Outcome Integration
1	Suzuki et al., 2013[14]	Older adults with MCI	Multicomponent exercise	RCT	Cognitive outcomes	Not applicable	Global cognition	Cognitive only
2	Sungkarat et al., 2018[15]	Older adults with MCI	Tai Chi	RCT	Cognition, BDNF	Not applicable	Global cognition; Biomarker-linked or brain-health-related cognitive outcomes	Cognitive only
3	Tomoto et al., 2021[21]	Amnestic MCI	Aerobic exercise	One-year intervention	Carotid stiffness, CBF	Cerebral blood flow or perfusion; Vascular function, arterial stiffness, or BP-related indicators	Not applicable	Cerebrovascular only
4	Tomoto et al., 2021[22]	MCI	Aerobic exercise	One-year intervention	Cerebral vasomotor reactivity	Cerebrovascular reactivity or	Not applicable	Cerebrovascular only

No.	Author, Year	Population / Risk Profile	Exercise Modality	Study Design / Intervention Duration	Main Outcome Domain	Grouped Cerebrovascular or Brain-Related Domain for Figure 3a	Grouped Cognitive Domain for Figure 3b	Outcome Integration
						hemodynamics		
5	Thomas et al., 2020[23]	MCI	Aerobic exercise	12 months	Brain perfusion	Cerebral blood flow or perfusion	Not applicable	Cerebrovascular only
6	Alfini et al., 2019[24]	MCI	Exercise training	Not clearly reported	Resting CBF	Cerebral blood flow or perfusion	Not applicable	Cerebrovascular only
7	Tarumi et al., 2019[25]	Amnestic MCI	Exercise training	One-year RCT	Cognitive and brain-related outcomes	Brain structure or other brain-related surrogate outcomes	Global cognition; Biomarker-linked or brain-health-related cognitive outcomes	Both
8	Bliss et al., 2022[26]	Sedentary obese older adults	Aerobic exercise	Training intervention	Cerebrovascular and cognitive function	Cerebrovascular reactivity or hemodynamics	Global cognition	Both
9	Tarumi et al., 2022[27]	Cognitively normal older adults	Aerobic exercise	One-year RCT	Neurocognitive function	Not applicable	Global cognition	Cognitive only

No.	Author, Year	Population / Risk Profile	Exercise Modality	Study Design / Intervention Duration	Main Outcome Domain	Grouped Cerebrovascular or Brain-Related Domain for Figure 3a	Grouped Cognitive Domain for Figure 3b	Outcome Integration
10	Chapman et al., 2013[28]	Aging adults	Aerobic exercise	Shorter-term intervention	Brain, cognition, fitness	Brain structure or other brain-related surrogate outcomes	Global cognition; Biomarker-linked or brain-health-related cognitive outcomes	Cognitive only
11	Penukonda et al., 2025[29]	Amnestic MCI	Exercise training	One-year intervention	Cognition, fitness, vascular stiffness, CO_2 reactivity	Cerebrovascular reactivity or hemodynamics; Vascular function, arterial stiffness, or BP-related indicators	Global cognition	Both
12	Sugawara et al., 2026[30]	Amnestic MCI	Exercise training	One-year RCT	Cerebrovascular impedance	Cerebrovascular reactivity or hemodynamics	Not applicable	Cerebrovascular only

No.	Author, Year	Population / Risk Profile	Exercise Modality	Study Design / Intervention Duration	Main Outcome Domain	Grouped Cerebrovascular or Brain-Related Domain for Figure 3a	Grouped Cognitive Domain for Figure 3b	Outcome Integration
13	Kunieda et al., 2022[31]	Older adults with amnestic MCI	Dual-task training	Not clearly reported	Regional CBF	Cerebral blood flow or perfusion	Not applicable	Cerebrovascular only
14	Odano et al., 2022[32]	MCI	Whole-body vibration exercise	Training intervention	Regional CBF, cognitive function	Cerebral blood flow or perfusion	Global cognition	Both
15	Boku et al., 2022[33]	MCI	Multicomponent day-care program	Not clearly reported	CBF	Cerebral blood flow or perfusion	Not applicable	Cerebrovascular only
16	So et al., 2024[34]	Healthy older adults	Aquatic treadmill exercise	Moderate-intensity intervention	Cognitive function, CBF	Cerebral blood flow or perfusion	Global cognition	Both
17	Hamasaki et al., 2019[35]	Middle-aged and older adults	Aerobic exercise plus lactotripeptide	Combined nutrition-exercise intervention	Cognitive function, cerebral oxygenation	Cerebral oxygenation	Global cognition	Both
18	Mitchell et al., 2022[36]	Adult clinical or at-risk population	Aerobic exercise	Pilot randomized clinical trial	Cerebral hemodynamics	Cerebrovascular reactivity or hemodynamics	Not applicable	Cerebrovascular only

No.	Author, Year	Population / Risk Profile	Exercise Modality	Study Design / Intervention Duration	Main Outcome Domain	Grouped Cerebrovascular or Brain-Related Domain for Figure 3a	Grouped Cognitive Domain for Figure 3b	Outcome Integration
19	Akazawa et al., 2012[37]	Postmenopausal women	Aerobic exercise	Training intervention	CBF	Cerebral blood flow or perfusion	Not applicable	Cerebrovascular only
20	Zhu et al., 2022[38]	Elderly people with amnestic MCI	Aerobic dance	3-month RCT	Hippocampal volume, cognition	Brain structure or other brain-related surrogate outcomes	Global cognition; Biomarker-linked or brain-health-related cognitive outcomes	Cognitive only
21	Baker et al., 2010[39]	MCI	Aerobic exercise	Controlled trial	Cognitive outcomes	Not applicable	Global cognition	Cognitive only
22	Zhu et al., 2018[40]	MCI	Aerobic dance	Specially designed routine	Cognitive outcomes	Not applicable	Global cognition	Cognitive only
23	Suzuki et al., 2012[41]	Older adults with amnestic MCI	Multicomponent exercise	RCT	Cognitive function	Not applicable	Global cognition	Cognitive only
24	Law et al., 2014[42]	Older adults with cognitive impairme	Functional task exercise	RCT	Cognitive and functional outcomes	Not applicable	Global cognition; Cognitive-motor or	Cognitive only

No.	Author, Year	Population / Risk Profile	Exercise Modality	Study Design / Intervention Duration	Main Outcome Domain	Grouped Cerebrovascular or Brain-Related Domain for Figure 3a	Grouped Cognitive Domain for Figure 3b	Outcome Integration
		nt at risk of Alzheimer's disease					functional cognition	
25	Law et al., 2019[43]	Older adults with MCI	Functional task exercise	Randomized pilot trial	Cognitive function	Not applicable	Global cognition	Cognitive only
26	Li et al., 2022[44]	Persons with MCI	Peer-supported exercise	Waitlist RCT	Cognitive and brain vitality outcomes	Brain structure or other brain-related surrogate outcomes	Global cognition; Biomarker-linked or brain-health-related cognitive outcomes	Cognitive only
27	De Wit et al., 2018[45]	Mild neurocognitive disorder	Physical exercise plus cognitive engagement	Group-randomized pilot trial	Cognitive engagement and cognitive outcomes	Not applicable	Global cognition; Cognitive-motor or functional cognition	Cognitive only
28	Shimada et al., 2018[46]	Older adults	Golf training	RCT	Cognition	Not applicable	Global cognition	Cognitive only
29	Bae et al., 2020[47]	Older adults with	Multicomponent dual-task exercise	RCT	Cortical thickness, cognition	Brain structure or other brain-	Global cognition; Biomarker-linked or	Cognitive only

No.	Author, Year	Population / Risk Profile	Exercise Modality	Study Design / Intervention Duration	Main Outcome Domain	Grouped Cerebrovascular or Brain-Related Domain for Figure 3a	Grouped Cognitive Domain for Figure 3b	Outcome Integration
		cognitive decline				related surrogate outcomes	brain-health-related cognitive outcomes	
30	Thapa et al., 2020[48]	Older adults with MCI	Virtual reality-based exercise	RCT	Cognition	Not applicable	Global cognition	Cognitive only
31	Park et al., 2019[49]	MCI	Physical activity, aerobic exercise, cognitive exercise	Randomized clinical study	Cognitive decline prevention	Not applicable	Dementia-related screening or decline prevention	Cognitive only
32	McEwen et al., 2018[50]	Older adults with subjective memory impairment	Aerobic exercise plus memory training	Simultaneous intervention	Memory, cognition	Not applicable	Memory; Global cognition	Cognitive only
33	Sungkarat et al., 2017[51]	Older adults with MCI	Tai Chi	RCT	Cognition, fall risk	Not applicable	Global cognition; Cognitive-motor or functional cognition	Cognitive only

No.	Author, Year	Population / Risk Profile	Exercise Modality	Study Design / Intervention Duration	Main Outcome Domain	Grouped Cerebrovascular or Brain-Related Domain for Figure 3a	Grouped Cognitive Domain for Figure 3b	Outcome Integration
34	Chen et al., 2023[52]	Adults ≥60 years with T2D and MCI	Tai Chi Chuan	Randomized clinical trial	Cognitive function	Not applicable	Global cognition	Cognitive only
35	Grzenda et al., 2024[53]	Older women at risk for Alzheimer's disease	Yoga	Comparative intervention	Cognitive and immunological outcomes	Not applicable	Global cognition; Biomarker-linked or brain-health-related cognitive outcomes	Cognitive only
36	Chobe et al., 2022[54]	Elderly adults with MCI	Integrated yoga and Ayurveda Rasayana	Non-randomized three-arm clinical trial	Cognitive function	Not applicable	Global cognition	Cognitive only
37	Wu et al., 2025[55]	Older adults with cognitive frailty	Resistance training	RCT	Cognitive and frailty-related outcomes	Not applicable	Global cognition; Cognitive-motor or functional cognition	Cognitive only
38	Yoon et al., 2018[56]	Cognitive frailty	Resistance exercise	RCT	Cognitive function, physical	Not applicable	Global cognition; Cognitive-motor or	Cognitive only

No.	Author, Year	Population / Risk Profile	Exercise Modality	Study Design / Intervention Duration	Main Outcome Domain	Grouped Cerebrovascular or Brain-Related Domain for Figure 3a	Grouped Cognitive Domain for Figure 3b	Outcome Integration
					performance		functional cognition	
39	Mavros et al., 2017[57]	Older adults with MCI	Resistance training	SMART trial-related analysis	Cognitive function, strength gains	Not applicable	Global cognition; Cognitive-motor or functional cognition	Cognitive only
40	Langoni et al., 2019[58]	Older adults with MCI	Exercise training	RCT	Cognition, conditioning, endurance, balance	Not applicable	Global cognition; Cognitive-motor or functional cognition	Cognitive only
41	Kušleikienė et al., 2025[59]	Older adults at low or high risk of MCI	Resistance training	12-week RCT	Cognition, cortical thickness	Brain structure or other brain-related surrogate outcomes	Global cognition; Biomarker-linked or brain-health-related cognitive outcomes	Cognitive only
42	Krootnark et al., 2024[60]	Older persons with MCI	Home-based aerobic versus resistance exercise	RCT	Cognition	Not applicable	Global cognition	Cognitive only

No.	Author, Year	Population / Risk Profile	Exercise Modality	Study Design / Intervention Duration	Main Outcome Domain	Grouped Cerebrovascular or Brain-Related Domain for Figure 3a	Grouped Cognitive Domain for Figure 3b	Outcome Integration
43	Huang et al., 2025[61]	Older adults with MCI	Remotely supervised aerobic and resistance training	Pilot three-arm RCT	Cognitive and feasibility outcomes	Not applicable	Global cognition	Cognitive only
44	Liao et al., 2021[62]	Frail older adults	Exergaming versus combined exercise	RCT	Cognitive function, brain activation	Neurovascular coupling or brain activation	Global cognition; Biomarker-linked or brain-health-related cognitive outcomes	Both
45	Jhaveri et al., 2023[63]	Older adults with MCI	SMARTfit dual-task exercise	Community-based pilot study	Cognition, physical function	Not applicable	Global cognition; Cognitive-motor or functional cognition	Cognitive only
46	Saeed et al., 2024[64]	Adults with MCI	Exergame balance training	Randomized trial	Cognitive function	Not applicable	Global cognition	Cognitive only
47	Shimada et al., 2018[65]	Patients with MCI	Combined physical and cognitive exercise	Randomized clinical trial	Cognition, mobility	Not applicable	Global cognition; Cognitive-motor or	Cognitive only

No.	Author, Year	Population / Risk Profile	Exercise Modality	Study Design / Intervention Duration	Main Outcome Domain	Grouped Cerebrovascular or Brain-Related Domain for Figure 3a	Grouped Cognitive Domain for Figure 3b	Outcome Integration
							functional cognition	
48	Bisbe et al., 2020[66]	Older adults with amnestic MCI	Choreographed exercise versus multimodal physical therapy	Randomized clinical trial	Cognitive outcomes	Not applicable	Global cognition	Cognitive only
49	de Oliveira Silva et al., 2019[67]	Elderly individuals with MCI or Alzheimer's disease	Multimodal training	3-month RCT	Mobility, executive function	Not applicable	Executive function; Cognitive-motor or functional cognition	Cognitive only
50	Jeong et al., 2021[68]	Patients with MCI	Multicomponent intervention	RCT	Habitual physical activity, cognitive function	Not applicable	Global cognition	Cognitive only
51	Cox et al., 2019[69]	Older adults at risk of Alzheimer's disease	Home-based physical activity program	24-month RCT	Health benefits, adherence, cognition-related risk	Not applicable	Dementia-related screening or decline prevention	Cognitive only

No.	Author, Year	Population / Risk Profile	Exercise Modality	Study Design / Intervention Duration	Main Outcome Domain	Grouped Cerebrovascular or Brain-Related Domain for Figure 3a	Grouped Cognitive Domain for Figure 3b	Outcome Integration
52	Smith et al., 2022[70]	Individuals with resistant hypertension	Lifestyle modification including exercise	TRIUMPH trial	Cognitive function	Not applicable	Global cognition	Cognitive only
53	Alosco et al., 2014[71]	Older adults with heart failure	Cardiac rehabilitation	Clinical rehabilitation study	Cognitive function	Not applicable	Global cognition	Cognitive only
54	Stanek et al., 2011[72]	Older adults with cardiovascular disease	Cardiac rehabilitation	Rehabilitation intervention	Cognitive function	Not applicable	Global cognition	Cognitive only

Table note: This table summarizes the study-level characteristics and evidence-map coding of the 54 studies included in the scoping review. Rows are ordered according to the final manuscript reference number, shown in brackets after the year in the Author, Year column. Original study-level outcome labels were retained in the Main Outcome Domain column, and corresponding grouped cerebrovascular or brain-related and cognitive domains were assigned for Figure 3 visualization to improve interpretability and maintain traceability. Outcome-integration categories indicate whether each study reported cerebrovascular or brain-related outcomes only, cognitive outcomes only, or both domains within the same intervention design. BDNF = brain-derived neurotrophic factor; BP = blood pressure; CBF = cerebral blood flow; CO_2 = carbon dioxide; MCI = mild cognitive impairment; RCT = randomized controlled trial; T2D = type 2 diabetes.
