# Supplementary material for: Exercise Training for Cerebrovascular and Cognitive Health in Adults at Risk of Cognitive Decline: A Scoping Review of Healthcare Translation and Evidence Gaps
Source: Healthcare (Basel). 2026 Jun 19;14(12):1774. doi: 10.3390/healthcare14121774 (PMC13299165; doi:10.3390/healthcare14121774)
Supplement: Supplementary file 1 [file healthcare-14-01774-s001.zip › Supplementary_Table_S8_full_coding_framework_with_study_level_coding_revised.pdf]

Supplementary Table S8. Full evidence map coding framework, operational definitions, and decision rules

| Coding Domain | Coding Category | Code | Definition / Coding Rule | Use in Evidence Map |
|---|---|---|---|---|
| Exercise modality | Aerobic training | AER | Structured endurance-based exercise, including walking, treadmill exercise, cycling, aerobic dance, aquatic treadmill exercise, or other repeated endurance-based protocols. | Used as a primary exercise modality category in the evidence map. |
| Exercise modality | Resistance training | RET | Structured muscle-strengthening exercise, including progressive resistance training, free weights, elastic bands, body-weight resistance exercise, or machine-based strength training. | Used to identify strength-focused exercise interventions. |
| Exercise modality | Combined aerobic and resistance training | COM | Programs containing both aerobic and resistance training components as primary intervention elements. | Used to distinguish combined training from single-modality and broader multimodal programs. |
| Exercise modality | High-intensity interval training | HIIT | Repeated high-intensity exercise bouts alternated with recovery intervals. | Used to identify time-efficient high-intensity exercise interventions when reported as the dominant modality. |
| Exercise modality | Multimodal or multicomponent exercise | MME | Programs combining multiple exercise components, such as aerobic exercise, resistance training, balance, | Used to map clinically translatable multicomponent exercise interventions. |

| Coding Domain | Coding Category | Code | Definition / Coding Rule | Use in Evidence Map |
|---|---|---|---|---|
| | | | coordination, flexibility, functional training, or cognitive-motor tasks. | |
| Exercise modality | Mind-body exercise | MBE | Tai Chi, yoga, qigong, or related integrative mind-body programs combining movement with attention, breathing, postural control, or self-regulation. | Used to identify exercise interventions involving both physical movement and attentional regulation. |
| Exercise modality | Dual-task, coordinative, or exergaming exercise | DTE | Exercise emphasizing cognitive-motor challenge, coordinative movement, dual-task performance, virtual reality, or exergaming. | Used to identify interventions targeting motor-cognitive integration. |
| Exercise modality | Rehabilitation-based exercise | REH | Cardiac rehabilitation, clinical rehabilitation, or structured exercise delivered within broader lifestyle or clinical care programs. | Used to identify structured exercise delivered in clinical, rehabilitation, or vascular-risk contexts. |
| Population category | Healthy adults | HA | Adults without major diagnosed cognitive, neurological, vascular, or metabolic risk. | Used to distinguish prevention-focused adult evidence. |
| Population category | Healthy older adults | HOA | Older adults without diagnosed cognitive impairment or major clinical disease relevant to the review question. | Used to identify healthy aging and prevention-oriented evidence. |
| Population category | Sedentary or inactive adults | SED | Adults with low baseline physical activity, sedentary behavior, or physical inactivity as a defining study characteristic. | Used to identify evidence in inactive or insufficiently active populations. |

| Coding Domain | Coding Category | Code | Definition / Coding Rule | Use in Evidence Map |
|---|---|---|---|---|
| Population category | Mild cognitive impairment | MCI | Participants diagnosed with mild cognitive impairment or mild neurocognitive disorder using validated clinical or research criteria. | Used to identify populations at elevated risk of cognitive decline. |
| Population category | Subjective cognitive decline or memory complaint | SCD | Participants reporting subjective cognitive decline, memory complaints, or perceived cognitive difficulty without confirmed clinical impairment. | Used to identify early-risk or preclinical cognitive-risk populations. |
| Population category | Cognitive frailty or frailty risk | CF | Participants with cognitive vulnerability combined with physical frailty, functional limitation, or frailty-related risk. | Used to identify frailty-related cognitive-risk populations. |
| Population category | Cardiometabolic or vascular risk | CMVR | Participants with hypertension, obesity, type 2 diabetes, metabolic syndrome, cardiovascular disease, heart failure, resistant hypertension, or related vascular-risk conditions. | Used to identify populations with vascular or cardiometabolic risk relevant to brain health. |
| Cerebrovascular or brain-related outcome domain | Brain structure or other brain-related surrogate outcomes | BRAIN | Brain structure, hippocampal volume, cortical thickness, brain vitality, brain-related outcomes, or other brain-health surrogate outcomes that were not clearly reported as direct cerebrovascular measures. | Used in Panel A of Figure 3 to map brain-related surrogate outcomes that may inform brain-health mechanisms but are not direct vascular measures. |

| Coding Domain | Coding Category | Code | Definition / Coding Rule | Use in Evidence Map |
|---|---|---|---|---|
| Cerebrovascular or brain-related outcome domain | Cerebral blood flow or perfusion | CBF/PERF | Resting, regional, or task-related cerebral blood flow, brain perfusion, regional CBF, or related flow-based measures assessed using MRI, fMRI, SPECT, Doppler-based methods, or comparable techniques. | Used in Panel A of Figure 3 to map flow- or perfusion-related outcomes. |
| Cerebrovascular or brain-related outcome domain | Cerebrovascular reactivity or hemodynamics | CVR/HEMO | Cerebrovascular reactivity, cerebral vasomotor reactivity, $CO_2$ reactivity, breath-holding response, cerebrovascular impedance, cerebral hemodynamics, cerebral autoregulation, or related hemodynamic responsiveness outcomes. | Used in Panel A of Figure 3 to map vascular responsiveness and hemodynamic outcomes. |
| Cerebrovascular or brain-related outcome domain | Cerebral oxygenation | COX | Cerebral oxygenation, oxygenated hemoglobin, deoxygenated hemoglobin, tissue oxygen saturation, or related NIRS-derived oxygenation measures. | Used in Panel A of Figure 3 to map oxygenation-related cerebrovascular outcomes. |
| Cerebrovascular or brain-related outcome domain | Vascular function, arterial stiffness, or blood pressure-related indicators | VASC/BP | Endothelial function, microvascular function, flow-mediated dilation, vascular function, arterial stiffness, pulse wave velocity, carotid stiffness, blood pressure, ambulatory blood pressure, or other | Used in Panel A of Figure 3 to map systemic or peripheral vascular-risk outcomes relevant to cerebrovascular and cognitive health. |

| Coding Domain | Coding Category | Code | Definition / Coding Rule | Use in Evidence Map |
|---|---|---|---|---|
| | | | vascular-risk indicators relevant to brain health. | |
| Cerebrovascular or brain-related outcome domain | Neurovascular coupling or brain activation | NVC/ACT | Hemodynamic, vascular, or brain activation responses during cognitive, motor, or task-based stimulation, including neurovascular coupling or brain activation outcomes. | Used in Panel A of Figure 3 to identify studies linking vascular responses, neural activity, or task-related activation. |
| Cognitive outcome domain | Global cognition | GCOG | Global cognitive screening measures, cognitive function, neurocognitive function, composite cognitive scores, MMSE, MoCA, ADAS-Cog, or comparable global cognitive outcomes. | Used in Panel B of Figure 3 to map broad cognitive outcomes. |
| Cognitive outcome domain | Executive function | EXF | Executive function, planning, inhibition, set-shifting, cognitive control, Stroop performance, Trail Making Test Part B, or comparable executive-function measures. | Used in Panel B of Figure 3 to map executive-function outcomes. |
| Cognitive outcome domain | Memory | MEM | Verbal memory, visual memory, episodic memory, delayed recall, recognition memory, subjective memory-related outcomes, or comparable memory-specific outcomes. | Used in Panel B of Figure 3 to map memory-specific outcomes. |
| Cognitive outcome domain | Attention or processing speed | ATT/PS | Reaction time, attention tasks, symbol substitution, timed cognitive tasks, | Used in Panel B of Figure 3 to map attention and |

| Coding Domain | Coding Category | Code | Definition / Coding Rule | Use in Evidence Map |
|---|---|---|---|---|
| | | | processing speed, or comparable attention and speed-based cognitive measures. | processing-speed outcomes. |
| Cognitive outcome domain | Cognitive-motor or functional cognition | CMF | Dual-task performance, mobility-cognition integration, functional cognitive tasks, frailty-related cognition, fall-risk-related cognition, physical function linked with cognition, daily function-related cognitive performance, or cognitive-motor outcomes. | Used in Panel B of Figure 3 to map functional cognition and motor-cognitive integration outcomes. |
| Cognitive outcome domain | Dementia-related screening or decline prevention | DEM/PREV | Dementia-related screening, cognitive decline prevention, Alzheimer's disease risk-related cognition, cognition-related risk, or outcomes explicitly framed as dementia prevention or cognitive decline mitigation. | Used in Panel B of Figure 3 to map dementia prevention and cognitive-risk outcomes. |
| Cognitive outcome domain | Biomarker-linked or brain-health-related cognitive outcomes | BIO/BRAIN | Cognitive outcomes reported together with BDNF, immunological markers, brain vitality, cortical thickness, hippocampal volume, brain activation, or other biological or brain-health-related markers. | Used in Panel B of Figure 3 to map cognitive outcomes linked with biomarkers, neurobiological markers, or brain-health indicators. |

| Coding Domain | Coding Category | Code | Definition / Coding Rule | Use in Evidence Map |
|---|---|---|---|---|
| Outcome integration category | Cerebrovascular outcomes only | CVO | Study reported at least one cerebrovascular, vascular, or brain-related outcome relevant to brain health and did not report a direct cognitive outcome. | Used to identify vascular-only or brain-outcome-only exercise intervention studies. |
| Outcome integration category | Cognitive outcomes only | COG | Study reported at least one cognitive outcome and did not report a direct cerebrovascular or vascular outcome. | Used to identify cognition-only exercise intervention studies. |
| Outcome integration category | Both cerebrovascular and cognitive outcomes | BOTH | Study reported at least one cerebrovascular, vascular, or brain-related outcome and at least one cognitive outcome within the same intervention design. | Used to identify studies that assessed vascular or brain-related adaptation alongside cognitive outcomes within the same intervention design. |
| Direction of findings | Positive | POS | Relevant outcomes showed clear improvement after exercise training. | Coded descriptively to support interpretation of study-level findings, but not used as a primary visual encoding variable in Figure 3. |
| Direction of findings | Mixed | MIX | Some outcomes improved, whereas others were null, inconsistent, or domain-specific. | Coded descriptively to identify heterogeneous findings. |
| Direction of findings | Null | NULL | No meaningful improvement was reported for the relevant outcome. | Coded descriptively to identify null findings. |

| Coding Domain | Coding Category | Code | Definition / Coding Rule | Use in Evidence Map |
|---|---|---|---|---|
| Direction of findings | Negative | NEG | Outcomes worsened or changed in an unfavorable direction. | Coded descriptively when negative findings were reported. |
| Direction of findings | Unclear | UNC | Reporting was insufficient or ambiguous, preventing clear classification of the direction of findings. | Used when the direction of findings could not be determined. |
| Evidence density | Low | LOW | One to two studies were present in the exercise modality by outcome-domain cell. | Represented by smaller bubble size and lighter shading in Figure 3. |
| Evidence density | Moderate | MOD | Three to five studies were present in the exercise modality by outcome-domain cell. | Represented by intermediate bubble size and shading in Figure 3. |
| Evidence density | High | HIGH | Six or more studies were present in the exercise modality by outcome-domain cell. | Represented by larger bubble size and darker shading in Figure 3. |
| Methodological and reporting maturity | Higher methodological and reporting maturity | Higher MRM | Randomized controlled trial with an appropriate comparator, clear exercise prescription, adherence reporting, and relevant outcome assessment. | Coded descriptively to contextualize the evidence map, but not used as a primary visual encoding variable in Figure 3. |
| Methodological and reporting maturity | Moderate methodological and reporting maturity | Moderate MRM | Controlled, quasi-experimental, or pilot study with partial reporting or some methodological limitations. | Coded descriptively to support methodological interpretation. |
| Methodological and reporting maturity | Preliminary methodological and reporting | Preliminary MRM | Single-arm, feasibility, or pre-post study with limited control of bias. | Coded descriptively to identify preliminary evidence. |

| Coding Domain | Coding Category | Code | Definition / Coding Rule | Use in Evidence Map |
|---|---|---|---|---|
| | maturity | | | |
| Exercise dose reporting quality | Complete FITT | FITT-C | Frequency, intensity, time, type, progression, and supervision were clearly described. | Used to assess completeness of exercise prescription reporting and replicability. |
| Exercise dose reporting quality | Partial FITT | FITT-P | Frequency, time, and type were reported, but intensity, progression, supervision, adherence, or other key prescription details were incomplete. | Used to identify partial exercise prescription reporting. |
| Exercise dose reporting quality | Limited FITT | FITT-L | Exercise prescription was insufficiently described for replication or interpretation of exercise dose. | Used to identify major gaps in exercise prescription reporting. |

**Table note:** This supplementary table provides the full evidence map coding framework, operational definitions, and decision rules used for the 54 studies included in the scoping review. Original study-level outcome labels were retained during data charting, while closely related cerebrovascular, vascular, brain-related, and cognitive outcomes were grouped into broader domains for Figure 3 visualization to improve interpretability and reduce excessive fragmentation. Exercise-based lifestyle and rehabilitation studies were coded separately when structured exercise was a central intervention component. Methodological and reporting maturity and direction-of-findings categories were coded descriptively and should not be interpreted as formal risk-of-bias ratings or pooled effectiveness estimates.

## Study-level descriptive coding results

The following study-level coding table was added to make the distribution of methodological and reporting maturity and FITT reporting quality transparent and traceable. Methodological and reporting maturity categories are descriptive evidence-map indicators and should not be interpreted as formal risk-of-bias ratings or pooled quality scores.

### Summary distribution

| Coding domain | Category | Number of study reports |
|---|---|---|
| Methodological and reporting maturity | Higher MRM | 26 |
| Methodological and reporting maturity | Moderate MRM | 22 |
| Methodological and reporting maturity | Preliminary MRM | 6 |
| Exercise dose reporting quality | FITT-C | 26 |
| Exercise dose reporting quality | FITT-P | 24 |
| Exercise dose reporting quality | FITT-L | 4 |

### Study-level coding table

| No. | Ref. | Study | Methodological and reporting maturity | FITT reporting quality |
|---|---|---|---|---|
| 1 | 14 | Suzuki et al., 2013 | Higher MRM | FITT-P |
| 2 | 15 | Sungkarat et al., 2018 | Higher MRM | FITT-P |
| 3 | 21 | Tomoto et al., 2021 | Higher MRM | FITT-C |
| 4 | 22 | Tomoto et al., 2021 | Higher MRM | FITT-C |
| 5 | 23 | Thomas et al., 2020 | Higher MRM | FITT-C |
| 6 | 24 | Alfini et al., 2019 | Preliminary MRM | FITT-P |

| No. | Ref. | Study | Methodological and reporting maturity | FITT reporting quality |
|---|---|---|---|---|
| 7 | 25 | Tarumi et al., 2019 | Higher MRM | FITT-C |
| 8 | 26 | Bliss et al., 2022 | Moderate MRM | FITT-P |
| 9 | 27 | Tarumi et al., 2022 | Higher MRM | FITT-C |
| 10 | 28 | Chapman et al., 2013 | Higher MRM | FITT-C |
| 11 | 29 | Penukonda et al., 2025 | Higher MRM | FITT-C |
| 12 | 30 | Sugawara et al., 2026 | Higher MRM | FITT-C |
| 13 | 31 | Kunieda et al., 2022 | Moderate MRM | FITT-P |
| 14 | 32 | Odano et al., 2022 | Moderate MRM | FITT-P |
| 15 | 33 | Boku et al., 2022 | Preliminary MRM | FITT-L |
| 16 | 34 | So et al., 2024 | Moderate MRM | FITT-P |
| 17 | 35 | Hamasaki et al., 2019 | Moderate MRM | FITT-P |
| 18 | 36 | Mitchell et al., 2022 | Moderate MRM | FITT-P |
| 19 | 37 | Akazawa et al., 2012 | Preliminary MRM | FITT-P |
| 20 | 38 | Zhu et al., 2022 | Higher MRM | FITT-C |
| 21 | 39 | Baker et al., 2010 | Moderate MRM | FITT-C |
| 22 | 40 | Zhu et al., 2018 | Higher MRM | FITT-C |
| 23 | 41 | Suzuki et al., 2012 | Higher MRM | FITT-C |
| 24 | 42 | Law et al., 2014 | Moderate MRM | FITT-P |
| 25 | 43 | Law et al., 2019 | Higher MRM | FITT-C |
| 26 | 44 | Li et al., 2022 | Higher MRM | FITT-C |

| No. | Ref. | Study | Methodological and reporting maturity | FITT reporting quality |
|---|---|---|---|---|
| 27 | 45 | De Wit et al., 2018 | Moderate MRM | FITT-P |
| 28 | 46 | Shimada et al., 2018 | Higher MRM | FITT-C |
| 29 | 47 | Bae et al., 2020 | Moderate MRM | FITT-P |
| 30 | 48 | Thapa et al., 2020 | Moderate MRM | FITT-P |
| 31 | 49 | Park et al., 2019 | Higher MRM | FITT-P |
| 32 | 50 | McEwen et al., 2018 | Higher MRM | FITT-C |
| 33 | 51 | Sungkarat et al., 2017 | Higher MRM | FITT-C |
| 34 | 52 | Chen et al., 2023 | Higher MRM | FITT-C |
| 35 | 53 | Grzenda et al., 2024 | Moderate MRM | FITT-P |
| 36 | 54 | Chobe et al., 2022 | Moderate MRM | FITT-P |
| 37 | 55 | Wu et al., 2025 | Higher MRM | FITT-C |
| 38 | 56 | Yoon et al., 2018 | Moderate MRM | FITT-P |
| 39 | 57 | Mavros et al., 2017 | Higher MRM | FITT-C |
| 40 | 58 | Langoni et al., 2019 | Higher MRM | FITT-C |
| 41 | 59 | Kušleikienė et al., 2025 | Moderate MRM | FITT-C |
| 42 | 60 | Krootnark et al., 2024 | Higher MRM | FITT-C |
| 43 | 61 | Huang et al., 2025 | Moderate MRM | FITT-C |
| 44 | 62 | Liao et al., 2021 | Moderate MRM | FITT-P |
| 45 | 63 | Jhaveri et al., 2023 | Preliminary MRM | FITT-P |
| 46 | 64 | Saeed et al., 2024 | Moderate MRM | FITT-L |

| No. | Ref. | Study | Methodological and reporting maturity | FITT reporting quality |
|---|---|---|---|---|
| 47 | 65 | Shimada et al., 2018 | Moderate MRM | FITT-P |
| 48 | 66 | Bisbe et al., 2020 | Moderate MRM | FITT-P |
| 49 | 67 | de Oliveira Silva et al., 2019 | Higher MRM | FITT-C |
| 50 | 68 | Jeong et al., 2021 | Moderate MRM | FITT-P |
| 51 | 69 | Cox et al., 2019 | Higher MRM | FITT-C |
| 52 | 70 | Smith et al., 2022 | Moderate MRM | FITT-P |
| 53 | 71 | Alosco et al., 2014 | Preliminary MRM | FITT-L |
| 54 | 72 | Stanek et al., 2011 | Preliminary MRM | FITT-L |

Note. Higher MRM = high methodological and reporting maturity; Moderate MRM = moderate methodological and reporting maturity; Preliminary MRM = preliminary methodological and reporting maturity; FITT-C = complete FITT reporting; FITT-P = partial FITT reporting; FITT-L = limited FITT reporting. The study-level classifications were generated according to the operational definitions in this supplementary table and are intended to support transparent evidence mapping.
